# Questioning inbreeding: Could outbreeding affect productivity in the North African catfish in Thailand?

**DOI:** 10.1371/journal.pone.0302584

**Published:** 2024-05-06

**Authors:** Chananya Patta, Thitipong Panthum, Chadaphon Thatukan, Wongsathit Wongloet, Piangjai Chalermwong, Pish Wattanadilokchatkun, Thanyapat Thong, Phanitada Srikampa, Worapong Singchat, Syed Farhan Ahmad, Kantika Noito, Ryan Rasoarahona, Ekaphan Kraichak, Narongrit Muangmai, Satid Chatchaiphan, Kednapat Sriphairoj, Sittichai Hatachote, Aingorn Chaiyes, Chatchawan Jantasuriyarat, Visarut Chailertlit, Warong Suksavate, Jumaporn Sonongbua, Jiraboon Prasanpan, Sunchai Payungporn, Kyudong Han, Agostinho Antunes, Prapansak Srisapoome, Akihiko Koga, Prateep Duengkae, Yoichi Matsuda, Uthairat Na-Nakorn, Kornsorn Srikulnath

**Affiliations:** 1 Animal Genomics and Bioresource Research Unit (AGB Research Unit), Faculty of Science, Kasetsart University, Bangkok, Thailand; 2 Sciences for Industry, Faculty of Science, Kasetsart University, Bangkok, Thailand; 3 Special Research Unit for Wildlife Genomics (SRUWG), Department of Forest Biology, Faculty of Forestry, Kasetsart University, Bangkok, Thailand; 4 Department of Botany, Faculty of Science, Kasetsart University, Bangkok, Thailand; 5 Department of Fishery Biology, Faculty of Fisheries, Kasetsart University, Bangkok, Thailand; 6 Department of Aquaculture, Faculty of Fisheries, Kasetsart University, Bangkok, Thailand; 7 Faculty of Natural Resources and Agro-Industry, Kasetsart University Chalermphrakiat Sakon Nakhon Province Campus, Sakon Nakhon, Thailand; 8 School of Agriculture and Cooperatives, Sukhothai Thammathirat Open University, Nonthaburi, Thailand; 9 Department of Genetics, Faculty of Science, Kasetsart University, Bangkok, Thailand; 10 Pathum Thani Aquatic Animal Genetics Research and Development Center, Aquatic Animal Genetics Research and Development Division, Department of Fisheries, Pathum Thani, Thailand; 11 Faculty of Interdisciplinary Studies, Khon Kaen University, Nong Kom Ko, Mueang Nong Khai District, Nong Khai, Thailand; 12 Kalasin Fish Hatchery Farm (Betagro), Buaban, Yangtalad District, Kalasin, Thailand; 13 Research Unit of Systems Microbiology, Department of Biochemistry, Faculty of Medicine, Chulalongkorn University, Bangkok, Thailand; 14 Department of Microbiology, Dankook University, Cheonan, Korea; 15 Bio-Medical Engineering Core Facility Research Center, Dankook University, Cheonan, Korea; 16 Smart Animal Bio institute, Dankook University, Cheonan, Republic of Korea; 17 CIIMAR/CIMAR, Interdisciplinary Centre of Marine and Environmental Research, University of Porto, Terminal de Cruzeiros do Porto de Leixões, Porto, Portugal; 18 Department of Biology, Faculty of Sciences, University of Porto, Porto, Portugal; 19 Center for Advanced Studies in Tropical Natural Resources, National Research University-Kasetsart University, Kasetsart University, Bangkok, Thailand; Tamil Nadu Dr J Jayalalithaa Fisheries University, INDIA

## Abstract

The North African catfish (*Clarias gariepinus*) is a significant species in aquaculture, which is crucial for ensuring food and nutrition security. Their high adaptability to diverse environments has led to an increase in the number of farms that are available for their production. However, long-term closed breeding adversely affects their reproductive performance, leading to a decrease in production efficiency. This is possibly caused by inbreeding depression. To investigate the root cause of this issue, the genetic diversity of captive North African catfish populations was assessed in this study. Microsatellite genotyping and mitochondrial DNA D-loop sequencing were applied to 136 catfish specimens, collected from three populations captured for breeding in Thailand. Interestingly, extremely low inbreeding coefficients were obtained within each population, and distinct genetic diversity was observed among the three populations, indicating that their genetic origins are markedly different. This suggests that outbreeding depression by genetic admixture among currently captured populations of different origins may account for the low productivity of the North African catfish in Thailand. Genetic improvement of the North African catfish populations is required by introducing new populations whose origins are clearly known. This strategy should be systematically integrated into breeding programs to establish an ideal founder stock for selective breeding.

## Introduction

Aquaculture, whose vital role in food and nutritional security is globally recognized, has an essential implication in rural development, in line with the United Nations Sustainable Development Goals. The cultivation of clariid catfish (*Clarias* spp), primarily the North African catfish (*Clarias gariepinus*), is practiced in more than 55 countries worldwide [1, 2]. High adaptability to diverse environments in this species is demonstrated by their wide geographical distribution, ability to tolerate low water quality, and resistance to various infectious agents [1]. The North African catfish show faster growth rates than the bighead catfish (*Clarias macrocephalus*) and walking catfish (*Clarias batrachus*), ultimately achieving significantly larger body sizes [3–7]. For this reason, the North African catfish have been widely farmed both within and beyond their native ranges, contributing to a global production of 1,249,000 tons in 2020 [2]. Intensive translocations of the North African catfish have occurred, primarily within Africa and Asia [8–10]. In Southeast Asia, the North African catfish is not favored by consumers due to its unfavorable color and texture of meat [3, 11]. Consequently, in 1987, hybrid catfish were developed through artificial crossbreeding of male North African and female bighead catfish to increase productivity and meat quality [12, 13]. A rapid growth rate and increased disease resistance were obtained in the F_1_ hybrid catfish. This, in turn, led to the widespread cultivation of these hybrids, which constituted over 90% of catfish production in Thailand by 2004 [3, 14]. However, mass production of F_1_ hybrids has been restricted by sterility, which is caused by interspecific reproductive isolation [15–17]. Hence, preparation of parental genetic stocks of both species is necessary for hybrid production on all farms. Critically, over the past decade, a decline in survival, growth, low conception, and fertility rates has been observed in North African catfish populations in Thailand. This reduction in the productive efficiency may be primarily attributable to the low quality of breeding populations of the North African catfish [11, 14, 18–20]. The long-term breeding using closed stocks of the North African catfish may have admittedly resulted in a loss of genetic variation in descendant populations [20]. In Thailand, over three different population stocks of the North African catfish are believed to have been introduced; however, their origins and breeding process are unknown because of the absence of breeding record [10, 11, 20]. Genetic variations decrease in the breeding stocks that passed through many generations in closed populations, resulting in the reduced growth performance, fertility, and viability in descendant populations caused by inbreeding depression [19–22].

In small closed populations, inbreeding due to mating between genetically related individuals is inevitable even if it is a random mating in either natural populations or artificially bred population [22]. The increase in homozygosity during inbreeding results in loss of fitness, as it eliminates heterozygous advantages and reveals deleterious recessive genes. Given the limited genetic diversity observed in the North African catfish populations in Thailand [10, 19, 23], it is hypothesized that a high level of inbreeding has occurred, resulting in inbreeding depression that causes reduced conception rates and fertility. To effectively manage breeding stocks of the North African catfish and recover their productivity, it is crucial to investigate the genetic divergence of the cultured populations. Despite their potential significance as a source for genetic improvement, the genetic diversity within the breeding populations has been scarcely explored [10, 19, 23]. Random variations in genetic diversity and alleles are frequently induced by the introduction of undocumented individuals into the North African catfish farms in Thailand, typically through commercial human-mediated transfers [10]. Genetic monitoring of North African catfish should be conducted every 4–5 years to facilitate sustainable aquaculture management.

In this study, the genetic divergence and population structure in the farmed North African catfish populations were assessed using mitochondrial DNA (mtDNA) D-loop sequencing and microsatellite genotyping. Microsatellite genotyping was previously conducted using a limited number of loci (fewer than eleven), which could potentially cause bias in estimating genetic divergence [24]. Furthermore, considering that genetic diversity can evolve over time depending how stocks are managed. this study sought to verify the existence of genetically distinct North African catfish populations in Thailand and evaluate the genetic divergence of the populations. The aim of the current study is to establish a genetic database for North African catfish populations to support a systematic program, potentially revising previous study findings [10, 19, 23]. This information will be valuable for developing the management of the breeding populations of the North African catfish and optimizing their genetic improvement programs.

## Materials and methods

### Specimen collection and DNA extraction

A total of 136 captive individuals of the North African catfish were collected from three locations, Sing Buri (SBR: 14°58’13"N, 100°18’41"E) (N = 8), Kalasin (KSN: 16°39’29"N, 103°29’10"E) (N = 97), and Nakhon Nayok (NYK: 1**4**°**05**’**58**"N, 10**1**°**0**9’**39**"E) (N = 31), which srve as centers for supplying the breeding stocks to farms across the country. The three farms analyzed for the genetic diversity of North African catfish were not the same as those used in previous studies [10, 19, 23]. Permission was granted by the owners and all individuals were released immediately after collecting samples. Notably, reliable genetic diversity estimates were demonstrated with small samples [25]. Thus, specimen division by unequal sample sizes may not significantly affect results in this study. All animal care and experimental procedures were approved by the Animal Experiment Committee of Kasetsart University (approval no. ACKU65-SCI-003 and ACKU65-SCI-026), and conducted in accordance with the Regulations on Animal Experiments at Kasetsart University. Approximately 0.3 × 0.3 cm of the caudal fins was taken from each individual and stored in 95% ethanol at 4°C. Genomic DNA was extracted using the standard salting-out protocol described by Supikamolseni et al. [26]. DNA quantity and quality were determined using a NanoDrop 2000 Spectrophotometer (Thermo Fisher Scientific, Wilmington, DE, USA) and 1.0% agarose gel electrophoresis, respectively. Detailed information of specimen used in this study is presented in S1 Table.

### Microsatellite genotyping and data analysis

Fifteen microsatellite primer sets were sourced from Agbebi et al. [27] and Kánainé Sipos et al. [28] (S2 Table). The 5′ end of the forward primer of each primer set was labeled with each of fluorescent dyes (Carboxyfluorescein: 6-FAM, Hexachloro–fluorescein: HEX, and Tetramethylrhodamine: TAMRA) (Macrogen Inc., Seoul, Korea). PCR amplification was singly performed using 15 μL of 1× standard reaction buffer (Apsalagen Co., Ltd., Bangkok, Thailand), 1.75 mM MgCl_2_, 0.2 mM dNTPs, 0.5 μM primers, 0.5 U *Taq* polymerase (Apsalagen Co., Ltd.), and 50 ng genomic DNA. The PCR protocol was as follows: initial denaturation at 95°C for 2 min, followed by 45 cycles of 95°C for 15 s, 55°C for 20 s, and 72°C for 40 s, with a final extension at 72°C for 5 min. The PCR products were detected using electrophoresis on 1% agarose gel. For microsatellite genotyping, fluorescent DNA fragment length analysis was performed using an ABI 3730XL automatic sequencer (Applied Biosystems, Foster City, CA, USA) at the DNA sequencing service of Macrogen Inc. Allelic sizes were determined using Peak Scanner version 1.0 (Applied Biosystems). The genotypic data generated in this study were deposited in the Dryad Digital Repository (https://datadryad.org/stash/share/XjO844ulqFErzDzbcmbCOeDNCzRKBLiO56AVCa-jr28, accessed 02 October 2023).

Genetic diversity was assessed using parameters, including the allelic frequency, number of alleles (*A*), effective number of alleles (*N*_a_), allelic richness (*AR*), number of effective alleles (*N*_ea_), Shannon’s information index (*I*), observed heterozygosity (*H*_o_), expected heterozygosity (*H*_e_), Hardy–Weinberg equilibrium and linkage disequilibrium, fixation index (*F*), and Wright’s *F*-statistic for subpopulations within the total population (*F*_ST_), which were calculated using Arlequin version 3.5 [29]. Considering the population size, deviations from Hardy–Weinberg equilibrium were assessed at each locus. This was examined through the Markov chain Monte Carlo (MCMC) approximation of Fisher’s exact test, utilizing the "genepop" package version 1.2.2 in R version 4.2.0 [30, 31]. Welch’s *t*-test, which does not assume equal variance between samples, was employed to assess significant differences between *H*_o_ and *H*_e_. The "t.test" function in the "stats" package of R version 4.2.0 was used for this analysis [31]. To examine the equality of variances between the *H*_o_ and *H*_e_ values across all captive populations, Bartlett’s test of homogeneity of variances was initially performed,this test utilized the “bartlett.test” function within the “stats” package of R version 4.2.0 [31]. *AR* was calculated using FSTAT version 1.2 [32], and MicroChecker version 2.2.3 was used to identify null alleles [33]. Polymorphic information content (*PIC)* was estimated for each locus using the Excel Microsatellite Toolkit. The *I* and *F* were calculated for each locus in the population using GenAlEx version 6.5 [34]. Effective population size (*N*_e_) was inferred as the number of breeding individuals contributing to the next generation using the linkage disequilibrium (LD) method implemented in NeEstimator v2.0 [35].

Relatedness values (*r*) were calculated for all individual pairs and mean pairwise *r* values based on allelic frequencies in the population were calculated using GenAlEx version 6.5 [34]. Then the distributions of the pairwise *r* values between all pairs were compared. This was achieved through a bootstrap version of the Kolmogorov–Smirnov test, as outlined by Praestgaard et al. [36]. The analysis was performed using the "ks.test" function implemented in the "stats" package of R version 4.2.0 [31]. The same approach was adopted to compare inbreeding coefficients (*F*_IS_) for individuals and overall *F*_IS_ at 95% confidence intervals (CIs) were calculated using the LynchRt estimator [37, 38] implemented in COANCESTRY [39]. The *r* values and *F*_IS_ were examined under the assumption that their averages are not significantly different from random assortments of unrelated individuals. Pairwise genetic distances among populations were calculated using the infinite allele model (IAM) based on *F*_ST_ in Arlequin version 3.5 with corrected *p* values and the stepwise mutation model (SMM) using *R*_ST_ in FSTAT version 1.2 [32]. To consider the possible influence of null alleles on the estimation of genetic differentiation, the Free^NA^ program [40] was run, which provided pairwise *F*_ST_^ENA^ values with ENA correction for null alleles. To elucidate group structure, analysis of molecular variance (AMOVA) was performed using GenAlEx version 6.5 [34]. Unlike *F*_ST_, this algorithm identifies a hierarchical structure of subgroups and does not require an a priori assumption of Hardy–Weinberg equilibrium. Nei’s genetic distances between populations were then examined using GenAlEx version 6.5 [34, 41]. BOTTLENECK version 1.2.02 [42] was used to evaluate signatures of recent population bottlenecks to excess heterozygosity and characteristic shifts in allelic frequency distributions observed in bottlenecked populations. To account for potential biases due to the limited sample size and number of loci, a two-phased mutation model (TPM) and a stepwise mutation model (SMM) were incorporated into the Wilcoxon signed-rank test to assess the significance of excess heterozygosity. Specifically, a 95% single-step mutation rate and a 5% multistep mutation rate were used in the TPM, with a variance of 12 set for multistep mutations [42]. This test detects relatively short-term bottleneck events. Hence, to determine whether the long-term bottleneck event was present during the process of breeding the North African catfish, the *M* ratio test was performed using Arlequin version 3.5 [29]. The *M* ratio, which is calculated as the mean number of alleles divided by the allelic size range, determines if there has been a decline in population in the past or not. The existence of bottleneck events in the past is represented by an *M* ratio lower than 0.68, as indicated by Garza and Williamson [43].

To assess the overall relationships among individuals within the population, principal coordinate analysis (PCoA) was conducted using GenAlEx version 6.5 [34]. Population structure with various gene pools was determined using the model-based clustering method in STRUCTURE version 2.3.4, which was run in parallel using *Structure_threader* [44, 45]. The run-length was set to 100,000 MCMC replicates following a burn-in period of 100,000 generations using a model with correlated allelic frequencies under a straight admixture model. The number of clusters (*K*) varied from 1 to 25 with 15 replicates for each value of *K*. The most probable number of clusters was determined by plotting the log likelihood of the information (ln Pr (*X*|*K*)) across the tested range of *K* values. The optimal *K* value was selected when ln Pr(*X*|*K*) reached a stable state [44]. The Δ*K* strategy was employed using a Structure Harvester to complement the analysis [46].

To examine the occurrence of genetic transfer between captive populations of the North African catfish, we applied two methods for detecting gene flow. Firstly, the recent migration rates between populations were estimated using BayesAss version 3.0.5 [47], which uses MCMC sampling within a Bayesian framework. The MCMC analysis was conducted over a span of 10 million generations, and the first one million generations were discarded for the burn-in period. Samples were collected after every 100 generations. To ensure effective exploration and convergence, the mixing parameters associated with migration rates (*m*), allelic frequencies (*a*), and inbreeding coefficients (*f*) were optimized according to the guidelines recommended by Wilson and Rannala [47]. Posterior acceptance rates between 20% and 60% were targeted for each parameter during the optimization process. The mixing parameters for the analysis were set as *m* = 0.20, *a* = 0.50, and *f* = 0.30. To investigate the historical gene flow, the MIGRATE-N version 4.4.3 [48] was used to perform Bayesian analysis for estimating the migration rate and effective population size of the three populations based on microsatellite data sets and coalescent theory [49]. Uniform prior distributions were used for the basic microsatellite model, and 5,000 steps were recorded every 100 generations using the MCMC procedure. The first 100,000 generations were discarded as burn-ins. Estimates were calculated for the mutation-scaled immigration rate (*M*) and mutation-scaled population size (Θ). The number of individuals entering populations (*N*_m_) was calculated, and the presence of gene flow in the past was determined using the formula *N*_mi–>j_  =  Θ_j_*M_i–>j_/4, where *N*_mi–>j_ represents the effective number of immigrants from population i to population j per generation or the gene flow rate. Circos version 0.69–8 was used to visualize genetic connectivity among populations [50].

### Mitochondrial DNA D-loop sequencing, quality control, and data analysis

MtDNA D-loop fragments were amplified using our designed primer pair CGA_CYTB_D–loop_F1 (5′-TAGTACACATCTGCCGAGAC-3′) and CGA_CYTB_D–loop_R1 (5′-ATGCCAAGTGAAAGTGACC-3′). PCR amplification was performed using 15 μL of 1 × standard reaction buffer, 2.0 mM MgCl_2_, 0.2 mM dNTPs, 0.5 μM primers, 0.5 U *Taq* polymerase (Apsalagen Co., Ltd.) and 50 ng genomic DNA. The PCR conditions were as follows: initial denaturation at 94°C for 5 min, followed by 35 cycles of 94°C for 1 min, 62°C for 1 min, 72°C for 1 min, and a final extension at 72°C for 10 min. The PCR products were purified using the FavorPrep GEL/PCR Purification Mini Kit (Favorgen Biotech Corp., Ping-Tung, Taiwan). The nucleotide sequences of the DNA fragments were determined using the DNA sequencing service of First Base Laboratories Sdn Bhd (Seri Kembangan, Selangor, Malaysia). The BLASTn program (http://blast.ncbi.nlm.nih.gov/Blast.cgi) were used to search nucleotide sequences in the National Center for Biotechnology Information (NCBI) database to confirm the identity of the DNA fragments amplified in the present study. All sequences were deposited in the DNA Data Bank of Japan (DDBJ) (https://www.ddbj.nig.ac.jp/, accessed 03 October 2023) (accession numbers: LC781360–LC781495) (S1 Table).

Multiple alignments of 136 partial mtDNA D-loop sequences from the North African catfish were performed using the default parameters in the Geneious Prime software version 2023.1.2 (Biomatters, Auckland, New Zealand, https://www.geneious.com). All unalignable and gap-containing sites were carefully removed from the datasets. Haplotype diversity (*h*), nucleotide diversity (*π*), number of haplotypes (*H*), the estimator theta (*S*), overall haplotype, and average number of nucleotide differences (*k*) were calculated based on the mtDNA D-loop sequences, as implemented in DnaSP version 6.12.03 [51]. The genetic differentiation coefficient (*G*_ST_), *F*_ST_, *Ф*_*ST*_ values, and *N*_m_ were estimated from the sequence and haplotype data using Arlequin version 3.5 [28]. The *F*_ST_ and *Ф*_ST_ values were calculated by analyzing 1,000 permutations of haplotypes between populations [29]. The variance in haplotype frequencies is solely accounted for by the *F*_ST_ statistic, while the relationships among haplotypes, based on molecular genetic distance, are considered by *Ф*_ST_ [29]. The average number of nucleotide substitutions per site between populations (*D*_xy_) and net nucleotide substitutions per site between populations (*D*_a_) were estimated using DnaSP version 6.12.03 [51]. A statistical parsimony network was constructed from the consensus sequences using the Templeton, Crandall, and Sing (TCS) algorithm implemented in PopART version 1.7 to examine haplotype grouping and population dynamics [52]. The mtDNA D-loop sequences of all individuals were used to construct the haplotype network. Phylogenetic analysis was then performed using Bayesian inference with MrBayes v3.2.7a [53] using *Clarias* sp. (accession number KJ201871), bighead catfish (*C*. *macrocephalus*, accession numbers KF583824 and KF583863), walking catfish (*C*. *batrachus*, accession numbers KC747511, KF583870, and JX423834), and mahseer (*Tor tor*, accession numbers HQ625378 and HQ625381) as outgroups. In the MCMC analysis four chains were run simultaneously for one million generations, sampling every 100 generations. Posterior probabilities within the sampled tree population were determined through Bayesian inference. Coalescent theory served as the framework, with migration rate and effective population size estimated using mtDNA D-loop sequence data analyzed via MIGRATE-N v4.4.3 software [54]. A uniform prior distribution represented the underlying DNA sequence model. In the MCMC procedure, 5,000 recorded steps were used per 100 generations, with the initial 100,000 generations being discarded as burn-in. Estimates for parameters such as *M*, Θ, and the number of effective migrants per generation (*N*_m_, calculated as ΘM/2) were yielded by genetic data [54]. The history of changes in population was also determined using a statistical test of neutrality. Tajima’s D* [55], Fu and Li’s D* and F* [56], and Fu’s *F*s [57] parameters were calculated using Arlequin version 3.5 [29]. The mismatch distribution approach, in which an observed frequency distribution of pairwise nucleotide differences among individuals is compared with expected distributions from an expanding population (small raggedness index) or a stationary population (large raggedness index), was performed to test for genetic signatures of historical population expansion in the North African catfish [58, 59]. Population expansion was estimated using a generalized least-squares approach, and CIs were computed with 10,000 bootstrap replicates. This was implemented in DnaSP version 6.12.03 [51]. Bayesian coalescent-based methods were also applied to evaluate the historical demographic fluctuations, using the extended Bayesian skyline plot (EBSP) analysis implemented in BEAUTi version 2.5.0 (part of the BEAST version 2.5.0 package) [60, 61], by applying the HKY model, strict clock model, and coalescent Bayesian skyline model with a prior Gamma distribution. The prior date was set to 45 million years ago according to the fossil data of African Clariidae [62]. TRACER (version 1.7.1, http://beast.community/tracer, accessed 10 October, 2023) was used to assess the burn-in and the effective sample sizes (ESSs) of the parameters. EBSP can fit different demographic scenarios by allowing for changes in population size over time.

## Results

### Genetic diversity of the North African catfish populations based on microsatellite genotype data

Microsatellite genotyping revealed 121 alleles for 15 loci with an average *N*_a_ value of 6.111±0.356 per locus (Table 1). Most allelic frequencies in the populations deviated significantly from expectations under Hardy–Weinberg equilibrium with the presence of linkage disequilibrium (S3–S5 Tables). Null alleles were frequently observed for six loci (Cg003, Cg214, Cg294, Cg316, Cga01, and Cga03); however, the markers were treated in the same way as other ones. The *F* values of all populations were positive. The *PIC* values of all populations ranged from 0.406 to 0.884, and *I* ranged from 0.796 to 2.371 (S6 Table). The mean *H*_o_ and *H*_e_ values were 0.420±0.032 (mean±standard error (SE) and 0.708±0.015, respectively, whereas the mean *AR* value was 5.841±0.145 (Table 1, S6 Table). Welch’s *t*-test showed significant differences between *H*_o_ and *H*_e_ values in all populations (Table 2). Pairwise comparison of *H*_o_ values did not reveal any statistical differences between populations, while the pairwise *H*_e_ values were significantly different only between the SBR and KSN populations (Table 3). The values of genetic diversity indices are summarized in Table 1 and S6 Table. The *N*_e_ values were infinite for the SBR population, 133.4 for KSN, and 385.9 for NYK (Table 4). The average *F*_IS_ of the SBR, KSN, and NYK populations were 0.097±0.034, 0.049±0.010, and 0.048±0.016, respectively (Table 4), with individual *F*_IS_ values in all populations ranging from −0.134 to 0.338 (S7–S9 Tables). However, the distributions of individual *F*_IS_ values were not significantly different between populations (S1 Fig, S10 Table). A pairwise test to determine the degree of relatedness (*r*) between individuals revealed that the average pairwise *r* values calculated for a total of 5,149 combinations of 136 individuals were −0.101±0.013 for the SBR population, −0.007±0.001 for KSN, and −0.023±0.003 for NYK. The analysis identified 5,120 pairs with −0.25 < *r* < 0.25 and 29 pairs with 0.25 > *r* (Table 4 and S11–S13 Tables). The distribution was highly skewed to the left, indicating lower pairwise *r* values than expected ones.

**Table 1:** Genetic diversity in three populations of the North African catfish (*Clarias gariepinus*) estimated using 15 microsatellite loci. Detailed information of 136 individuals analyzed in this study is presented in S1 Table.

Population*		N	*N*_a_	*AR*	*N*_ea_	*I*	*H*_o_	*H*_e_	*M-ratio*	*PIC*	*F*
SBR	Mean	7.400	4.267	3.555	3.102	1.231	0.370	0.655	0.258	0.604	0.446
	S.E.	0.321	0.267	0.149	0.207	0.065	0.066	0.025	0.047	0.027	0.100
KSN	Mean	95.133	7.600	7.565	4.490	1.614	0.445	0.755	0.321	0.718	0.422
	S.E.	0.920	0.542	0.498	0.394	0.079	0.053	0.019	0.039	0.023	0.063
NYK	Mean	30.467	6.467	6.404	4.122	1.508	0.444	0.715	0.366	0.679	0.390
	S.E.	0.274	0.646	0.596	0.494	0.098	0.048	0.029	0.040	0.031	0.055
all population	Mean	44.333	6.111	5.841	3.905	1.451	0.420	0.708	0.315	0.667	0.419
	S.E.	5.608	0.356	0.145	0.234	0.052	0.032	0.015	0.003	0.002	0.043

N, Sample size; *N*_a_, number of alleles; *AR*, allelic richness; *N*_ea_, number of effective alleles; *I*, Shannon’s information index; *H*_o_, observed heterozygosity; *H*_e_, expected heterozygosity; *PIC*, polymorphic information content; *F*, fixation index.

*SBR, Sing Buri; KSN, Kalasin; NYK, Nakhon Nayok.

**Table 2:** Observed (*H*_o_) and expected (*H*_e_) heterozygosity in three populations of the North African catfish (*Clarias gariepinus*) estimated by the Welch’s *t*-test using 15 microsatellite loci.

Population*	*H*_o_	*H*_e_	df	*t*-test	*p*-value
SBR	0.370±0.066	0.655±0.025	-0.285	-4.038	0.003
KSN	0.445±0.053	0.755±0.019	-0.310	-5.506	0.000
NYK	0.444±0.048	0.715±0.029	-0.271	-4.832	0.000

*SBR, Sing Buri; KSN, Kalasin; NYK, Nakhon Nayok.

**Table 3:** Comparison of observed (*H*_o_) and expected (*H*_e_) values between three populations of the North African catfish (*Clarias gariepinus*) based on the genotyping data of 15 microsatellite loci.

	Population 1*	Population 2*	df	SE	*t*-test	*p*-value
Observed heterozygosity (*H*_o_)	SBR	KSN	-0.075	0.085	-0.886	0.387
	SBR	NYK	-0.074	0.082	-0.907	0.379
	KSN	NYK	0.001	0.072	0.014	0.989
Expected heterozygosity (*H*_e_)	SBR	KSN	-0.100	0.031	-3.184	0.005
	SBR	NYK	-0.060	0.038	-1.567	0.129
	KSN	NYK	0.040	0.035	1.154	0.253

* SBR, Sing Buri; KSN, Kalasin; NYK, Nakhon Nayok.

**Table 4:** Inbreeding coefficients, relatedness values and effective population size in three populations of the North African catfish (*Clarias gariepinus*).

Population*	N	*F*_IS_	Relatedness (*r*)	Estimated *N*_e_	95% CIs for *N*_e_
SBR	8	0.097±0.034	-0.101±0.013	Infinite	22.8–Infinite
KSN	97	0.049±0.010	-0.007±0.001	133.4	99.6–192.5
NYK	31	0.048±0.016	-0.023±0.003	385.9	103.9–Infinite

N, sample size; *F*_IS_, inbreeding coefficient; *N*_e_, effective population size.

* SBR, Sing Buri; KSN, Kalasin; NYK, Nakhon Nayok.

**Fig 1:**
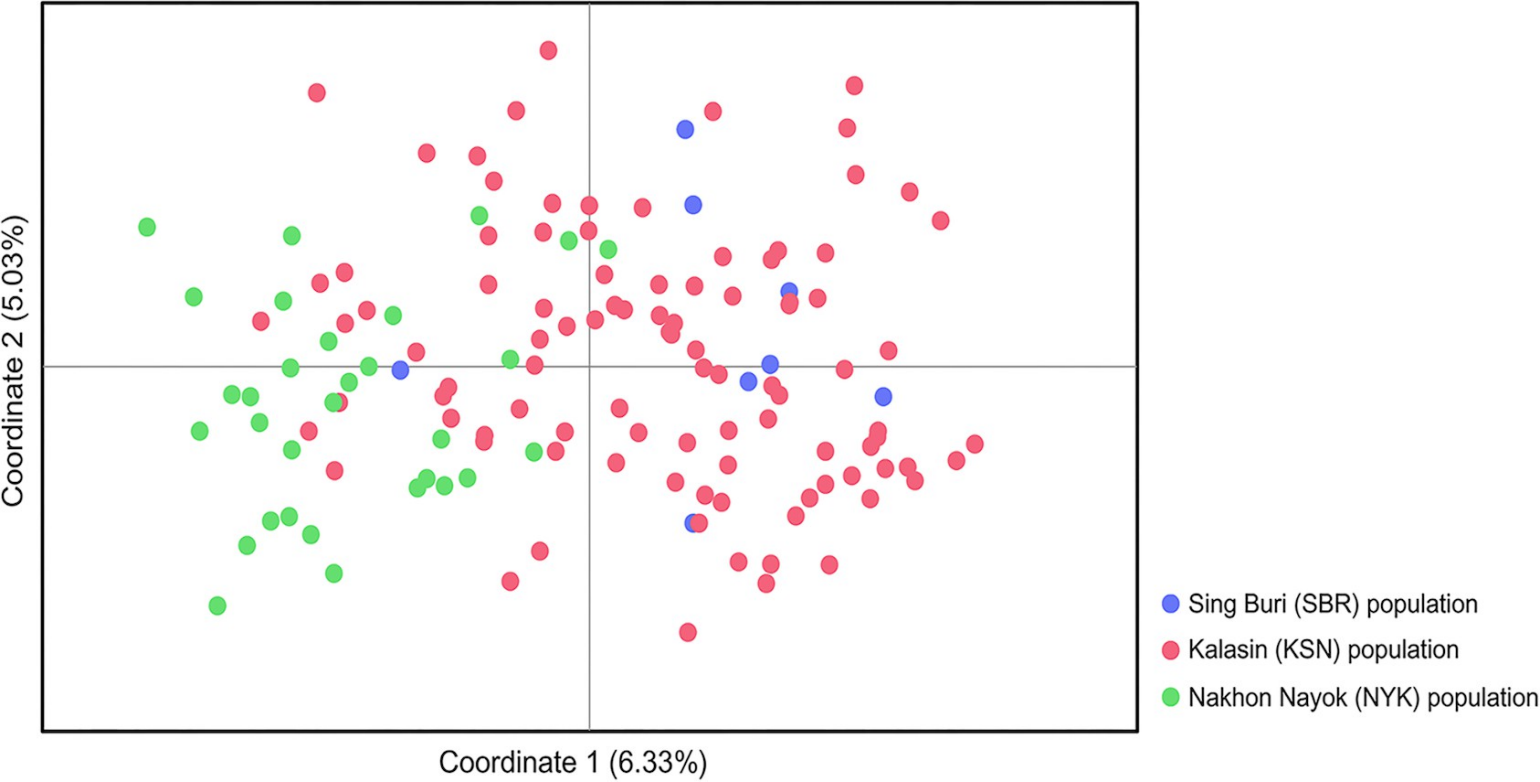
Genetic structures of three populations of the North African catfish revealed by the principal coordinate analysis.

**Fig 2:**
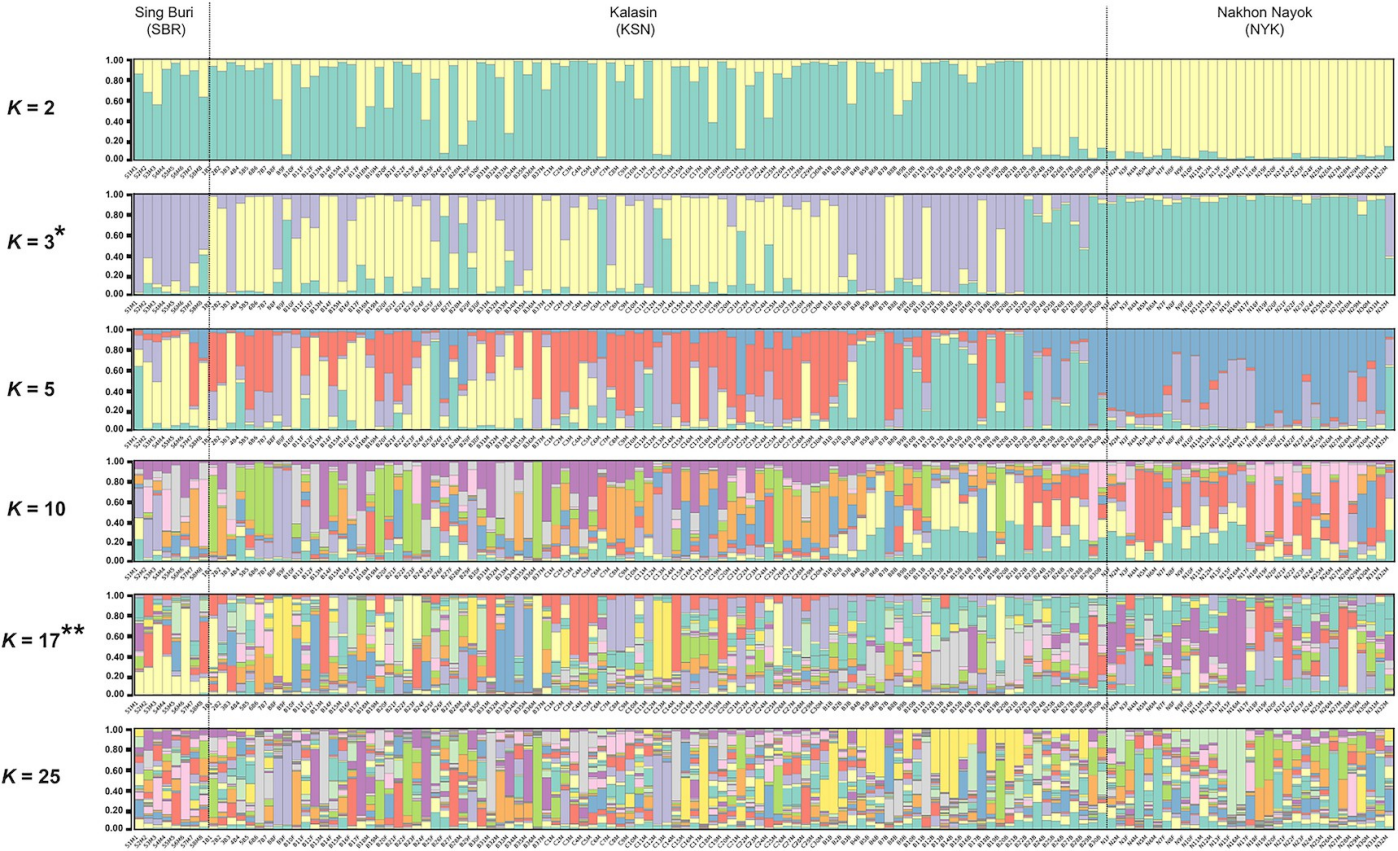
Genetic structures of three populations of the North African catfish revealed by the Bayesian structural analysis. Each vertical bar on the *x*-axis represents an individual, and the *y*-axis represents the proportion of membership in each genetic cluster. All individuals from three populations are superimposed on the plot. Black vertical lines indicate the boundaries. The highest posterior probability, denoted by *, is determined based on Evanno’s Δ*K*, and the ln P(*K*) is represented by **.

**Fig 3:**
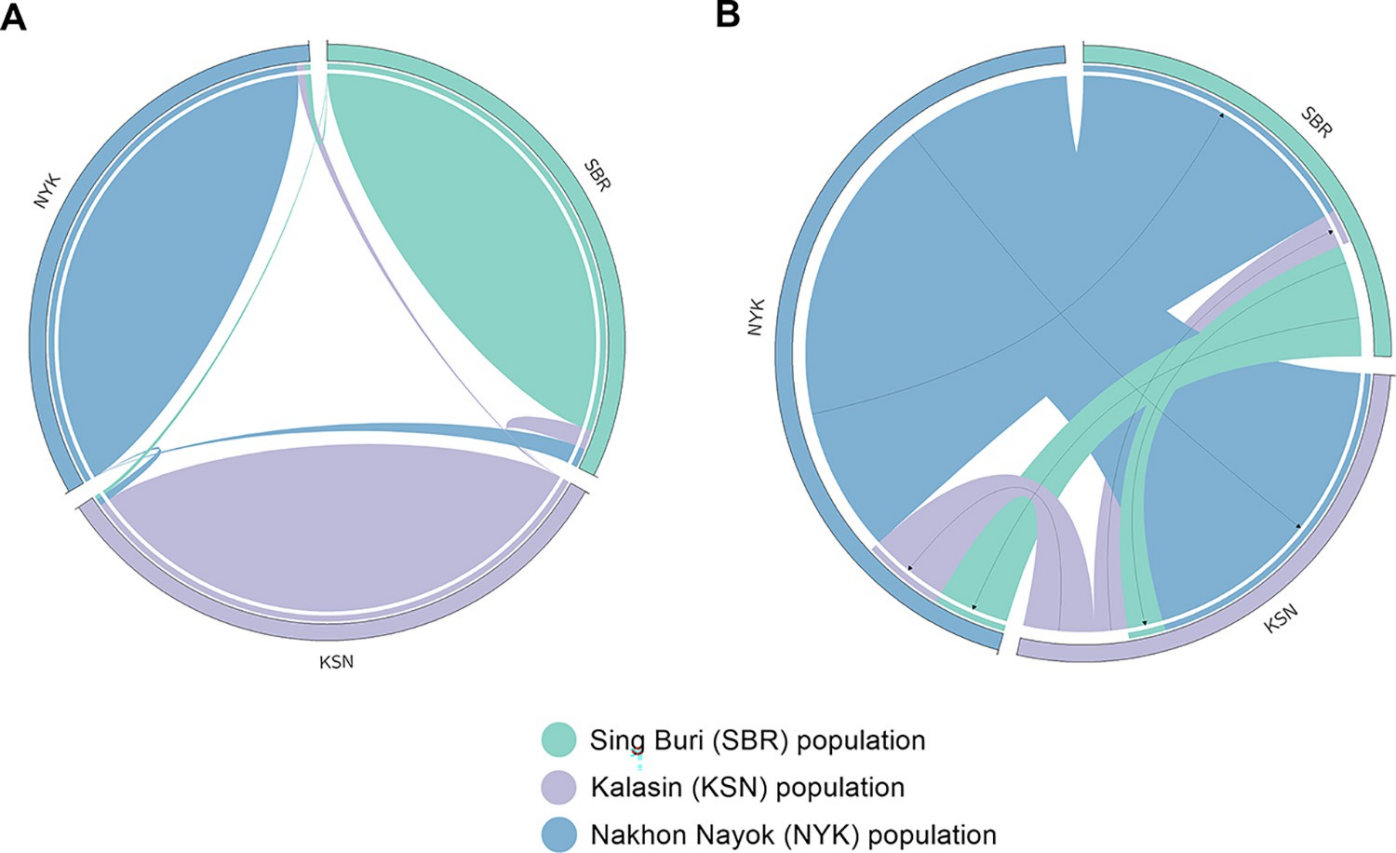
The source–sink migration dynamics revealed by Circos. (A) The current migration directionality estimated using the BayesAss. (B) The historical migration represented using the MIGRATE-N. The width of the migration curves indicates the relative magnitude of migration.

Significant differences (*p* < 0.05) were found in the *F*_ST_ values between the SBR and KSN populations after 110 permutations (S14 Table). The AMOVA with 15 microsatellite loci showed variation were distributed 40% between individuals and 6% between populations (S15 Table). Nei’s genetic distances indicated that KSN was closer to NYK than SBR (S16 Table). The PCoA indicated that all individuals tended to mix together (Fig 1). Different gene pool patterns between the populations were observed by the model-based Bayesian algorithms implemented in STRUCTURE with the increase in *K* values (Fig 2). The highest posterior probability was found at *K* = 3 as a single peak based on the Evanno’s Δ*K*, while the mean ln P(*K*) showed a single peak at *K* = 17 (S2 Fig). The Wilcoxon signed-rank test was performed to find out if bottlenecks existed in the three populations, and consequently SMM and TPM values ranged from 0.555 to 0.906 and from 0.000 to 0.013, respectively, which showed a normal L-shaped mode shift, indicating that there were no bottlenecks at least in recent years (S17 Table). The *M* ratios of the SBR, KSN, and NYK populations were 0.258, 0.321, and 0.366, respectively (Table 1). These *M* ratios were much lower than 0.68 threshold estimated by Garza and Williamson [43], indicating that rapid population decreases occurred in these population in the historical past. The migration rate determined by BayesAss, which serves as an index to estimate recent gene flow, ranged from 0.930 to 0.981 within populations and from 0.006 to 0.035 between populations (Fig 3 and S18 Table). MIGRATE-N analysis using the datasets of microsatellite genotypes revealed a diverse range of *M* values (12.333–121.667); the highest *M* value (121.667) was observed from NYK to KSN (S19 Table). The median scaled mutation rates (Θ) ranged from 0.096 (NYK) to 0.098 (SBR and KSN) (S19 Table). A diverse range of *N*_m_ values from 0.302 to 2.992 was observed among the three populations. The highest *N*_m_ value (2.992) was observed from NYK to KSN, indicating that relatively higher gene flow occurred between these populations (S20 Table).

### Genetic diversity of the North African catfish populations based on mitochondrial DNA D-loop sequence data

The aligned amplicon length of the mtDNA D-loop sequences was 451 bp. The numbers of haplotypes in the SBR, KSN, and NYK populations and the entire population were 4, 9, 10, and 17, respectively. The values of *h* and *π* were 0.665±0.026 and 0.053±0.003, respectively for the entire population (Table 5). The values ranged from −0.020 to 0.032 for *F*_ST_, 0.007 to 0.057 for *G*_ST_, 0.005 to 0.031 for *Φ*_ST_, 0.049 to 0.061 for *D*_xy_, –0.002 to 0.001 for *D*_a_, and 15.372 to infinite for *N*_m_, (Table 6). A haplotype network was constructed with the data of polymorphic sites and haplotypes (S3 Fig). The two most common haplotypes (CGA4 and CGA13) were shared among the three populations. The phylogenetic analysis of mtDNA D-loop sequences from the 136 individuals indicated that the individuals from the three populations were closely related to each other and grouped into the same cluster, which was separated from those of other species (S4 Fig). The MIGRATE-N analysis revealed that the posterior probability distribution for each parameter was also well defined (S21 Table), thus facilitating the generation of point estimates and credibility intervals for each parameter. *M* values ranged from 3.70 to 139.00, with the highest value (139.00) that was observed in the migration from KSN into NYK. Θ values of 0.098 were observed in all the populations (S21 Table). A diverse range of *N*_m_ values (0.091 to 3.416) was observed, with the highest value (3.416) that was observed in the migration from KSN into NYK (S22 Table). Population history was assessed using multiple tests of neutrality based on mtDNA D-loop sequence analysis between populations. The results revealed non-significant values for Tajima’s *D* (ranging from −0.583 to 1.583), Fu and Li’s F* (−0.494 to 1.044), and Fu and Li’s *D* (−2.595 to 0.872) (Table 7). Mismatch distribution analysis revealed unimodal distribution for the KSN and NYK populations, while SBR showed the bimodal distribution (S5 Fig). The raggedness index values ranging from 0.214 to 0.542 were not statistically significant (Table 7). Constant population size was determined using an mtDNA D-loop sequence-based model (S6 Fig).

**Table 5:** The genetic diversity of mitochondrial DNA D-loop sequences in three populations of the North African catfish (*Clarias gariepinus*).

Population*	N	Number of haplotypes (*H*)	Theta (Per Site) from *S*	Average number of nucleotide differences (*k*)	Overall haplotype	Nucleotide diversities (*π*)
SBR	8	4	0.048	24.786	0.643± 0.184	0.056±0.013
KSN	97	9	0.033	21.360	0.625± 0.028	0.048±0.001
NYK	31	10	0.081	29.714	0.753±0.066	0.067±0.012
all populations	136	17	0.067	23.172	0.665± 0.026	0.053± 0.003

* SBR, Sing Buri; KSN, Kalasin; NYK, Nakhon Nayok.

**Table 6:** Genetic differentiation of the mitochondrial DNA D-loop sequences between three populations of the North African catfish (*Clarias gariepinus*).

Population 1*	Population 2*	*G*_ST_	*Ф*_ST_	*F*_ST_	*D*_xy_	*D*_a_	*N*_m_
NYK	SBR	0.057	0.031	0.032ns	0.061	0.001	15.372
NYK	KSN	0.007	0.012	0.011ns	0.057	0.000	46.637
SBR	KSN	0.034	0.005	-0.020ns	0.049	-0.002	Infinite

*G*_ST_, genetic differentiation coefficient; *Ф*_ST_, correlation of random haplotypes within populations; *F*_ST_, Wright’s *F*-statistics for subpopulations within the total population; *D*_xy_, average number of nucleotide substitutions per site between populations; *D*_a_, net nucleotide substitutions per site between populations; *N*_m_, number of individuals entering populations.

* SBR, Sing Buri; KSN, Kalasin; NYK, Nakhon Nayok.

**Table 7:** Neutrality tests of mitochondrial D-loop sequences in three populations of the North African catfish (*Clarias gariepinus*).

Population^§^	Tajima	Fu *D*	Fu *F*	Fu’s *F*_*s*_	Raggedness Index
SBR	1.145^ns^	0.872^ns^	1.044^ns^	7.558	0.260^ns^
KSN	1.583^ns^	-1.825^ns^	-0.494^ns^	29.987	0.542^ns^
NYK	-0.305^ns^	-0.095^ns^	-0.197^ns^	13.935	0.214^ns^
all populations	-0.583^ns^	-2.595*	-2.019^ns^	22.608	0.339^ns^

ns, not significant

*, *p* < 0.05.

^**§**^SBR, Sing Buri; KSN, Kalasin; NYK, Nakhon Nayok.

## Discussion

To overcome the genetic vulnerability of the North African catfish in the aquaculture farms in Thailand, the initiatives for their genetic improvement began in 2016 by the government and private sector, often using the stocks introduced from the captive populations in the past three decades [10]. The present breeding stocks in Thailand are believed to have originated from the populations that were introduced into Vietnam from Central Africa in 1974 [63]. They were introduced into Thailand in 1987 and then have contributed to aquaculture production [19]. This aligns with our demographic analysis by microsatellite genotyping, which revealed a historical population reduction in all examined captive populations. Consistently, a non–significant raggedness index was observed and most captive North African catfish populations showed unimodal distributions, indicating potential population expansion after reduction in the three populations in the past. Similarly, maternal lineage analyses with mtDNA D-loop sequences revealed low π and *h* values in all populations. This suggests that maternal gene flow from other populations into the breeding populations were well restricted during the process of introducing captured North African catfish. By contrast, no recent bottlenecks were detected in the population, as evidenced by the haplotype network analysis and neutrality test with mtDNA D-loop sequences and demographic data of microsatellite genotypes. This result may be explained by the constant selection of genes associated with economically valuable traits as a part of farm managements, which maintains consistent frequencies of advantageous genes in the farm populations. Alternatively, it can be inferred that few recent introductions of alien breeds within the stock have occurred.

### Influences on genetic factors that may affect the productivity in the North African catfish population in Thailand: Not inbreeding, but what?

The North African catfish in captive farms are often commercially transferred across the regions in the country [19]. Nowadays, the North African catfish holds significant aquacultural importance, particularly as a parental stock for producing hybrids with the bighead catfish in Thailand [13, 64]. Therefore, large population stocks in SBR, KSN, and NYK are expected to supplement parental stocks for hybrid production. Previous studies on genetic diversity among Thai captive North African catfish populations using microsatellite genotyping revealed a high *H*_e_ value (ranged from 0.67–0.80) [10, 19]; however, it was less sufficient for estimating their genetic diversity due to a deficiency in the number of markers (less than 11 loci) [24]. In this study, 15 microsatellite loci were used to examine the genetic diversity of captive North African catfish populations in Thailand. All the three populations (SBR, KSN, and NYK) exhibited high *H*_e_ and *AR* values, indicating a high adaptive potential [65]. The inheritable variety of breeding populations is crucial in aquaculture for adapting to changing environments and ensuring survival, growth, reproduction, and disease resistance [66]. Despite higher *H*_e_ compared to *H*_o_ in all the three populations significantly, *F*_IS_ and *r* values remained remarkably low, which suggests the existence of inbreeding. However, artificial selection to improve economically important traits is effective when heterozygous genotypes have a higher relative fitness than homozygous genotypes. Many individuals in the North African catfish population remain effective at transmitting genetic components within the captive population. This suggests a large *N*_e_ value that is expected to exceed the threshold of 45–50 individuals recommended by Tave [67] and Caballero et al. [68], which helps to avoid the long-term inbreeding depression. It was hypothesized that the influence of inbreeding on the low conception and fertility rates in North African catfish might be minimal. A potential subpopulation within all captive populations, suggested by positive *F* values and Bayesian structural analysis, could be observed. Genetic partitioning within population, which may also be due to the Wahlund effect [69], results from the potential mixed origin of the captive populations with founding individuals from historically distinct groups. These diverse origins of populations can help mitigate the loss of genetic variations. Despite frequent occurrences of deleterious traits associated with growth, survival, stress resistance, and reproductive abilities in the long-term breeding populations of the North African catfish, the captive populations display an exceptionally lower inbreeding value. Well-regulated farm management, disease control, and food quality are observed in Thai farmer communities [18, 70]. Why and how does the fitness and production in the breeding populations of the North African catfish decline in Thailand?

### Possible diverse genetic origins of the North African catfish in Thailand

Genetic diversity patterns of the captive populations reveal their past population dynamics and levels of genetic isolation. Most *F*_ST_ values between the populations indicated a minimal genetic differentiation except for that between the SBR and KSN populations, which may be attributed to the small number of individuals of the SBR population (N = 8). This result aligns with the findings obtained from PCoA, in which no clustering was observed among individuals localized from different populations. Such a lack of significant drift or the presence of isolated populations observed in this study may be caused by the extensive historical gene flow between the three populations, which was facilitated by the migration of captive populations artificially done for aquaculture. The results of AMOVA emphasized that a significant portion of the overall genetic variation exists within populations, indicating that gene flow may have extensively occurred between the three captive populations. Genetic similarity between the populations, shown by microsatellite genotype data, suggests a historical unidirectional gene flow from NYK to SBR or from NYK to KSN. The *N*_m_ values that exceeded 1.0 strongly suggest that the gene flow surpasses the genetic drift [71, 72]. Large-scale gene flow and subsequent genetic admixture between populations reduce the genetic variation between populations. In the past, the NYK population has served as the central hub for distributing North African catfish, particularly paternal stocks for production by breeding to aquaculture farms across the country. This suggests that the estimated gene flow corresponds to the prevailing direction of human-mediated population transfer. Substantial maternal gene flow from KSN to NYK was observed through the MIGRATE-N analysis of mtDNA D-loop sequences, indicating that the directional maternal genetic exchange occurred between the two captive populations. However, it cannot be ruled out that the potential underestimation of historical migration within the population may have contributed to low migration rates, potentially intensifying the divergence between independently sourced populations. The *r* value should be interpreted cautiously due to potential high values caused by chance. Bayesian structural analyses suggested a lack of genetic structuring in the three populations. Distinct gene pools were detected between the SBR-KSN populations and the NYK population at different *K*-levels. This suggests that the SBR-KSN and NYK populations were derived from distinct lineages. Chalermwong et al. [73] asserted that a species complex was identified within the North African catfish, which was characterized by substantial intraspecific sequence divergence, although it was classified under the same species using a species delimitation approach based on DNA barcodes. Moreover, ZZ/ZW sex determination system, which is likely the ancestral system for the North African catfish, is the same as the systems reported in Africa and Israel. However, studies on different populations from Israel, Hungary, and China have suggested the presence of an XX/XY system or the potential coexistence of both sex chromosome systems, indicating multiple systems of sex determination in the North African catfish [74–83]. This suggests that comprehensive genomic analyses of diverse populations derived from different regions are required to address the taxonomic issues in the North African catfish. Alternatively, the observed various gene pools between the North African catfish populations in Thailand may be attributed to the difference in genetic origins of domesticated strains of the same species. Examinations of fast- and slow-growing strains of the North African catfish in Nigeria have revealed that the difference in the growth rate depends on their geographic origins [84, 85]. Likewise, four African catfish populations from, Thailand, the Netherlands, Egypt, and Kenya, which are believed to harbor unique genetic advantages, were introduced to Indonesia for genetic improvement [63, 86].

Cross-mating between different populations can boost reproductive fitness through increased heterozygosity, which prevents the expression of deleterious recessive alleles. In Indonesia, the hybridization between the Egyptian and Dutch populations of the North African catfish yielded the remarkable results, such as the improvement of growth rate and survival rate [63]. However, crossbreeding may cause the reduction of fitness due to incompatibilities between genetically diverse populations. Outbreeding depression describes the phenomenon whereby outbred offspring exhibit reduced reproductive fitness compared to non-outbred offspring. Inter-population hybridization could lead to outbreeding depression, resulting in reduced fitness in the F_1_ or F_2_ generations. This could significantly reduce the survival rate of the North African catfish, particularly in the regions lacking or poorly enforcing strict regulations on inter-basin fish translocation in Africa [87]. The reduction in survival rate, growth rate, low conception, fertility rates, and fecundity actually occurs in the North African catfish populations in Thailand [19, 20]. Principally, outbreeding depression may be caused by various mechanisms, including ecological factors. Cross-mating between populations that adapt to distinctly different environments could produce the offspring that are less well-suited to the environments, as typically observed in the offspring of F_1_ generation. Interbreeding between different populations with high genetic diversity could disrupt the genetic complexity, resulting in the reduction of reproductive fitness. These genetic consequences are often observed in later generations beyond meiotic division that disrupts gene complexes by independent assortment and genetic recombination. The resource of the North African catfish in Thailand likely originated in the Central African Republic, although the possibility that they derived from Egypt remains feasible [88]. Intentional crossbreeding between different breeding populations in hatcheries aims to restore high genetic diversity but may result in the mixing of gene pools of genetically distinct populations, leading to the reduction of genetic specificity of each population in the North African catfish resources. The government-funded fish farming program from 1990 to 2012 in Thailand achieved a significant increase in the number of catfish hatcheries for the purpose of enhancing food security and increasing income in the community. However, uncontrolled crossbreeding puts the catfish industry at risk. This issue may arise mainly from the absence of planned breeding and establishment of rearing conditions (for larvae), resulting in significantly lower productivity. We hypothesize that the mixed genetic lineage contributes to the reduction in productivity of the North African catfish in Thailand. Introduction of new individuals with different genetic origins into the hatchery may significantly alter the genetic composition of the original populations, potentially leading to outbreeding depression [89]. This should be taken into account when formulating breeding plans for improving the genetic resources of the North African catfish by establishing a baseline population with distinct gene pools and mixing them.

### Significance of the North African catfish and their breeding strategies

Genetic divergence in fish populations captured from nature is far superior to domesticated populations, which avoids potential inbreeding that causes the decrease in genetic variation. However, the adaptability of the North African catfish populations may be adversely affected by outbreeding between genetically distinct populations. Selective breeding alone cannot substantially improve the productivity by avoiding outbreeding depression. It is probably challenging to identify the genetic origins of the current North African catfish populations in Thailand and to accomplish their breeding under well-developed programs laid out based on the genetic data. North African catfish populations introduced from Africa, which are characterized by high genetic diversity and purity, can be utilized as a high quality of resource to enhance the hatchery production, increase income of farmers, and improve food availability per person.

## Conclusions

Maintaining a sufficient amount of genetic variation is crucial for breeding populations of economically important species such as the North African catfish. Over the past decade, the reduction in conception rates, fertility, and production has been reported in the captive North African catfish populations in Thailand. Evaluation of the genetic diversity of the captive populations is essential for determining how to manage the genetic stock effectively. This study revealed a potentially low inbreeding value in the captive populations of the North African catfish in Thailand but also the presence of diverse genetic origins and gene pools. The mixing of breeding populations may have occurred by introducing different the North African catfish populations with different origins to Thailand, and intentional crossbreeding was probably carried out aimed at maintaining their genetic diversity. This process may have mixed the gene pools of genetically different populations, causing a loss of the genetic distinctness of the captive North African catfish resources in Thailand. This could increase the risk of outbreeding depression. Therefore, introduction of new populations with known origins into the hatcheries is required for ensuring the genetic purity of breeding populations and reducing the risk of outbreeding depression. The genetically diverse populations with known origins would serve as an ideal founder stock for selective breeding programs aimed at improving the productivity of the North African catfish.

## Supporting information

S1 Table List of 136 specimen of the North African catfish (*Clarias gariepinus*) used in this study. All mitochondrial DNA D-loop sequences were deposited in the DNA Data Bank of Japan (DDBJ), and sequence similarity search was performed with BLASTn (http://blast.ncbi.nlm.nih.gov/Blast.cgi).
(DOCX)

S2 Table Nucleotide sequences of microsatellite primers and fluorescence dyes for labeling used in this study. (DOCX)

S3 Table Pairwise comparison of linkage disequilibrium of 15 microsatellite loci in the North African catfish (*Clarias gariepinus*) from the Sing Buri population. Numbers indicate *p*-values with 110 permutations.
(DOCX)

S4 Table Pairwise comparison of linkage disequilibrium of 15 microsatellite loci in the North African catfish (*Clarias gariepinus*) from the Kalasin population. Numbers indicate *p*-values with 110 permutations.
(DOCX)

S5 Table Pairwise comparison of linkage disequilibrium of 15 microsatellite loci in the North African catfish (*Clarias gariepinus*) from the Nakhon Nayok population. Numbers indicate *p*-values with 110 permutations.
(DOCX)

S6 Table Genetic diversity of each microsatellite locus in three populations of the North African catfish (*Clarias gariepinus*). Detailed information of all individuals is presented in S1 Table.
(DOCX)

S7 Table Inbreeding coefficients (FIS) of eight individuals from the Sing Buri population. (DOCX)

S8 Table Inbreeding coefficients (*F*_IS_) of 97 individuals from the Kalasin population. (DOCX)

S9 Table Inbreeding coefficients (*F*_IS_) of 31 individuals from the Nakhon Nayok population. (DOCX)

S10 Table Distributions of genetic relatedness values (*r*) and inbreeding coefficients (*F*_IS_) for the North African catfish (*Clarias gariepinus*). (DOCX)

S11 Table Pairwise comparison of genetic relatedness (*r*) values for eight individuals from the Sing Buri population. (DOCX)

S12 Table Pairwise comparison of genetic relatedness (*r*) values for 97 individuals from the Kalasin population. (DOCX)

S13 Table Pairwise comparison of genetic relatedness (*r*) values for 31 individuals from the Nakhon Nayok population. (DOCX)

S14 Table Pairwise comparison of genetic differentiation (*F*_ST_), *F*STENA values with ENA correction for null alleles, and *R*_ST_ values between three populations. The numbers indicate *p*-values with 110 permutations.
(DOCX)

S15 Table Analysis of molecular variance (AMOVA) results for the North African catfish (*Clarias gariepinus*) based on 15 microsatellite loci. (DOCX)

S16 Table Nei’s genetic distance (*D*) values between three populations of the North African catfish. (DOCX)

S17 Table Wilcoxon sign rank test to evaluate mutation–drift equilibrium in 136 North African catfish (*Clarias gariepinus*) under different models. (DOCX)

S18 Table The mean migration rates between populations and 95% confidence intervals for standard deviation determined via BayesAss using microsatellite data for the North African catfish (*Clarias gariepinus*). (DOCX)

S19 Table Mutation-scaled effective population sizes (Θ) in three populations of the North African catfish and asymmetric migration rates (*M*) between populations among 136 North African catfish (*Clarias gariepinus*) for the estimated with 15 microsatellite loci. (DOCX)

S20 Table The effective number of immigrants (*N*_m_) from population i into population j per generation in three populations of the North African catfish (*Clarias gariepinus*) estimated with 15 microsatellite loci. (DOCX)

S21 Table Mutation-scaled effective population sizes (Θ) in three populations of the North African catfish (*Clarias gariepinus*) and asymmetric migration rates (*M*) between populations estimated with the mitochondrial DNA D-loop sequences. (DOCX)

S22 Table The effective number of immigrants (*N*_m_) from population i into population j per generation in three populations of the North African catfish (*Clarias gariepinus*) estimated by the mitochondrial DNA D-loop sequences. (DOCX)

S1 Fig Observed distribution of pairwise relatedness values and inbreeding coefficients that are plotted against the expected distributions for the North African catfish (*Clarias gariepinus*) in three populations. (A) pairwise relatedness values (*r*). (B) inbreeding coefficients (*F*_IS_).
(TIF)

S2 Fig Population structure of 136 the North African catfish. Plot of (A) Evanno’s Δ*K* and (B) ln P(*K*).
(TIF)

S3 Fig Haplotype network of three populations of North African catfish based on the data of mitochondrial DNA D-loop sequences. SBR, Sing Buri population,KSN, Kalasin population,NYK, Nakorn Nayok population.
(TIF)

S4 Fig Phylogenetic tree of the haplotypes of mitochondrial DNA D-loop sequences in three populations of the North African catfish. The two most common haplotypes (CGA4 and CGA13) were shared among three populations.
(TIF)

S5 Fig Mismatch distribution of the mitochondrial DNA D-loop sequences in three populations of the North African catfish. (A) the entire population, (B) the Sing Buri population, (C) the Kalasin population, (D) the Nakorn Nayok population. The *x*-axis represents the number of pairwise differences (mismatches), and the *y*-axis represents the frequency of these differences. The distribution of frequencies of observed mismatches (red line) is compared to those of frequencies of expected mismatches (green line).
(TIF)

S6 Fig The historical demographic fluctuations of the mitochondrial DNA D‐loop sequences of the North African catfish by coalescent Bayesian skyline analysis. The median effective population size is delimited by the black lines. The blue shaded area delimits the upper and lower bounds of the 95% highest posterior density interval. The *x*-axis represents time in years and the *y*-axis is displayed in logarithmic scale.
(TIF)
