# Supplementary material for: Questioning inbreeding: Could outbreeding affect productivity in the North African catfish in Thailand?
Source: PLoS One. 2024 May 6;19(5):e0302584. doi: 10.1371/journal.pone.0302584 (PMC11073742; doi:10.1371/journal.pone.0302584)
Supplement: S1 Table — All mitochondrial DNA D-loop sequences were deposited in the DNA Data Bank of Japan (DDBJ), and sequence similarity search was performed with BLASTn (http://blast.ncbi.nlm.nih.gov/Blast.cgi). (DOCX) [file pone.0302584.s001.docx]

**S1 Table.** List of 136 specimens of the North African catfish (*Clarias gariepinus*) used in this study. All mitochondrial DNA D-loop sequences were deposited in the DNA Data Bank of Japan (DDBJ), and sequence similarity search was performed with BLASTn (http://blast.ncbi.nlm.nih.gov/Blast.cgi).

| **No.** | **Code name** | **Species** | **Locality*** | **Accession number of mtDNA D-loop sequences in DDBJ** | **Identity (%)** |
| --- | --- | --- | --- | --- | --- |
| 1 | S1M1 | *Clarias gariepinus* | SBR, Sing Buri | LC781488 | 97.57 |
| 2 | S2M2 | *Clarias gariepinus* | SBR, Sing Buri | LC781489 | 100.00 |
| 3 | S3M3 | *Clarias gariepinus* | SBR, Sing Buri | LC781490 | 100.00 |
| 4 | S4M4 | *Clarias gariepinus* | SBR, Sing Buri | LC781404 | 98.00 |
| 5 | S5M5 | *Clarias gariepinus* | SBR, Sing Buri | LC781492 | 100.00 |
| 6 | S6M6 | *Clarias gariepinus* | SBR, Sing Buri | LC781438 | 100.00 |
| 7 | S7M7 | *Clarias gariepinus* | SBR, Sing Buri | LC781494 | 100.00 |
| 8 | S8M8 | *Clarias gariepinus* | SBR, Sing Buri | LC781360 | 95.84 |
| 9 | B1 | *Clarias gariepinus* | KSN, Kalasin | LC781467 | 100.00 |
| 10 | B2 | *Clarias gariepinus* | KSN, Kalasin | LC781459 | 98.00 |
| 11 | B3 | *Clarias gariepinus* | KSN, Kalasin | LC781458 | 98.00 |
| 12 | B4 | *Clarias gariepinus* | KSN, Kalasin | LC781415 | 98.00 |
| 13 | B5 | *Clarias gariepinus* | KSN, Kalasin | LC781417 | 98.00 |
| 14 | B6 | *Clarias gariepinus* | KSN, Kalasin | LC781419 | 100.00 |
| 15 | B7 | *Clarias gariepinus* | KSN, Kalasin | LC781421 | 100.00 |
| 16 | B8F | *Clarias gariepinus* | KSN, Kalasin | LC781457 | 100.00 |
| 17 | B9F | *Clarias gariepinus* | KSN, Kalasin | LC781425 | 100.00 |
| 18 | B10F | *Clarias gariepinus* | KSN, Kalasin | LC781361 | 100.00 |
| 19 | B11F | *Clarias gariepinus* | KSN, Kalasin | LC781363 | 99.55 |
| 20 | B12F | *Clarias gariepinus* | KSN, Kalasin | LC781365 | 98.00 |
| 21 | B13M | *Clarias gariepinus* | KSN, Kalasin | LC781367 | 100.00 |
| 22 | B14F | *Clarias gariepinus* | KSN, Kalasin | LC781369 | 100.00 |
| 23 | B15M | *Clarias gariepinus* | KSN, Kalasin | LC781372 | 99.55 |
| 24 | B16F | *Clarias gariepinus* | KSN, Kalasin | LC781373 | 98.00 |
| 25 | B17F | *Clarias gariepinus* | KSN, Kalasin | LC781466 | 98.00 |
| 26 | B18M | *Clarias gariepinus* | KSN, Kalasin | LC781376 | 99.55 |
| 27 | B19M | *Clarias gariepinus* | KSN, Kalasin | LC781380 | 100.00 |
| 28 | B20F | *Clarias gariepinus* | KSN, Kalasin | LC781383 | 100.00 |
| 29 | B21F | *Clarias gariepinus* | KSN, Kalasin | LC781385 | 98.00 |
| 30 | B22F | *Clarias gariepinus* | KSN, Kalasin | LC781387 | 100.00 |
| 31 | B23F | *Clarias gariepinus* | KSN, Kalasin | LC781389 | 100.00 |
| 32 | B24F | *Clarias gariepinus* | KSN, Kalasin | LC781391 | 98.00 |
| 33 | B25F | *Clarias gariepinus* | KSN, Kalasin | LC781393 | 98.00 |
| 34 | B26F | *Clarias gariepinus* | KSN, Kalasin | LC781395 | 98.00 |
| 35 | B27F | *Clarias gariepinus* | KSN, Kalasin | LC781397 | 98.00 |
| 36 | B28M | *Clarias gariepinus* | KSN, Kalasin | LC781400 | 98.00 |
| 37 | B29F | *Clarias gariepinus* | KSN, Kalasin | LC781401 | 98.22 |
| 38 | B30F | *Clarias gariepinus* | KSN, Kalasin | LC781382 | 98.00 |
| 39 | B31M | *Clarias gariepinus* | KSN, Kalasin | LC781478 | 100.00 |
| 40 | B32M | *Clarias gariepinus* | KSN, Kalasin | LC781406 | 100.00 |
| 41 | B33M | *Clarias gariepinus* | KSN, Kalasin | LC781409 | 98.00 |
| 42 | B34M | *Clarias gariepinus* | KSN, Kalasin | LC781410 | 98.00 |
| 43 | B35M | *Clarias gariepinus* | KSN, Kalasin | LC781411 | 98.00 |
| 44 | B36M | *Clarias gariepinus* | KSN, Kalasin | LC781412 | 100.00 |
| 45 | B37M | *Clarias gariepinus* | KSN, Kalasin | LC781413 | 98.00 |
| 46 | C1M | *Clarias gariepinus* | KSN, Kalasin | LC781382 | 100.00 |
| 47 | C2M | *Clarias gariepinus* | KSN, Kalasin | LC781493 | 98.00 |
| 48 | C3M | *Clarias gariepinus* | KSN, Kalasin | LC781379 | 100.00 |
| 49 | C4M | *Clarias gariepinus* | KSN, Kalasin | LC781491 | 98.00 |
| 50 | C5M | *Clarias gariepinus* | KSN, Kalasin | LC781390 | 100.00 |
| 51 | C6M | *Clarias gariepinus* | KSN, Kalasin | LC781378 | 100.00 |
| 52 | C7M | *Clarias gariepinus* | KSN, Kalasin | LC781377 | 100.00 |
| 53 | C8M | *Clarias gariepinus* | KSN, Kalasin | LC781404 | 100.00 |
| 54 | C9M | *Clarias gariepinus* | KSN, Kalasin | LC781438 | 97.78 |
| 55 | C10M | *Clarias gariepinus* | KSN, Kalasin | LC781467 | 98.00 |
| 56 | C11M | *Clarias gariepinus* | KSN, Kalasin | LC781405 | 100.00 |
| 57 | C12M | *Clarias gariepinus* | KSN, Kalasin | LC781466 | 98.00 |
| 58 | C13M | *Clarias gariepinus* | KSN, Kalasin | LC781435 | 98.67 |
| 59 | C14M | *Clarias gariepinus* | KSN, Kalasin | LC781376 | 100.00 |
| 60 | C15M | *Clarias gariepinus* | KSN, Kalasin | LC781464 | 98.00 |
| 61 | C16M | *Clarias gariepinus* | KSN, Kalasin | LC781462 | 98.00 |
| 62 | C17M | *Clarias gariepinus* | KSN, Kalasin | LC781459 | 98.00 |
| 63 | C18M | *Clarias gariepinus* | KSN, Kalasin | LC781423 | 99.55 |
| 64 | C19M | *Clarias gariepinus* | KSN, Kalasin | LC781458 | 98.00 |
| 65 | C20M | *Clarias gariepinus* | KSN, Kalasin | LC781362 | 95.99 |
| 66 | C21M | *Clarias gariepinus* | KSN, Kalasin | LC781478 | 98.00 |
| 67 | C22M | *Clarias gariepinus* | KSN, Kalasin | LC781406 | 100.00 |
| 68 | C23M | *Clarias gariepinus* | KSN, Kalasin | LC781457 | 98.00 |
| 69 | C24M | *Clarias gariepinus* | KSN, Kalasin | LC781375 | 100.00 |
| 70 | C25M | *Clarias gariepinus* | KSN, Kalasin | LC781407 | 100.00 |
| 71 | C26M | *Clarias gariepinus* | KSN, Kalasin | LC781456 | 98.00 |
| 72 | C27M | *Clarias gariepinus* | KSN, Kalasin | LC781495 | 98.00 |
| 73 | C28M | *Clarias gariepinus* | KSN, Kalasin | LC781408 | 100.00 |
| 74 | C29M | *Clarias gariepinus* | KSN, Kalasin | LC781455 | 98.00 |
| 75 | C30M | *Clarias gariepinus* | KSN, Kalasin | LC781454 | 98.00 |
| 76 | B1m | *Clarias gariepinus* | KSN, Kalasin | LC781381 | 98.00 |
| 77 | B2m | *Clarias gariepinus* | KSN, Kalasin | LC781403 | 100.00 |
| 78 | B3m | *Clarias gariepinus* | KSN, Kalasin | LC781414 | 100.00 |
| 79 | B4m | *Clarias gariepinus* | KSN, Kalasin | LC781416 | 100.00 |
| 80 | B5m | *Clarias gariepinus* | KSN, Kalasin | LC781418 | 98.00 |
| 81 | B6m | *Clarias gariepinus* | KSN, Kalasin | LC781420 | 99.55 |
| 82 | B7m | *Clarias gariepinus* | KSN, Kalasin | LC781422 | 99.78 |
| 83 | B8m | *Clarias gariepinus* | KSN, Kalasin | LC781424 | 98.00 |
| 84 | B9m | *Clarias gariepinus* | KSN, Kalasin | LC781426 | 99.55 |
| 85 | B10m | *Clarias gariepinus* | KSN, Kalasin | LC781405 | 98.00 |
| 86 | B11m | *Clarias gariepinus* | KSN, Kalasin | LC781364 | 100.00 |
| 87 | B12m | *Clarias gariepinus* | KSN, Kalasin | LC781366 | 98.00 |
| 88 | B13m | *Clarias gariepinus* | KSN, Kalasin | LC781368 | 100.00 |
| 89 | B14m | *Clarias gariepinus* | KSN, Kalasin | LC781370 | 100.00 |
| 90 | B15m | *Clarias gariepinus* | KSN, Kalasin | LC781371 | 100.00 |
| 91 | B16m | *Clarias gariepinus* | KSN, Kalasin | LC781374 | 98.00 |
| 92 | B17m | *Clarias gariepinus* | KSN, Kalasin | LC781435 | 100.00 |
| 93 | B18m | *Clarias gariepinus* | KSN, Kalasin | LC781464 | 98.00 |
| 94 | B19m | *Clarias gariepinus* | KSN, Kalasin | LC781462 | 100.00 |
| 95 | B20m | *Clarias gariepinus* | KSN, Kalasin | LC781384 | 100.00 |
| 96 | B21m | *Clarias gariepinus* | KSN, Kalasin | LC781386 | 99.55 |
| 97 | B22m | *Clarias gariepinus* | KSN, Kalasin | LC781388 | 98.00 |
| 98 | B23m | *Clarias gariepinus* | KSN, Kalasin | LC781423 | 98.00 |
| 99 | B24m | *Clarias gariepinus* | KSN, Kalasin | LC781392 | 98.44 |
| 100 | B25m | *Clarias gariepinus* | KSN, Kalasin | LC781394 | 98.44 |
| 101 | B26m | *Clarias gariepinus* | KSN, Kalasin | LC781396 | 100.00 |
| 102 | B27m | *Clarias gariepinus* | KSN, Kalasin | LC781398 | 98.00 |
| 103 | B28m | *Clarias gariepinus* | KSN, Kalasin | LC781399 | 100.00 |
| 104 | B29m | *Clarias gariepinus* | KSN, Kalasin | LC781402 | 94.42 |
| 105 | B30m | *Clarias gariepinus* | KSN, Kalasin | LC781362 | 100.00 |
| 106 | N1F | *Clarias gariepinus* | NYK, Nakhon Nayok | LC781418 | 98.00 |
| 107 | N2M | *Clarias gariepinus* | NYK, Nakhon Nayok | LC781447 | 98.00 |
| 108 | N3F | *Clarias gariepinus* | NYK, Nakhon Nayok | LC781463 | 85.71 |
| 109 | N4M | *Clarias gariepinus* | NYK, Nakhon Nayok | LC781465 | 98.00 |
| 110 | N5M | *Clarias gariepinus* | NYK, Nakhon Nayok | LC781471 | 86.50 |
| 111 | N6M | *Clarias gariepinus* | NYK, Nakhon Nayok | LC781472 | 98.00 |
| 112 | N7F | *Clarias gariepinus* | NYK, Nakhon Nayok | LC781473 | 97.78 |
| 113 | N8F | *Clarias gariepinus* | NYK, Nakhon Nayok | LC781480 | 98.00 |
| 114 | N9F | *Clarias gariepinus* | NYK, Nakhon Nayok | LC781494 | 99.33 |
| 115 | N10F | *Clarias gariepinus* | NYK, Nakhon Nayok | LC781360 | 98.00 |
| 116 | N11M | *Clarias gariepinus* | NYK, Nakhon Nayok | LC781361 | 99.33 |
| 117 | N12M | *Clarias gariepinus* | NYK, Nakhon Nayok | LC781365 | 98.00 |
| 118 | N13F | *Clarias gariepinus* | NYK, Nakhon Nayok | LC781367 | 98.67 |
| 119 | N15F | *Clarias gariepinus* | NYK, Nakhon Nayok | LC781369 | 98.00 |
| 120 | N16M | *Clarias gariepinus* | NYK, Nakhon Nayok | LC781370 | 98.00 |
| 121 | N17F | *Clarias gariepinus* | NYK, Nakhon Nayok | LC781371 | 98.00 |
| 122 | N18F | *Clarias gariepinus* | NYK, Nakhon Nayok | LC781395 | 98.00 |
| 123 | N19F | *Clarias gariepinus* | NYK, Nakhon Nayok | LC781412 | 99.55 |
| 124 | N20F | *Clarias gariepinus* | NYK, Nakhon Nayok | LC781419 | 100.00 |
| 125 | N21F | *Clarias gariepinus* | NYK, Nakhon Nayok | LC781420 | 100.00 |
| 126 | N22F | *Clarias gariepinus* | NYK, Nakhon Nayok | LC781421 | 99.55 |
| 127 | N23F | *Clarias gariepinus* | NYK, Nakhon Nayok | LC781428 | 98.00 |
| 128 | N24F | *Clarias gariepinus* | NYK, Nakhon Nayok | LC781433 | 98.00 |
| 129 | N25M | *Clarias gariepinus* | NYK, Nakhon Nayok | LC781434 | 99.55 |
| 130 | N26M | *Clarias gariepinus* | NYK, Nakhon Nayok | LC781439 | 98.00 |
| 131 | N27M | *Clarias gariepinus* | NYK, Nakhon Nayok | LC781441 | 98.00 |
| 132 | N28M | *Clarias gariepinus* | NYK, Nakhon Nayok | LC781445 | 100.00 |
| 133 | N29M | *Clarias gariepinus* | NYK, Nakhon Nayok | LC781446 | 100.00 |
| 134 | N30M | *Clarias gariepinus* | NYK, Nakhon Nayok | LC781448 | 100.00 |
| 135 | N31M | *Clarias gariepinus* | NYK, Nakhon Nayok | LC781450 | 97.78 |
| 136 | N32M | *Clarias gariepinus* | NYK, Nakhon Nayok | LC781461 | 100.00 |

*SBR, Sing Buri; KSN, Kalasin; NYK, Nakhon Nayok.
