# Supplementary material for: Questioning inbreeding: Could outbreeding affect productivity in the North African catfish in Thailand?
Source: PLoS One. 2024 May 6;19(5):e0302584. doi: 10.1371/journal.pone.0302584 (PMC11073742; doi:10.1371/journal.pone.0302584)
Supplement: S2 Table — (DOCX) [file pone.0302584.s002.docx]

**S2 Table.** Nucleotide sequences of microsatellite primers and fluorescence dyes for labeling used in this study.

| **Primer** | **Fluorescence**  **dye** | **Primer sequence 5′ to 3′** | | **Size (bp)** | **Repeat motifs** |
| --- | --- | --- | --- | --- | --- |
|  |  | **Forward** | **Reward** |  |  |
| **Cg002** | HEX | AGCAGGAAAACGGGTCAC | ATGGTTCAGCTGTAGTGTTGG | 82 – 112 | ($\text{CA)}_{26}$ |
| **Cg003** | HEX | CGCCCTACCTGTAACCTGT | CGAGTTTCCAGGTAGAGCAG | 132 – 150 | ($\text{CA)}_{17}$ |
| **Cg010** | HEX | GTCATGCTGGGAGAACAAGA | TGCATGATAGGTGAAAGGGT | 92 – 136 | ($\text{GT)}_{13}$ |
| **Cg175** | TAMRA | CCTGAAGAAGAAATCTCAGATAAGT | ATCAGACTGAGGATGAGGTTCA | 153 – 200 | ($\text{AC)}_{15}$ |
| **Cg214** | FAM | CCTGCATTATTCCCTTTGAC | AATAACAGACTATGTTGAATTAATGG | 139 – 177 | ($\text{CA)}_{15}$ |
| **Cg294** | HEX | CAGCTGTCGATGTATGACCTG | GACCTGAAGGAGCCTGTCTG | 307 – 338 | ($\text{TG)}_{18}$ |
| **Cg312** | FAM | TTGTGCACTGCAAGCGAT | AGTCAATGCATTTGGACAGC | 196 – 227 | ($\text{CA)}_{14}$ |
| **Cg316** | FAM | GCTCGATTTCTGATTTACAGG | CGGAGAAGTGCCGTAGCT | 78 – 112 | ($\text{AG)}_{18}$ |
| **Cg339** | TAMRA | GCTCGATTTCTGATTTACAGG | CCCCTTACAGCTTCATTTTCC | 134 – 196 | ($\text{TGTGTGAGAG)}_{8}$ |
| **Cg352** | TAMRA | TGGTTGAGATCGAGGTTGG | TGACTCAGACTCGGAGACCTAC | 204 – 246 | ($\text{GT)}_{19}$ |
| **Cg639** | TAMRA | CAGCTTTGGCTCGGTCA | AACACGTTCAAGCGGTAGTC | 177 – 220 | ($\text{AC)}_{10}$ |
| **Cg647** | TAMRA | GGTCAGCACCACCTGAGAA | TCCAGCTTTTAGCAAGTGTTAG | 116 – 132 | ($\text{GT)}_{10}$ |
| **Cg661** | FAM | TTGGGTTTATCCGTGGTTC | GAGATGCTGGTGATGGTGAG | 106 – 147 | ($\text{AC)}_{13}$ |
| **Cga01** | FAM | GGCTAAAAGAACCCTGTCTG | TACAGCGTCGATAAGCCAGG | 107 – 212 | ($\text{GT)}_{15}$ |
| **Cga03** | FAM | CACTTCTTACATTTGTGCCC | ACCTGTATTGATTTCTTGCC | 94 – 278 | ($\text{GT)}_{21}$ |
