# Supplementary material for: Questioning inbreeding: Could outbreeding affect productivity in the North African catfish in Thailand?
Source: PLoS One. 2024 May 6;19(5):e0302584. doi: 10.1371/journal.pone.0302584 (PMC11073742; doi:10.1371/journal.pone.0302584)
Supplement: S3 Table — Numbers indicate p-values with 110 permutations. (DOCX) [file pone.0302584.s003.docx]

**S3 Table.** Pairwise comparison of linkage disequilibrium of 15 microsatellite loci in the North African catfish (*Clarias gariepinus*) from the Sing Buri population. Numbers indicate *p*-values with 110 permutations.

| **Locus** | **Cg002** | **Cg003** | **Cg010** | **Cg175** | **Cg214** | **Cg294** | **Cg312** | **Cg316** | **Cg339** | **Cg352** | **Cg639** | **Cg647** | **Cg661** | **Cga01** | **Cga03** |
| --- | --- | --- | --- | --- | --- | --- | --- | --- | --- | --- | --- | --- | --- | --- | --- |
| **Cg002** |  |  |  |  |  |  |  |  |  |  |  |  |  |  |  |
| **Cg003** | 0.503 |  |  |  |  |  |  |  |  |  |  |  |  |  |  |
| **Cg010** | N/A | 1.000 |  |  |  |  |  |  |  |  |  |  |  |  |  |
| **Cg175** | 1.000 | 0.804 | 1.000 |  |  |  |  |  |  |  |  |  |  |  |  |
| **Cg214** | 1.000 | 0.139 | 1.000 | 0.486 |  |  |  |  |  |  |  |  |  |  |  |
| **Cg294** | 1.000 | 1.000 | 1.000 | 1.000 | 1.000 |  |  |  |  |  |  |  |  |  |  |
| **Cg312** | 1.000 | 1.000 | 1.000 | 1.000 | 1.000 | 1.000 |  |  |  |  |  |  |  |  |  |
| **Cg316** | N/A | 0.807 | 1.000 | 1.000 | 1.000 | 1.000 | 1.000 |  |  |  |  |  |  |  |  |
| **Cg339** | 1.000 | 0.729 | 0.219 | 0.683 | 0.656 | 1.000 | 0.569 | 0.409 |  |  |  |  |  |  |  |
| **Cg352** | 1.000 | 0.154 | 1.000 | 0.508 | 0.487 | 1.000 | 0.396 | 0.282 | 0.690 |  |  |  |  |  |  |
| **Cg639** | 1.000 | 1.000 | 1.000 | 1.000 | 1.000 | 1.000 | 0.314 | 1.000 | 1.000 | 1.000 |  |  |  |  |  |
| **Cg647** | N/A | N/A | N/A | N/A | N/A | N/A | N/A | N/A | N/A | N/A | N/A |  |  |  |  |
| **Cg661** | 1.000 | 1.000 | 0.222 | 1.000 | 0.176 | 1.000 | 1.000 | 0.451 | 0.243 | 1.000 | 1.000 | N/A |  |  |  |
| **Cga01** | 1.000 | 1.000 | 1.000 | 1.000 | 0.295 | 1.000 | 1.000 | 1.000 | 1.000 | 1.000 | 1.000 | N/A | 0.361 |  |  |
| **Cga03** | 1.000 | 0.724 | 1.000 | 0.898 | 0.174 | 1.000 | 0.214 | 0.654 | 0.499 | 0.163 | 1.000 | N/A | 0.716 | 0.533 |  |
