# Supplementary material for: Questioning inbreeding: Could outbreeding affect productivity in the North African catfish in Thailand?
Source: PLoS One. 2024 May 6;19(5):e0302584. doi: 10.1371/journal.pone.0302584 (PMC11073742; doi:10.1371/journal.pone.0302584)
Supplement: S4 Table — Numbers indicate p-values with 110 permutations. (DOCX) [file pone.0302584.s004.docx]

**S4 Table.** Pairwise comparison of linkage disequilibrium of 15 microsatellite loci in the North African catfish (*Clarias gariepinus*) from the Kalasin population. Numbers indicate *p*-values with 110 permutations.

| **Locus** | **Cg002** | **Cg003** | **Cg010** | **Cg175** | **Cg214** | **Cg294** | **Cg312** | **Cg316** | **Cg339** | **Cg352** | **Cg639** | **Cg647** | **Cg661** | **Cga01** | **Cga03** |
| --- | --- | --- | --- | --- | --- | --- | --- | --- | --- | --- | --- | --- | --- | --- | --- |
| **Cg002** |  |  |  |  |  |  |  |  |  |  |  |  |  |  |  |
| **Cg003** | 0.660 |  |  |  |  |  |  |  |  |  |  |  |  |  |  |
| **Cg010** | 0.029 | 0.882 |  |  |  |  |  |  |  |  |  |  |  |  |  |
| **Cg175** | 0.503 | 0.023 | 0.525 |  |  |  |  |  |  |  |  |  |  |  |  |
| **Cg214** | 0.051 | 0.093 | 0.084 | 0.000 |  |  |  |  |  |  |  |  |  |  |  |
| **Cg294** | 0.087 | 0.084 | 0.014 | 0.129 | 0.170 |  |  |  |  |  |  |  |  |  |  |
| **Cg312** | 0.105 | 0.596 | 0.789 | 0.019 | 0.575 | 0.028 |  |  |  |  |  |  |  |  |  |
| **Cg316** | 0.151 | 0.591 | 0.239 | 0.260 | 0.397 | 0.644 | 0.019 |  |  |  |  |  |  |  |  |
| **Cg339** | 0.005 | 0.977 | 0.414 | 0.057 | 0.899 | 0.193 | 0.503 | 0.639 |  |  |  |  |  |  |  |
| **Cg352** | 0.000 | 0.751 | 0.733 | 0.146 | 0.594 | 0.079 | 0.318 | 0.676 | 0.178 |  |  |  |  |  |  |
| **Cg639** | 0.268 | 0.571 | 0.516 | 0.153 | 0.289 | 0.539 | 0.103 | 0.100 | 0.528 | 0.106 |  |  |  |  |  |
| **Cg647** | 0.509 | 0.529 | 0.214 | 0.382 | 0.129 | 0.953 | 0.418 | 0.097 | 0.543 | 0.043 | 0.269 |  |  |  |  |
| **Cg661** | 0.265 | 0.188 | 0.830 | 0.025 | 0.910 | 0.332 | 0.011 | 0.317 | 0.783 | 0.125 | 0.276 | 0.213 |  |  |  |
| **Cga01** | 0.006 | 0.950 | 0.187 | 0.299 | 0.499 | 0.594 | 0.450 | 0.164 | 0.213 | 0.598 | 0.689 | 0.001 | 0.908 |  |  |
| **Cga03** | 0.663 | 0.962 | 0.143 | 0.615 | 0.645 | 0.419 | 0.115 | 0.239 | 0.137 | 0.088 | 0.916 | 0.251 | 0.002 | 0.949 |  |
