# Supplementary material for: Questioning inbreeding: Could outbreeding affect productivity in the North African catfish in Thailand?
Source: PLoS One. 2024 May 6;19(5):e0302584. doi: 10.1371/journal.pone.0302584 (PMC11073742; doi:10.1371/journal.pone.0302584)
Supplement: S5 Table — Numbers indicate p-values with 110 permutations. (DOCX) [file pone.0302584.s005.docx]

**S5 Table.** Pairwise comparison of linkage disequilibrium of 15 microsatellite loci in the North African catfish (*Clarias gariepinus*) from the Nakhon Nayok population. Numbers indicate *p*-values with 110 permutations.

| **Locus** | **Cg002** | **Cg003** | **Cg010** | **Cg175** | **Cg214** | **Cg294** | **Cg312** | **Cg316** | **Cg339** | **Cg352** | **Cg639** | **Cg647** | **Cg661** | **Cga01** | **Cga03** |
| --- | --- | --- | --- | --- | --- | --- | --- | --- | --- | --- | --- | --- | --- | --- | --- |
| **Cg002** |  |  |  |  |  |  |  |  |  |  |  |  |  |  |  |
| **Cg003** | 0.386 |  |  |  |  |  |  |  |  |  |  |  |  |  |  |
| **Cg010** | 0.482 | 0.637 |  |  |  |  |  |  |  |  |  |  |  |  |  |
| **Cg175** | 1.000 | 0.335 | 1.000 |  |  |  |  |  |  |  |  |  |  |  |  |
| **Cg214** | 0.220 | 0.912 | 0.618 | 1.000 |  |  |  |  |  |  |  |  |  |  |  |
| **Cg294** | 0.376 | 0.670 | 0.819 | 0.710 | 0.304 |  |  |  |  |  |  |  |  |  |  |
| **Cg312** | 0.936 | 0.925 | 0.162 | 0.776 | 0.860 | 0.120 |  |  |  |  |  |  |  |  |  |
| **Cg316** | 0.027 | 0.713 | 0.929 | 1.000 | 0.329 | 0.986 | 0.515 |  |  |  |  |  |  |  |  |
| **Cg339** | 0.135 | 0.385 | 0.412 | 1.000 | 0.792 | 0.326 | 0.058 | 0.671 |  |  |  |  |  |  |  |
| **Cg352** | 0.806 | 0.944 | 0.423 | 1.000 | 1.000 | 0.798 | 0.128 | 1.000 | 0.748 |  |  |  |  |  |  |
| **Cg639** | 0.679 | 0.541 | 0.025 | 0.212 | 0.318 | 0.389 | 0.506 | 0.947 | 0.135 | 0.783 |  |  |  |  |  |
| **Cg647** | 0.932 | 0.165 | 0.078 | 0.480 | 0.835 | 0.261 | 0.321 | 0.931 | 0.670 | 0.072 | 0.454 |  |  |  |  |
| **Cg661** | 0.535 | 0.155 | 0.908 | 0.314 | 0.840 | 0.828 | 0.703 | 0.250 | 0.225 | 0.670 | 0.013 | 0.810 |  |  |  |
| **Cga01** | 0.231 | 0.217 | 0.184 | 0.436 | 0.816 | 0.359 | 0.703 | 0.677 | 0.240 | 0.012 | 0.000 | 0.152 | 0.713 |  |  |
| **Cga03** | 0.067 | 0.422 | 0.007 | 0.980 | 0.310 | 0.087 | 0.970 | 0.974 | 0.736 | 0.974 | 0.315 | 0.478 | 0.115 | 0.784 |  |
