# Supplementary material for: Questioning inbreeding: Could outbreeding affect productivity in the North African catfish in Thailand?
Source: PLoS One. 2024 May 6;19(5):e0302584. doi: 10.1371/journal.pone.0302584 (PMC11073742; doi:10.1371/journal.pone.0302584)
Supplement: S6 Table — Detailed information of all individuals is presented in S1 Table. (DOCX) [file pone.0302584.s006.docx]

**S6 Table.** Genetic diversity of each microsatellite locus in three populations of the North African catfish (*Clarias gariepinus*). Detailed information of all individuals is presented in Supplementary Table S1.

| **Population*** | **Locus** | **N** | ***N*_a_** | ***AR*** | ***N*_ea_** | ***I*** | ***H*_o_** | ***H*_e_** | ***M-ratio*** | ***PIC*** | ***F*** |
| --- | --- | --- | --- | --- | --- | --- | --- | --- | --- | --- | --- |
| SBR | Cg002 | 5 | 3.000 | 2.800 | 2.174 | 0.898 | 0.400 | 0.540 | 0.500 | 0.466 | 0.259 |
|  | Cg003 | 7 | 3.000 | 2.670 | 1.815 | 0.796 | 0.000 | 0.449 | 0.750 | 0.406 | 1.000 |
|  | Cg010 | 8 | 5.000 | 4.125 | 3.879 | 1.461 | 0.625 | 0.742 | 0.313 | 0.701 | 0.158 |
|  | Cg175 | 8 | 5.000 | 3.728 | 2.977 | 1.300 | 0.500 | 0.664 | 0.179 | 0.618 | 0.247 |
|  | Cg214 | 8 | 5.000 | 4.017 | 3.657 | 1.424 | 0.125 | 0.727 | 0.132 | 0.682 | 0.828 |
|  | Cg294 | 8 | 4.000 | 3.810 | 3.879 | 1.371 | 0.375 | 0.742 | 0.143 | 0.694 | 0.495 |
|  | Cg312 | 8 | 4.000 | 3.361 | 2.844 | 1.180 | 0.750 | 0.648 | 0.200 | 0.592 | -0.157 |
|  | Cg316 | 8 | 6.000 | 4.410 | 4.267 | 1.576 | 0.375 | 0.766 | 0.273 | 0.728 | 0.510 |
|  | Cg339 | 8 | 4.000 | 3.262 | 2.844 | 1.163 | 0.875 | 0.648 | 0.100 | 0.582 | -0.349 |
|  | Cg352 | 8 | 6.000 | 4.433 | 3.765 | 1.542 | 0.500 | 0.734 | 0.167 | 0.702 | 0.319 |
|  | Cg639 | 8 | 4.000 | 3.361 | 2.844 | 1.180 | 0.375 | 0.648 | 0.286 | 0.592 | 0.422 |
|  | Cg647 | 4 | 3.000 | 3.000 | 2.909 | 1.082 | 0.250 | 0.656 | 0.375 | 0.582 | 0.619 |
|  | Cg661 | 8 | 3.000 | 2.728 | 2.133 | 0.900 | 0.250 | 0.531 | 0.079 | 0.468 | 0.529 |
|  | Cga01 | 7 | 5.000 | 4.322 | 4.261 | 1.512 | 0.143 | 0.765 | 0.313 | 0.726 | 0.813 |
|  | Cga03 | 8 | 4.000 | 3.300 | 2.286 | 1.074 | 0.000 | 0.563 | 0.063 | 0.524 | 1.000 |
|  | Mean | 7.400 | 4.267 | 3.555 | 3.102 | 1.231 | 0.370 | 0.655 | 0.258 | 0.604 | 0.446 |
|  | SE | 0.321 | 0.267 | 0.159 | 0.207 | 0.065 | 0.066 | 0.025 | 0.047 | 0.027 | 0.100 |
| KSN | Cg002 | 96 | 6.000 | 5.990 | 4.344 | 1.554 | 0.469 | 0.770 | 0.375 | 0.732 | 0.391 |
|  | Cg003 | 86 | 4.000 | 4.000 | 2.631 | 1.093 | 0.070 | 0.620 | 0.667 | 0.552 | 0.887 |
|  | Cg010 | 97 | 9.000 | 8.862 | 3.827 | 1.544 | 0.526 | 0.739 | 0.080 | 0.696 | 0.288 |
|  | Cg175 | 96 | 11.000 | 11.000 | 8.325 | 2.222 | 0.750 | 0.880 | 0.367 | 0.868 | 0.148 |
|  | Cg214 | 97 | 8.000 | 8.000 | 6.281 | 1.946 | 0.567 | 0.841 | 0.211 | 0.822 | 0.326 |
|  | Cg294 | 97 | 8.000 | 7.999 | 4.539 | 1.715 | 0.351 | 0.780 | 0.267 | 0.751 | 0.550 |
|  | Cg312 | 97 | 7.000 | 6.999 | 2.821 | 1.329 | 0.258 | 0.646 | 0.438 | 0.605 | 0.601 |
|  | Cg316 | 97 | 11.000 | 10.862 | 4.566 | 1.779 | 0.680 | 0.781 | 0.423 | 0.751 | 0.129 |
|  | Cg339 | 88 | 6.000 | 6.000 | 3.580 | 1.406 | 0.341 | 0.721 | 0.100 | 0.671 | 0.527 |
|  | Cg352 | 96 | 9.000 | 8.896 | 6.083 | 1.931 | 0.542 | 0.836 | 0.346 | 0.816 | 0.352 |
|  | Cg639 | 97 | 6.000 | 5.988 | 3.712 | 1.457 | 0.629 | 0.731 | 0.429 | 0.687 | 0.139 |
|  | Cg647 | 97 | 5.000 | 4.988 | 2.836 | 1.179 | 0.392 | 0.647 | 0.500 | 0.586 | 0.395 |
|  | Cg661 | 97 | 10.000 | 9.885 | 4.909 | 1.846 | 0.691 | 0.796 | 0.227 | 0.770 | 0.133 |
|  | Cga01 | 92 | 7.000 | 7.000 | 5.177 | 1.717 | 0.217 | 0.807 | 0.292 | 0.778 | 0.731 |
|  | Cga03 | 97 | 7.000 | 6.999 | 3.723 | 1.486 | 0.196 | 0.731 | 0.103 | 0.686 | 0.732 |
|  | Mean | 95.133 | 7.600 | 7.565 | 4.490 | 1.614 | 0.445 | 0.755 | 0.321 | 0.718 | 0.422 |
|  | SE | 0.920 | 0.542 | 0.533 | 0.394 | 0.079 | 0.053 | 0.019 | 0.039 | 0.023 | 0.063 |
| NYK | Cg002 | 31 | 5.000 | 5.000 | 3.963 | 1.473 | 0.355 | 0.748 | 0.500 | 0.706 | 0.525 |
|  | Cg003 | 28 | 4.000 | 4.000 | 3.358 | 1.297 | 0.107 | 0.702 | 0.667 | 0.652 | 0.847 |
|  | Cg010 | 31 | 6.000 | 5.903 | 4.388 | 1.579 | 0.710 | 0.772 | 0.273 | 0.737 | 0.081 |
|  | Cg175 | 31 | 13.000 | 12.871 | 9.376 | 2.371 | 0.516 | 0.893 | 0.406 | 0.884 | 0.422 |
|  | Cg214 | 31 | 8.000 | 7.895 | 4.854 | 1.770 | 0.677 | 0.794 | 0.211 | 0.766 | 0.147 |
|  | Cg294 | 31 | 6.000 | 5.992 | 2.073 | 1.127 | 0.258 | 0.518 | 0.214 | 0.496 | 0.502 |
|  | Cg312 | 31 | 4.000 | 4.000 | 2.506 | 1.129 | 0.323 | 0.601 | 0.667 | 0.557 | 0.463 |
|  | Cg316 | 31 | 8.000 | 7.806 | 4.512 | 1.709 | 0.613 | 0.778 | 0.400 | 0.747 | 0.213 |
|  | Cg339 | 28 | 5.000 | 5.000 | 3.621 | 1.420 | 0.429 | 0.724 | 0.125 | 0.678 | 0.408 |
|  | Cg352 | 30 | 9.000 | 9.000 | 7.031 | 2.071 | 0.567 | 0.858 | 0.250 | 0.843 | 0.339 |
|  | Cg639 | 31 | 6.000 | 5.806 | 3.668 | 1.420 | 0.645 | 0.727 | 0.429 | 0.680 | 0.113 |
|  | Cg647 | 31 | 5.000 | 4.903 | 2.843 | 1.203 | 0.516 | 0.648 | 0.500 | 0.582 | 0.204 |
|  | Cg661 | 31 | 9.000 | 8.887 | 4.021 | 1.718 | 0.452 | 0.751 | 0.375 | 0.723 | 0.399 |
|  | Cga01 | 30 | 5.000 | 5.000 | 3.681 | 1.403 | 0.300 | 0.728 | 0.313 | 0.680 | 0.588 |
|  | Cga03 | 31 | 4.000 | 3.999 | 1.934 | 0.927 | 0.194 | 0.483 | 0.167 | 0.447 | 0.599 |
|  | Mean | 30.467 | 6.467 | 6.404 | 4.122 | 1.508 | 0.444 | 0.715 | 0.366 | 0.679 | 0.390 |
|  | SE | 0.274 | 0.646 | 0.637 | 0.494 | 0.098 | 0.048 | 0.029 | 0.040 | 0.031 | 0.055 |

N, Sample size; *N*_a_, number of alleles; *AR*, allelic richness; *N*_ea_, number of effective alleles; *I*, Shannon’s information index; *H*_o_, observed heterozygosity; *H*_e_, expected heterozygosity; *PIC*, polymorphic information content; *F*, fixation index.

*SBR, Sing Buri; KSN, Kalasin; NYK, Nakhon Nayok.
