# Supplementary material for: Questioning inbreeding: Could outbreeding affect productivity in the North African catfish in Thailand?
Source: PLoS One. 2024 May 6;19(5):e0302584. doi: 10.1371/journal.pone.0302584 (PMC11073742; doi:10.1371/journal.pone.0302584)
Supplement: S7 Table — (DOCX) [file pone.0302584.s007.docx]

**S7 Table.** Inbreeding coefficients (*F*_IS_) of eight individuals from the Sing Buri population.

| **Individual** | ***F*_IS_** |
| --- | --- |
| S1M1 | 0.127 |
| S2M2 | 0.006 |
| S3M3 | -0.064 |
| S4M4 | 0.142 |
| S5M5 | 0.227 |
| S6M6 | 0.245 |
| S7M7 | 0.079 |
| S8M8 | 0.014 |
