# Supplementary material for: Questioning inbreeding: Could outbreeding affect productivity in the North African catfish in Thailand?
Source: PLoS One. 2024 May 6;19(5):e0302584. doi: 10.1371/journal.pone.0302584 (PMC11073742; doi:10.1371/journal.pone.0302584)
Supplement: S8 Table — (DOCX) [file pone.0302584.s008.docx]

**S8 Table.** Inbreeding coefficients (*F*_IS_) of 97 individuals from the Kalasin population.

| **Individual** | ***F*_IS_** |
| --- | --- |
| B1 | 0.138 |
| B2 | 0.256 |
| B3 | 0.289 |
| B4 | 0.141 |
| B5 | -0.023 |
| B6 | 0.173 |
| B7 | -0.015 |
| B8F | 0.015 |
| B9F | -0.048 |
| B10F | 0.194 |
| B11F | -0.098 |
| B12F | 0.125 |
| B13M | 0.054 |
| B14F | 0.112 |
| B15M | 0.021 |
| B16F | -0.001 |
| B17F | 0.130 |
| B18M | -0.058 |
| B19M | -0.028 |
| B20F | -0.080 |
| B21F | 0.054 |
| B22F | 0.161 |
| B23F | 0.332 |
| B24F | 0.259 |
| B25F | 0.224 |
| B26F | -0.083 |
| B27F | 0.086 |
| B28M | -0.025 |
| B29F | 0.168 |
| B30F | 0.124 |
| B31M | 0.023 |
| B32M | 0.047 |
| B33M | -0.010 |
| B34M | 0.201 |
| B35M | 0.056 |
| B36M | 0.154 |
| B37M | -0.002 |
| C1M | -0.134 |
| C2M | 0.012 |
| C3M | 0.097 |
| C4M | 0.140 |
| C5M | -0.011 |
| C6M | 0.147 |
| C7M | -0.014 |
| C8M | 0.040 |
| C9M | 0.014 |
| C10M | -0.089 |
| C11M | -0.031 |
| C12M | 0.017 |
| C13M | 0.031 |
| C14M | 0.087 |
| C15M | -0.030 |
| C16M | -0.004 |
| C17M | 0.180 |
| C18M | 0.055 |
| C19M | -0.045 |
| C20M | -0.075 |
| C21M | 0.150 |
| C22M | -0.050 |
| C23M | -0.031 |
| C24M | -0.002 |
| C25M | 0.047 |
| C26M | 0.029 |
| C27M | -0.076 |
| C28M | 0.175 |
| C29M | -0.096 |
| C30M | 0.084 |
| B1m | 0.001 |
| B2m | -0.061 |
| B3m | -0.014 |
| B4m | -0.099 |
| B5m | -0.084 |
| B6m | 0.055 |
| B7m | 0.165 |
| B8m | 0.006 |
| B9m | -0.019 |
| B10m | -0.064 |
| B11m | 0.213 |
| B12m | 0.090 |
| B13m | 0.090 |
| B14m | 0.047 |
| B15m | -0.084 |
| B16m | 0.037 |
| B17m | 0.084 |
| B18m | -0.015 |
| B19m | 0.059 |
| B20m | 0.087 |
| B21m | 0.214 |
| B22m | 0.008 |
| B23m | 0.100 |
| B24m | 0.013 |
| B25m | -0.049 |
| B26m | -0.032 |
| B27m | 0.051 |
| B28m | -0.076 |
| B29m | 0.338 |
| B30m | -0.030 |
