# Supplementary material for: Questioning inbreeding: Could outbreeding affect productivity in the North African catfish in Thailand?
Source: PLoS One. 2024 May 6;19(5):e0302584. doi: 10.1371/journal.pone.0302584 (PMC11073742; doi:10.1371/journal.pone.0302584)
Supplement: S9 Table — (DOCX) [file pone.0302584.s009.docx]

**S9 Table.** Inbreeding coefficients (*F*_IS_) of 31 individuals from the Nakhon Nayok population.

| **Individual** | ***F*_IS_** |
| --- | --- |
| N1F | -0.043 |
| N2M | -0.003 |
| N3F | 0.153 |
| N4M | 0.133 |
| N5M | 0.199 |
| N6M | 0.014 |
| N7F | -0.064 |
| N8F | 0.133 |
| N9F | 0.023 |
| N10F | -0.006 |
| N11M | 0.195 |
| N12M | 0.074 |
| N13F | -0.010 |
| N15F | 0.187 |
| N16M | 0.205 |
| N17F | -0.049 |
| N18F | 0.090 |
| N19F | 0.045 |
| N20F | -0.035 |
| N21F | -0.040 |
| N22F | -0.035 |
| N23F | 0.006 |
| N24F | -0.040 |
| N25M | -0.028 |
| N26M | 0.078 |
| N27M | 0.086 |
| N28M | 0.192 |
| N29M | -0.048 |
| N30M | 0.046 |
| N31M | 0.080 |
| N32M | -0.063 |
