# Supplementary material for: Questioning inbreeding: Could outbreeding affect productivity in the North African catfish in Thailand?
Source: PLoS One. 2024 May 6;19(5):e0302584. doi: 10.1371/journal.pone.0302584 (PMC11073742; doi:10.1371/journal.pone.0302584)
Supplement: S10 Table — (DOCX) [file pone.0302584.s010.docx]

**S10 Table.** Distributions of genetic relatedness values (*r*) and inbreeding coefficients (*F*_IS_) for the North African catfish (*Clarias gariepinus*).

| **Population 1*** | **Population 2*** | **Relatedness value (*r*)** | | **Inbreeding coefficient (*F*_IS_)** | |
| --- | --- | --- | --- | --- | --- |
|  |  | **Density** | ***p-*value** | **Density** | ***p-*value** |
| all populations | SBR | 0.540 | <0.01 | 0.279 | 0.519 |
| all populations | KSN | 0.013 | 0.838 | 0.124 | 0.810 |
| all populations | NYK | 0.101 | <0.01 | 0.088 | 0.974 |
| SBR | KSN | 0.550 | <0.01 | 0.287 | 0.483 |
| SBR | NYK | 0.476 | <0.01 | 0.327 | 0.422 |
| KSN | NYK | 0.113 | <0.01 | 0.035 | 1.000 |

*****SBR, Sing Buri; KSN, Kalasin; NYK, Nakhon Nayok.
