# Supplementary material for: Questioning inbreeding: Could outbreeding affect productivity in the North African catfish in Thailand?
Source: PLoS One. 2024 May 6;19(5):e0302584. doi: 10.1371/journal.pone.0302584 (PMC11073742; doi:10.1371/journal.pone.0302584)
Supplement: S11 Table — (DOCX) [file pone.0302584.s011.docx]

**S11 Table.** Pairwise comparison of genetic relatedness (*r*) values for eight individuals from the Sing Buri population.

| **Sample 1** | **Sample 2** | ***r*** |
| --- | --- | --- |
| S1M1 | S2M2 | -0.146 |
| S1M1 | S3M3 | 0.011 |
| S2M2 | S3M3 | -0.034 |
| S1M1 | S4M4 | -0.087 |
| S2M2 | S4M4 | 0.019 |
| S3M3 | S4M4 | -0.054 |
| S1M1 | S5M5 | -0.072 |
| S2M2 | S5M5 | -0.214 |
| S3M3 | S5M5 | -0.151 |
| S4M4 | S5M5 | -0.174 |
| S1M1 | S6M6 | -0.163 |
| S2M2 | S6M6 | -0.120 |
| S3M3 | S6M6 | -0.003 |
| S4M4 | S6M6 | -0.099 |
| S5M5 | S6M6 | -0.123 |
| S1M1 | S7M7 | 0.010 |
| S2M2 | S7M7 | -0.097 |
| S3M3 | S7M7 | 0.005 |
| S4M4 | S7M7 | -0.077 |
| S5M5 | S7M7 | -0.166 |
| S6M6 | S7M7 | -0.143 |
| S1M1 | S8M8 | -0.140 |
| S2M2 | S8M8 | -0.159 |
| S3M3 | S8M8 | -0.223 |
| S4M4 | S8M8 | -0.062 |
| S5M5 | S8M8 | -0.178 |
| S6M6 | S8M8 | -0.059 |
