# Supplementary material for: Questioning inbreeding: Could outbreeding affect productivity in the North African catfish in Thailand?
Source: PLoS One. 2024 May 6;19(5):e0302584. doi: 10.1371/journal.pone.0302584 (PMC11073742; doi:10.1371/journal.pone.0302584)
Supplement: S12 Table — (DOCX) [file pone.0302584.s012.docx]

**S12 Table.** Pairwise comparison of genetic relatedness (*r*) values for 97 individuals from the Kalasin population.

| **Sample 1** | **Sample 2** | ***r*** |
| --- | --- | --- |
| B1 | B2 | 0.080 |
| B1 | B3 | -0.090 |
| B2 | B3 | -0.085 |
| B1 | B4 | -0.029 |
| B2 | B4 | -0.079 |
| B3 | B4 | -0.067 |
| B1 | B5 | 0.000 |
| B2 | B5 | 0.019 |
| B3 | B5 | -0.070 |
| B4 | B5 | 0.096 |
| B1 | B6 | 0.073 |
| B2 | B6 | -0.070 |
| B3 | B6 | 0.003 |
| B4 | B6 | 0.066 |
| B5 | B6 | 0.023 |
| B1 | B7 | 0.088 |
| B2 | B7 | -0.064 |
| B3 | B7 | -0.048 |
| B4 | B7 | 0.103 |
| B5 | B7 | 0.022 |
| B6 | B7 | 0.144 |
| B1 | B8F | 0.002 |
| B2 | B8F | -0.075 |
| B3 | B8F | -0.098 |
| B4 | B8F | -0.035 |
| B5 | B8F | -0.078 |
| B6 | B8F | 0.080 |
| B7 | B8F | 0.039 |
| B1 | B9F | -0.003 |
| B2 | B9F | -0.097 |
| B3 | B9F | -0.104 |
| B4 | B9F | -0.048 |
| B5 | B9F | -0.019 |
| B6 | B9F | 0.100 |
| B7 | B9F | -0.012 |
| B8F | B9F | 0.242 |
| B1 | B10F | 0.283 |
| B2 | B10F | -0.005 |
| B3 | B10F | 0.378 |
| B4 | B10F | -0.032 |
| B5 | B10F | -0.088 |
| B6 | B10F | 0.062 |
| B7 | B10F | 0.090 |
| B8F | B10F | 0.013 |
| B9F | B10F | 0.104 |
| B1 | B11F | -0.067 |
| B2 | B11F | -0.021 |
| B3 | B11F | -0.073 |
| B4 | B11F | -0.023 |
| B5 | B11F | 0.014 |
| B6 | B11F | 0.003 |
| B7 | B11F | 0.025 |
| B8F | B11F | 0.041 |
| B9F | B11F | -0.038 |
| B10F | B11F | -0.047 |
| B1 | B12F | -0.036 |
| B2 | B12F | -0.061 |
| B3 | B12F | 0.113 |
| B4 | B12F | -0.073 |
| B5 | B12F | 0.041 |
| B6 | B12F | -0.093 |
| B7 | B12F | -0.077 |
| B8F | B12F | -0.096 |
| B9F | B12F | -0.050 |
| B10F | B12F | -0.059 |
| B11F | B12F | -0.083 |
| B1 | B13M | -0.021 |
| B2 | B13M | 0.008 |
| B3 | B13M | -0.065 |
| B4 | B13M | 0.020 |
| B5 | B13M | -0.069 |
| B6 | B13M | -0.024 |
| B7 | B13M | 0.019 |
| B8F | B13M | -0.057 |
| B9F | B13M | -0.061 |
| B10F | B13M | 0.031 |
| B11F | B13M | -0.080 |
| B12F | B13M | 0.033 |
| B1 | B14F | -0.053 |
| B2 | B14F | -0.011 |
| B3 | B14F | -0.033 |
| B4 | B14F | 0.150 |
| B5 | B14F | -0.025 |
| B6 | B14F | 0.074 |
| B7 | B14F | 0.066 |
| B8F | B14F | 0.060 |
| B9F | B14F | 0.030 |
| B10F | B14F | -0.042 |
| B11F | B14F | 0.045 |
| B12F | B14F | -0.056 |
| B13M | B14F | -0.068 |
| B1 | B15M | 0.011 |
| B2 | B15M | 0.030 |
| B3 | B15M | 0.046 |
| B4 | B15M | -0.064 |
| B5 | B15M | -0.018 |
| B6 | B15M | 0.015 |
| B7 | B15M | 0.015 |
| B8F | B15M | -0.004 |
| B9F | B15M | -0.038 |
| B10F | B15M | -0.024 |
| B11F | B15M | -0.025 |
| B12F | B15M | -0.024 |
| B13M | B15M | 0.001 |
| B14F | B15M | -0.017 |
| B1 | B16F | -0.108 |
| B2 | B16F | -0.065 |
| B3 | B16F | 0.082 |
| B4 | B16F | 0.005 |
| B5 | B16F | -0.039 |
| B6 | B16F | -0.041 |
| B7 | B16F | -0.052 |
| B8F | B16F | -0.044 |
| B9F | B16F | -0.002 |
| B10F | B16F | 0.013 |
| B11F | B16F | -0.022 |
| B12F | B16F | 0.117 |
| B13M | B16F | -0.006 |
| B14F | B16F | -0.020 |
| B15M | B16F | 0.036 |
| B1 | B17F | -0.040 |
| B2 | B17F | -0.085 |
| B3 | B17F | -0.050 |
| B4 | B17F | 0.004 |
| B5 | B17F | -0.028 |
| B6 | B17F | -0.014 |
| B7 | B17F | 0.081 |
| B8F | B17F | -0.010 |
| B9F | B17F | 0.063 |
| B10F | B17F | -0.021 |
| B11F | B17F | -0.022 |
| B12F | B17F | 0.047 |
| B13M | B17F | -0.007 |
| B14F | B17F | 0.043 |
| B15M | B17F | 0.112 |
| B16F | B17F | 0.041 |
| B1 | B18M | -0.095 |
| B2 | B18M | 0.042 |
| B3 | B18M | 0.034 |
| B4 | B18M | -0.061 |
| B5 | B18M | 0.007 |
| B6 | B18M | -0.103 |
| B7 | B18M | -0.019 |
| B8F | B18M | -0.075 |
| B9F | B18M | 0.037 |
| B10F | B18M | -0.071 |
| B11F | B18M | -0.060 |
| B12F | B18M | 0.072 |
| B13M | B18M | -0.053 |
| B14F | B18M | -0.009 |
| B15M | B18M | -0.047 |
| B16F | B18M | 0.065 |
| B17F | B18M | 0.077 |
| B1 | B19M | 0.162 |
| B2 | B19M | -0.027 |
| B3 | B19M | -0.025 |
| B4 | B19M | -0.037 |
| B5 | B19M | 0.095 |
| B6 | B19M | 0.199 |
| B7 | B19M | 0.054 |
| B8F | B19M | -0.116 |
| B9F | B19M | -0.062 |
| B10F | B19M | 0.026 |
| B11F | B19M | -0.078 |
| B12F | B19M | -0.005 |
| B13M | B19M | 0.069 |
| B14F | B19M | -0.054 |
| B15M | B19M | 0.033 |
| B16F | B19M | 0.023 |
| B17F | B19M | 0.000 |
| B18M | B19M | 0.192 |
| B1 | B20F | 0.020 |
| B2 | B20F | -0.017 |
| B3 | B20F | -0.064 |
| B4 | B20F | -0.001 |
| B5 | B20F | 0.094 |
| B6 | B20F | 0.108 |
| B7 | B20F | -0.003 |
| B8F | B20F | 0.022 |
| B9F | B20F | 0.111 |
| B10F | B20F | -0.097 |
| B11F | B20F | -0.007 |
| B12F | B20F | -0.036 |
| B13M | B20F | -0.078 |
| B14F | B20F | 0.092 |
| B15M | B20F | -0.062 |
| B16F | B20F | -0.044 |
| B17F | B20F | -0.061 |
| B18M | B20F | 0.064 |
| B19M | B20F | 0.029 |
| B1 | B21F | -0.091 |
| B2 | B21F | -0.070 |
| B3 | B21F | -0.011 |
| B4 | B21F | 0.088 |
| B5 | B21F | 0.043 |
| B6 | B21F | -0.011 |
| B7 | B21F | -0.031 |
| B8F | B21F | -0.053 |
| B9F | B21F | 0.000 |
| B10F | B21F | -0.054 |
| B11F | B21F | -0.040 |
| B12F | B21F | -0.040 |
| B13M | B21F | -0.043 |
| B14F | B21F | 0.042 |
| B15M | B21F | -0.083 |
| B16F | B21F | 0.141 |
| B17F | B21F | 0.042 |
| B18M | B21F | -0.025 |
| B19M | B21F | -0.034 |
| B20F | B21F | -0.019 |
| B1 | B22F | -0.013 |
| B2 | B22F | -0.079 |
| B3 | B22F | 0.055 |
| B4 | B22F | -0.042 |
| B5 | B22F | -0.057 |
| B6 | B22F | 0.027 |
| B7 | B22F | 0.097 |
| B8F | B22F | -0.027 |
| B9F | B22F | 0.032 |
| B10F | B22F | 0.040 |
| B11F | B22F | -0.021 |
| B12F | B22F | -0.025 |
| B13M | B22F | -0.052 |
| B14F | B22F | 0.048 |
| B15M | B22F | -0.032 |
| B16F | B22F | 0.162 |
| B17F | B22F | 0.026 |
| B18M | B22F | 0.207 |
| B19M | B22F | 0.218 |
| B20F | B22F | 0.000 |
| B21F | B22F | 0.005 |
| B1 | B23F | 0.238 |
| B2 | B23F | 0.192 |
| B3 | B23F | 0.312 |
| B4 | B23F | 0.141 |
| B5 | B23F | 0.157 |
| B6 | B23F | -0.063 |
| B7 | B23F | 0.001 |
| B8F | B23F | -0.002 |
| B9F | B23F | -0.037 |
| B10F | B23F | -0.011 |
| B11F | B23F | -0.008 |
| B12F | B23F | -0.022 |
| B13M | B23F | -0.050 |
| B14F | B23F | 0.061 |
| B15M | B23F | 0.073 |
| B16F | B23F | -0.070 |
| B17F | B23F | -0.022 |
| B18M | B23F | -0.023 |
| B19M | B23F | -0.035 |
| B20F | B23F | -0.038 |
| B21F | B23F | -0.053 |
| B22F | B23F | -0.033 |
| B1 | B24F | -0.026 |
| B2 | B24F | -0.028 |
| B3 | B24F | -0.064 |
| B4 | B24F | -0.008 |
| B5 | B24F | -0.002 |
| B6 | B24F | -0.089 |
| B7 | B24F | -0.044 |
| B8F | B24F | 0.013 |
| B9F | B24F | 0.067 |
| B10F | B24F | 0.009 |
| B11F | B24F | -0.063 |
| B12F | B24F | 0.095 |
| B13M | B24F | 0.082 |
| B14F | B24F | 0.004 |
| B15M | B24F | 0.019 |
| B16F | B24F | 0.017 |
| B17F | B24F | 0.219 |
| B18M | B24F | 0.025 |
| B19M | B24F | -0.032 |
| B20F | B24F | -0.059 |
| B21F | B24F | -0.016 |
| B22F | B24F | -0.084 |
| B23F | B24F | 0.012 |
| B1 | B25F | 0.009 |
| B2 | B25F | -0.086 |
| B3 | B25F | -0.092 |
| B4 | B25F | 0.076 |
| B5 | B25F | -0.080 |
| B6 | B25F | 0.039 |
| B7 | B25F | -0.025 |
| B8F | B25F | -0.037 |
| B9F | B25F | -0.049 |
| B10F | B25F | -0.050 |
| B11F | B25F | 0.170 |
| B12F | B25F | -0.016 |
| B13M | B25F | 0.083 |
| B14F | B25F | -0.019 |
| B15M | B25F | -0.014 |
| B16F | B25F | -0.066 |
| B17F | B25F | -0.008 |
| B18M | B25F | -0.135 |
| B19M | B25F | -0.084 |
| B20F | B25F | -0.022 |
| B21F | B25F | 0.058 |
| B22F | B25F | -0.138 |
| B23F | B25F | -0.088 |
| B24F | B25F | 0.059 |
| B1 | B26F | -0.007 |
| B2 | B26F | -0.073 |
| B3 | B26F | 0.001 |
| B4 | B26F | -0.039 |
| B5 | B26F | -0.039 |
| B6 | B26F | 0.053 |
| B7 | B26F | -0.015 |
| B8F | B26F | -0.019 |
| B9F | B26F | 0.100 |
| B10F | B26F | -0.033 |
| B11F | B26F | 0.117 |
| B12F | B26F | 0.040 |
| B13M | B26F | 0.011 |
| B14F | B26F | 0.054 |
| B15M | B26F | -0.021 |
| B16F | B26F | 0.022 |
| B17F | B26F | 0.035 |
| B18M | B26F | -0.013 |
| B19M | B26F | 0.021 |
| B20F | B26F | 0.010 |
| B21F | B26F | 0.001 |
| B22F | B26F | 0.003 |
| B23F | B26F | -0.024 |
| B24F | B26F | 0.019 |
| B25F | B26F | 0.217 |
| B1 | B27F | -0.120 |
| B2 | B27F | -0.087 |
| B3 | B27F | 0.115 |
| B4 | B27F | 0.057 |
| B5 | B27F | -0.039 |
| B6 | B27F | 0.002 |
| B7 | B27F | -0.038 |
| B8F | B27F | 0.004 |
| B9F | B27F | -0.012 |
| B10F | B27F | -0.098 |
| B11F | B27F | -0.008 |
| B12F | B27F | 0.124 |
| B13M | B27F | -0.053 |
| B14F | B27F | 0.079 |
| B15M | B27F | 0.004 |
| B16F | B27F | 0.165 |
| B17F | B27F | -0.008 |
| B18M | B27F | -0.015 |
| B19M | B27F | -0.125 |
| B20F | B27F | -0.072 |
| B21F | B27F | 0.199 |
| B22F | B27F | -0.060 |
| B23F | B27F | -0.045 |
| B24F | B27F | -0.026 |
| B25F | B27F | 0.075 |
| B26F | B27F | 0.074 |
| B1 | B28M | -0.021 |
| B2 | B28M | -0.101 |
| B3 | B28M | -0.073 |
| B4 | B28M | 0.029 |
| B5 | B28M | -0.002 |
| B6 | B28M | -0.057 |
| B7 | B28M | 0.026 |
| B8F | B28M | -0.014 |
| B9F | B28M | 0.041 |
| B10F | B28M | -0.043 |
| B11F | B28M | -0.008 |
| B12F | B28M | 0.015 |
| B13M | B28M | 0.036 |
| B14F | B28M | -0.022 |
| B15M | B28M | 0.042 |
| B16F | B28M | 0.026 |
| B17F | B28M | 0.319 |
| B18M | B28M | 0.024 |
| B19M | B28M | 0.003 |
| B20F | B28M | -0.046 |
| B21F | B28M | 0.011 |
| B22F | B28M | -0.006 |
| B23F | B28M | -0.008 |
| B24F | B28M | 0.003 |
| B25F | B28M | -0.087 |
| B26F | B28M | 0.226 |
| B27F | B28M | 0.003 |
| B1 | B29F | 0.128 |
| B2 | B29F | 0.111 |
| B3 | B29F | 0.290 |
| B4 | B29F | -0.044 |
| B5 | B29F | -0.107 |
| B6 | B29F | 0.092 |
| B7 | B29F | -0.048 |
| B8F | B29F | 0.122 |
| B9F | B29F | 0.138 |
| B10F | B29F | -0.129 |
| B11F | B29F | -0.024 |
| B12F | B29F | -0.095 |
| B13M | B29F | -0.075 |
| B14F | B29F | 0.029 |
| B15M | B29F | -0.151 |
| B16F | B29F | -0.044 |
| B17F | B29F | -0.078 |
| B18M | B29F | 0.005 |
| B19M | B29F | -0.131 |
| B20F | B29F | 0.103 |
| B21F | B29F | -0.081 |
| B22F | B29F | -0.036 |
| B23F | B29F | 0.304 |
| B24F | B29F | -0.077 |
| B25F | B29F | -0.072 |
| B26F | B29F | -0.066 |
| B27F | B29F | -0.072 |
| B28M | B29F | -0.107 |
| B1 | B30F | -0.093 |
| B2 | B30F | -0.004 |
| B3 | B30F | 0.033 |
| B4 | B30F | 0.168 |
| B5 | B30F | 0.021 |
| B6 | B30F | -0.034 |
| B7 | B30F | 0.005 |
| B8F | B30F | -0.074 |
| B9F | B30F | -0.056 |
| B10F | B30F | 0.083 |
| B11F | B30F | -0.079 |
| B12F | B30F | -0.044 |
| B13M | B30F | 0.086 |
| B14F | B30F | 0.085 |
| B15M | B30F | -0.022 |
| B16F | B30F | 0.093 |
| B17F | B30F | -0.008 |
| B18M | B30F | 0.005 |
| B19M | B30F | -0.052 |
| B20F | B30F | -0.063 |
| B21F | B30F | 0.074 |
| B22F | B30F | 0.078 |
| B23F | B30F | 0.155 |
| B24F | B30F | 0.020 |
| B25F | B30F | -0.023 |
| B26F | B30F | -0.001 |
| B27F | B30F | 0.024 |
| B28M | B30F | 0.006 |
| B29F | B30F | -0.037 |
| B1 | B31M | 0.060 |
| B2 | B31M | -0.031 |
| B3 | B31M | -0.017 |
| B4 | B31M | -0.019 |
| B5 | B31M | -0.001 |
| B6 | B31M | -0.030 |
| B7 | B31M | 0.010 |
| B8F | B31M | 0.081 |
| B9F | B31M | -0.119 |
| B10F | B31M | -0.025 |
| B11F | B31M | 0.029 |
| B12F | B31M | -0.033 |
| B13M | B31M | 0.289 |
| B14F | B31M | -0.125 |
| B15M | B31M | -0.010 |
| B16F | B31M | -0.030 |
| B17F | B31M | 0.004 |
| B18M | B31M | -0.030 |
| B19M | B31M | 0.108 |
| B20F | B31M | -0.008 |
| B21F | B31M | -0.018 |
| B22F | B31M | -0.024 |
| B23F | B31M | -0.053 |
| B24F | B31M | 0.013 |
| B25F | B31M | 0.056 |
| B26F | B31M | -0.061 |
| B27F | B31M | -0.090 |
| B28M | B31M | -0.032 |
| B29F | B31M | -0.075 |
| B30F | B31M | 0.047 |
| B1 | B32M | -0.001 |
| B2 | B32M | -0.028 |
| B3 | B32M | 0.023 |
| B4 | B32M | -0.076 |
| B5 | B32M | 0.006 |
| B6 | B32M | 0.015 |
| B7 | B32M | 0.105 |
| B8F | B32M | 0.074 |
| B9F | B32M | 0.112 |
| B10F | B32M | 0.225 |
| B11F | B32M | -0.053 |
| B12F | B32M | -0.007 |
| B13M | B32M | 0.009 |
| B14F | B32M | -0.039 |
| B15M | B32M | -0.062 |
| B16F | B32M | 0.088 |
| B17F | B32M | 0.014 |
| B18M | B32M | 0.077 |
| B19M | B32M | 0.088 |
| B20F | B32M | 0.024 |
| B21F | B32M | -0.060 |
| B22F | B32M | 0.128 |
| B23F | B32M | -0.007 |
| B24F | B32M | 0.041 |
| B25F | B32M | -0.073 |
| B26F | B32M | -0.002 |
| B27F | B32M | -0.124 |
| B28M | B32M | -0.027 |
| B29F | B32M | -0.041 |
| B30F | B32M | -0.002 |
| B31M | B32M | 0.009 |
| B1 | B33M | -0.035 |
| B2 | B33M | -0.030 |
| B3 | B33M | 0.025 |
| B4 | B33M | -0.098 |
| B5 | B33M | -0.021 |
| B6 | B33M | -0.090 |
| B7 | B33M | -0.033 |
| B8F | B33M | -0.029 |
| B9F | B33M | 0.035 |
| B10F | B33M | 0.075 |
| B11F | B33M | -0.043 |
| B12F | B33M | 0.101 |
| B13M | B33M | -0.026 |
| B14F | B33M | -0.087 |
| B15M | B33M | -0.002 |
| B16F | B33M | 0.064 |
| B17F | B33M | 0.125 |
| B18M | B33M | 0.102 |
| B19M | B33M | 0.005 |
| B20F | B33M | -0.084 |
| B21F | B33M | -0.055 |
| B22F | B33M | 0.076 |
| B23F | B33M | -0.009 |
| B24F | B33M | 0.045 |
| B25F | B33M | -0.127 |
| B26F | B33M | 0.000 |
| B27F | B33M | -0.083 |
| B28M | B33M | 0.068 |
| B29F | B33M | 0.404 |
| B30F | B33M | -0.030 |
| B31M | B33M | -0.061 |
| B32M | B33M | 0.162 |
| B1 | B34M | 0.019 |
| B2 | B34M | 0.015 |
| B3 | B34M | 0.161 |
| B4 | B34M | -0.096 |
| B5 | B34M | -0.096 |
| B6 | B34M | -0.044 |
| B7 | B34M | -0.063 |
| B8F | B34M | 0.003 |
| B9F | B34M | -0.041 |
| B10F | B34M | -0.006 |
| B11F | B34M | 0.006 |
| B12F | B34M | 0.022 |
| B13M | B34M | 0.011 |
| B14F | B34M | -0.022 |
| B15M | B34M | 0.063 |
| B16F | B34M | 0.028 |
| B17F | B34M | -0.031 |
| B18M | B34M | -0.010 |
| B19M | B34M | 0.001 |
| B20F | B34M | 0.005 |
| B21F | B34M | -0.074 |
| B22F | B34M | 0.039 |
| B23F | B34M | -0.053 |
| B24F | B34M | -0.061 |
| B25F | B34M | -0.130 |
| B26F | B34M | -0.030 |
| B27F | B34M | -0.050 |
| B28M | B34M | 0.000 |
| B29F | B34M | 0.041 |
| B30F | B34M | 0.015 |
| B31M | B34M | 0.049 |
| B32M | B34M | 0.027 |
| B33M | B34M | 0.048 |
| B1 | B35M | 0.024 |
| B2 | B35M | -0.079 |
| B3 | B35M | 0.059 |
| B4 | B35M | -0.061 |
| B5 | B35M | -0.025 |
| B6 | B35M | -0.057 |
| B7 | B35M | -0.011 |
| B8F | B35M | 0.068 |
| B9F | B35M | 0.071 |
| B10F | B35M | 0.061 |
| B11F | B35M | 0.000 |
| B12F | B35M | 0.273 |
| B13M | B35M | 0.018 |
| B14F | B35M | -0.030 |
| B15M | B35M | -0.065 |
| B16F | B35M | 0.059 |
| B17F | B35M | 0.072 |
| B18M | B35M | 0.028 |
| B19M | B35M | -0.072 |
| B20F | B35M | -0.068 |
| B21F | B35M | 0.005 |
| B22F | B35M | -0.022 |
| B23F | B35M | 0.006 |
| B24F | B35M | 0.058 |
| B25F | B35M | -0.012 |
| B26F | B35M | -0.021 |
| B27F | B35M | 0.044 |
| B28M | B35M | -0.028 |
| B29F | B35M | 0.230 |
| B30F | B35M | -0.061 |
| B31M | B35M | -0.042 |
| B32M | B35M | 0.077 |
| B33M | B35M | 0.329 |
| B34M | B35M | 0.029 |
| B1 | B36M | 0.181 |
| B2 | B36M | -0.059 |
| B3 | B36M | -0.007 |
| B4 | B36M | -0.047 |
| B5 | B36M | -0.019 |
| B6 | B36M | 0.077 |
| B7 | B36M | -0.005 |
| B8F | B36M | -0.041 |
| B9F | B36M | -0.052 |
| B10F | B36M | -0.041 |
| B11F | B36M | 0.103 |
| B12F | B36M | -0.010 |
| B13M | B36M | -0.080 |
| B14F | B36M | 0.080 |
| B15M | B36M | 0.032 |
| B16F | B36M | -0.076 |
| B17F | B36M | -0.008 |
| B18M | B36M | -0.024 |
| B19M | B36M | 0.136 |
| B20F | B36M | 0.070 |
| B21F | B36M | -0.045 |
| B22F | B36M | 0.210 |
| B23F | B36M | 0.082 |
| B24F | B36M | -0.056 |
| B25F | B36M | -0.012 |
| B26F | B36M | 0.003 |
| B27F | B36M | -0.051 |
| B28M | B36M | -0.024 |
| B29F | B36M | -0.073 |
| B30F | B36M | -0.031 |
| B31M | B36M | 0.121 |
| B32M | B36M | -0.052 |
| B33M | B36M | -0.062 |
| B34M | B36M | 0.017 |
| B35M | B36M | -0.068 |
| B1 | B37M | -0.059 |
| B2 | B37M | -0.015 |
| B3 | B37M | -0.104 |
| B4 | B37M | 0.067 |
| B5 | B37M | -0.082 |
| B6 | B37M | -0.080 |
| B7 | B37M | 0.031 |
| B8F | B37M | 0.017 |
| B9F | B37M | 0.072 |
| B10F | B37M | 0.048 |
| B11F | B37M | 0.046 |
| B12F | B37M | -0.028 |
| B13M | B37M | 0.145 |
| B14F | B37M | 0.081 |
| B15M | B37M | -0.013 |
| B16F | B37M | -0.026 |
| B17F | B37M | 0.203 |
| B18M | B37M | 0.070 |
| B19M | B37M | -0.045 |
| B20F | B37M | -0.027 |
| B21F | B37M | 0.003 |
| B22F | B37M | -0.059 |
| B23F | B37M | -0.053 |
| B24F | B37M | 0.119 |
| B25F | B37M | -0.013 |
| B26F | B37M | 0.088 |
| B27F | B37M | -0.059 |
| B28M | B37M | 0.065 |
| B29F | B37M | -0.037 |
| B30F | B37M | 0.064 |
| B31M | B37M | 0.064 |
| B32M | B37M | -0.019 |
| B33M | B37M | 0.017 |
| B34M | B37M | -0.008 |
| B35M | B37M | 0.049 |
| B36M | B37M | -0.053 |
| B1 | C1M | 0.031 |
| B2 | C1M | -0.035 |
| B3 | C1M | -0.071 |
| B4 | C1M | 0.023 |
| B5 | C1M | 0.096 |
| B6 | C1M | -0.068 |
| B7 | C1M | -0.007 |
| B8F | C1M | -0.035 |
| B9F | C1M | -0.100 |
| B10F | C1M | -0.063 |
| B11F | C1M | 0.009 |
| B12F | C1M | -0.061 |
| B13M | C1M | 0.020 |
| B14F | C1M | -0.069 |
| B15M | C1M | 0.027 |
| B16F | C1M | -0.066 |
| B17F | C1M | -0.048 |
| B18M | C1M | 0.175 |
| B19M | C1M | 0.165 |
| B20F | C1M | -0.009 |
| B21F | C1M | -0.040 |
| B22F | C1M | 0.137 |
| B23F | C1M | 0.116 |
| B24F | C1M | -0.002 |
| B25F | C1M | -0.032 |
| B26F | C1M | -0.081 |
| B27F | C1M | -0.087 |
| B28M | C1M | -0.033 |
| B29F | C1M | -0.104 |
| B30F | C1M | -0.009 |
| B31M | C1M | 0.043 |
| B32M | C1M | -0.050 |
| B33M | C1M | -0.104 |
| B34M | C1M | 0.004 |
| B35M | C1M | -0.063 |
| B36M | C1M | 0.074 |
| B37M | C1M | -0.003 |
| B1 | C2M | -0.068 |
| B2 | C2M | -0.004 |
| B3 | C2M | -0.055 |
| B4 | C2M | 0.008 |
| B5 | C2M | -0.060 |
| B6 | C2M | 0.000 |
| B7 | C2M | -0.058 |
| B8F | C2M | 0.039 |
| B9F | C2M | 0.064 |
| B10F | C2M | 0.074 |
| B11F | C2M | -0.005 |
| B12F | C2M | -0.041 |
| B13M | C2M | -0.074 |
| B14F | C2M | 0.034 |
| B15M | C2M | -0.039 |
| B16F | C2M | -0.047 |
| B17F | C2M | -0.095 |
| B18M | C2M | -0.087 |
| B19M | C2M | -0.098 |
| B20F | C2M | -0.034 |
| B21F | C2M | -0.019 |
| B22F | C2M | 0.125 |
| B23F | C2M | -0.083 |
| B24F | C2M | -0.130 |
| B25F | C2M | -0.037 |
| B26F | C2M | -0.065 |
| B27F | C2M | -0.008 |
| B28M | C2M | -0.046 |
| B29F | C2M | 0.024 |
| B30F | C2M | -0.024 |
| B31M | C2M | -0.094 |
| B32M | C2M | -0.042 |
| B33M | C2M | -0.001 |
| B34M | C2M | 0.037 |
| B35M | C2M | -0.033 |
| B36M | C2M | 0.089 |
| B37M | C2M | -0.108 |
| C1M | C2M | -0.033 |
| B1 | C3M | -0.019 |
| B2 | C3M | 0.042 |
| B3 | C3M | -0.086 |
| B4 | C3M | -0.061 |
| B5 | C3M | 0.042 |
| B6 | C3M | -0.083 |
| B7 | C3M | -0.032 |
| B8F | C3M | -0.037 |
| B9F | C3M | -0.071 |
| B10F | C3M | -0.001 |
| B11F | C3M | 0.077 |
| B12F | C3M | 0.023 |
| B13M | C3M | 0.025 |
| B14F | C3M | -0.111 |
| B15M | C3M | -0.092 |
| B16F | C3M | 0.076 |
| B17F | C3M | -0.035 |
| B18M | C3M | -0.026 |
| B19M | C3M | -0.013 |
| B20F | C3M | -0.052 |
| B21F | C3M | 0.103 |
| B22F | C3M | 0.017 |
| B23F | C3M | -0.035 |
| B24F | C3M | -0.012 |
| B25F | C3M | -0.035 |
| B26F | C3M | -0.084 |
| B27F | C3M | 0.065 |
| B28M | C3M | -0.015 |
| B29F | C3M | -0.059 |
| B30F | C3M | -0.094 |
| B31M | C3M | 0.048 |
| B32M | C3M | 0.008 |
| B33M | C3M | 0.000 |
| B34M | C3M | -0.001 |
| B35M | C3M | 0.054 |
| B36M | C3M | 0.045 |
| B37M | C3M | -0.031 |
| C1M | C3M | 0.073 |
| C2M | C3M | 0.076 |
| B1 | C4M | 0.077 |
| B2 | C4M | -0.004 |
| B3 | C4M | -0.064 |
| B4 | C4M | -0.027 |
| B5 | C4M | -0.065 |
| B6 | C4M | 0.016 |
| B7 | C4M | 0.026 |
| B8F | C4M | -0.032 |
| B9F | C4M | -0.038 |
| B10F | C4M | -0.017 |
| B11F | C4M | -0.043 |
| B12F | C4M | -0.008 |
| B13M | C4M | 0.128 |
| B14F | C4M | 0.030 |
| B15M | C4M | -0.015 |
| B16F | C4M | 0.054 |
| B17F | C4M | 0.111 |
| B18M | C4M | -0.001 |
| B19M | C4M | 0.037 |
| B20F | C4M | -0.090 |
| B21F | C4M | 0.004 |
| B22F | C4M | 0.011 |
| B23F | C4M | -0.020 |
| B24F | C4M | 0.096 |
| B25F | C4M | 0.084 |
| B26F | C4M | 0.002 |
| B27F | C4M | 0.026 |
| B28M | C4M | 0.002 |
| B29F | C4M | 0.011 |
| B30F | C4M | 0.007 |
| B31M | C4M | 0.134 |
| B32M | C4M | -0.013 |
| B33M | C4M | -0.036 |
| B34M | C4M | 0.028 |
| B35M | C4M | 0.014 |
| B36M | C4M | -0.003 |
| B37M | C4M | 0.014 |
| C1M | C4M | 0.021 |
| C2M | C4M | -0.027 |
| C3M | C4M | 0.060 |
| B1 | C5M | -0.016 |
| B2 | C5M | -0.044 |
| B3 | C5M | -0.047 |
| B4 | C5M | 0.031 |
| B5 | C5M | -0.024 |
| B6 | C5M | -0.058 |
| B7 | C5M | -0.071 |
| B8F | C5M | -0.058 |
| B9F | C5M | -0.062 |
| B10F | C5M | 0.083 |
| B11F | C5M | -0.053 |
| B12F | C5M | 0.021 |
| B13M | C5M | 0.086 |
| B14F | C5M | -0.049 |
| B15M | C5M | -0.018 |
| B16F | C5M | -0.020 |
| B17F | C5M | 0.018 |
| B18M | C5M | -0.062 |
| B19M | C5M | -0.024 |
| B20F | C5M | 0.024 |
| B21F | C5M | 0.013 |
| B22F | C5M | -0.094 |
| B23F | C5M | -0.058 |
| B24F | C5M | 0.079 |
| B25F | C5M | 0.106 |
| B26F | C5M | -0.117 |
| B27F | C5M | 0.020 |
| B28M | C5M | -0.011 |
| B29F | C5M | -0.107 |
| B30F | C5M | 0.017 |
| B31M | C5M | 0.127 |
| B32M | C5M | 0.007 |
| B33M | C5M | -0.053 |
| B34M | C5M | -0.005 |
| B35M | C5M | -0.008 |
| B36M | C5M | 0.046 |
| B37M | C5M | 0.108 |
| C1M | C5M | 0.019 |
| C2M | C5M | 0.081 |
| C3M | C5M | 0.020 |
| C4M | C5M | 0.029 |
| B1 | C6M | -0.104 |
| B2 | C6M | -0.051 |
| B3 | C6M | -0.087 |
| B4 | C6M | 0.022 |
| B5 | C6M | -0.053 |
| B6 | C6M | -0.093 |
| B7 | C6M | -0.092 |
| B8F | C6M | -0.073 |
| B9F | C6M | 0.109 |
| B10F | C6M | 0.090 |
| B11F | C6M | 0.056 |
| B12F | C6M | 0.060 |
| B13M | C6M | 0.104 |
| B14F | C6M | -0.020 |
| B15M | C6M | 0.000 |
| B16F | C6M | -0.051 |
| B17F | C6M | 0.026 |
| B18M | C6M | 0.009 |
| B19M | C6M | -0.080 |
| B20F | C6M | 0.066 |
| B21F | C6M | -0.090 |
| B22F | C6M | -0.082 |
| B23F | C6M | -0.090 |
| B24F | C6M | 0.100 |
| B25F | C6M | 0.071 |
| B26F | C6M | -0.015 |
| B27F | C6M | -0.062 |
| B28M | C6M | -0.031 |
| B29F | C6M | -0.046 |
| B30F | C6M | -0.070 |
| B31M | C6M | -0.026 |
| B32M | C6M | -0.082 |
| B33M | C6M | -0.001 |
| B34M | C6M | -0.087 |
| B35M | C6M | -0.009 |
| B36M | C6M | -0.097 |
| B37M | C6M | 0.114 |
| C1M | C6M | -0.049 |
| C2M | C6M | -0.045 |
| C3M | C6M | -0.080 |
| C4M | C6M | 0.006 |
| C5M | C6M | 0.052 |
| B1 | C7M | -0.005 |
| B2 | C7M | 0.065 |
| B3 | C7M | -0.025 |
| B4 | C7M | -0.038 |
| B5 | C7M | -0.003 |
| B6 | C7M | -0.061 |
| B7 | C7M | -0.028 |
| B8F | C7M | -0.013 |
| B9F | C7M | -0.062 |
| B10F | C7M | -0.082 |
| B11F | C7M | -0.051 |
| B12F | C7M | 0.007 |
| B13M | C7M | -0.016 |
| B14F | C7M | -0.010 |
| B15M | C7M | -0.054 |
| B16F | C7M | -0.028 |
| B17F | C7M | -0.087 |
| B18M | C7M | 0.000 |
| B19M | C7M | -0.049 |
| B20F | C7M | -0.010 |
| B21F | C7M | 0.052 |
| B22F | C7M | 0.014 |
| B23F | C7M | -0.058 |
| B24F | C7M | -0.051 |
| B25F | C7M | -0.032 |
| B26F | C7M | -0.039 |
| B27F | C7M | 0.060 |
| B28M | C7M | -0.099 |
| B29F | C7M | -0.005 |
| B30F | C7M | -0.031 |
| B31M | C7M | 0.006 |
| B32M | C7M | -0.089 |
| B33M | C7M | 0.021 |
| B34M | C7M | 0.091 |
| B35M | C7M | 0.014 |
| B36M | C7M | -0.011 |
| B37M | C7M | -0.044 |
| C1M | C7M | -0.040 |
| C2M | C7M | 0.064 |
| C3M | C7M | 0.101 |
| C4M | C7M | 0.035 |
| C5M | C7M | -0.031 |
| C6M | C7M | -0.019 |
| B1 | C8M | 0.021 |
| B2 | C8M | 0.026 |
| B3 | C8M | -0.057 |
| B4 | C8M | -0.041 |
| B5 | C8M | -0.042 |
| B6 | C8M | -0.085 |
| B7 | C8M | -0.048 |
| B8F | C8M | -0.104 |
| B9F | C8M | -0.007 |
| B10F | C8M | -0.062 |
| B11F | C8M | -0.025 |
| B12F | C8M | -0.025 |
| B13M | C8M | 0.044 |
| B14F | C8M | 0.081 |
| B15M | C8M | 0.049 |
| B16F | C8M | -0.051 |
| B17F | C8M | -0.009 |
| B18M | C8M | 0.070 |
| B19M | C8M | 0.017 |
| B20F | C8M | 0.119 |
| B21F | C8M | -0.004 |
| B22F | C8M | -0.039 |
| B23F | C8M | -0.056 |
| B24F | C8M | -0.056 |
| B25F | C8M | -0.121 |
| B26F | C8M | -0.011 |
| B27F | C8M | -0.044 |
| B28M | C8M | 0.004 |
| B29F | C8M | 0.019 |
| B30F | C8M | 0.019 |
| B31M | C8M | -0.011 |
| B32M | C8M | -0.084 |
| B33M | C8M | 0.022 |
| B34M | C8M | 0.088 |
| B35M | C8M | -0.029 |
| B36M | C8M | -0.027 |
| B37M | C8M | 0.043 |
| C1M | C8M | 0.024 |
| C2M | C8M | -0.017 |
| C3M | C8M | -0.009 |
| C4M | C8M | 0.032 |
| C5M | C8M | -0.056 |
| C6M | C8M | 0.256 |
| C7M | C8M | 0.064 |
| B1 | C9M | 0.008 |
| B2 | C9M | -0.014 |
| B3 | C9M | -0.048 |
| B4 | C9M | -0.030 |
| B5 | C9M | 0.041 |
| B6 | C9M | 0.012 |
| B7 | C9M | 0.016 |
| B8F | C9M | 0.031 |
| B9F | C9M | -0.032 |
| B10F | C9M | -0.053 |
| B11F | C9M | 0.008 |
| B12F | C9M | -0.049 |
| B13M | C9M | -0.038 |
| B14F | C9M | 0.006 |
| B15M | C9M | 0.022 |
| B16F | C9M | -0.085 |
| B17F | C9M | -0.067 |
| B18M | C9M | 0.007 |
| B19M | C9M | 0.072 |
| B20F | C9M | 0.018 |
| B21F | C9M | -0.031 |
| B22F | C9M | -0.016 |
| B23F | C9M | -0.076 |
| B24F | C9M | -0.086 |
| B25F | C9M | -0.102 |
| B26F | C9M | -0.097 |
| B27F | C9M | -0.052 |
| B28M | C9M | -0.031 |
| B29F | C9M | -0.028 |
| B30F | C9M | -0.048 |
| B31M | C9M | -0.010 |
| B32M | C9M | -0.030 |
| B33M | C9M | -0.069 |
| B34M | C9M | 0.085 |
| B35M | C9M | 0.001 |
| B36M | C9M | 0.026 |
| B37M | C9M | -0.032 |
| C1M | C9M | 0.069 |
| C2M | C9M | 0.159 |
| C3M | C9M | -0.008 |
| C4M | C9M | -0.024 |
| C5M | C9M | 0.120 |
| C6M | C9M | -0.103 |
| C7M | C9M | 0.010 |
| C8M | C9M | 0.040 |
| B1 | C10M | 0.036 |
| B2 | C10M | -0.092 |
| B3 | C10M | -0.083 |
| B4 | C10M | -0.002 |
| B5 | C10M | 0.027 |
| B6 | C10M | 0.044 |
| B7 | C10M | -0.012 |
| B8F | C10M | 0.086 |
| B9F | C10M | 0.030 |
| B10F | C10M | -0.144 |
| B11F | C10M | 0.021 |
| B12F | C10M | 0.013 |
| B13M | C10M | 0.004 |
| B14F | C10M | 0.014 |
| B15M | C10M | -0.006 |
| B16F | C10M | -0.001 |
| B17F | C10M | 0.021 |
| B18M | C10M | -0.072 |
| B19M | C10M | 0.003 |
| B20F | C10M | 0.109 |
| B21F | C10M | -0.006 |
| B22F | C10M | -0.047 |
| B23F | C10M | -0.015 |
| B24F | C10M | 0.001 |
| B25F | C10M | 0.015 |
| B26F | C10M | 0.030 |
| B27F | C10M | 0.024 |
| B28M | C10M | 0.027 |
| B29F | C10M | -0.012 |
| B30F | C10M | -0.068 |
| B31M | C10M | 0.019 |
| B32M | C10M | 0.013 |
| B33M | C10M | -0.035 |
| B34M | C10M | -0.008 |
| B35M | C10M | -0.042 |
| B36M | C10M | 0.080 |
| B37M | C10M | 0.051 |
| C1M | C10M | -0.022 |
| C2M | C10M | -0.096 |
| C3M | C10M | -0.023 |
| C4M | C10M | -0.083 |
| C5M | C10M | 0.106 |
| C6M | C10M | -0.050 |
| C7M | C10M | -0.011 |
| C8M | C10M | -0.081 |
| C9M | C10M | -0.014 |
| B1 | C11M | -0.026 |
| B2 | C11M | -0.004 |
| B3 | C11M | 0.082 |
| B4 | C11M | -0.032 |
| B5 | C11M | -0.005 |
| B6 | C11M | 0.014 |
| B7 | C11M | -0.081 |
| B8F | C11M | -0.035 |
| B9F | C11M | -0.077 |
| B10F | C11M | -0.042 |
| B11F | C11M | 0.007 |
| B12F | C11M | -0.007 |
| B13M | C11M | -0.046 |
| B14F | C11M | -0.022 |
| B15M | C11M | 0.097 |
| B16F | C11M | 0.010 |
| B17F | C11M | -0.037 |
| B18M | C11M | -0.049 |
| B19M | C11M | -0.034 |
| B20F | C11M | -0.036 |
| B21F | C11M | 0.021 |
| B22F | C11M | -0.014 |
| B23F | C11M | -0.013 |
| B24F | C11M | -0.047 |
| B25F | C11M | -0.025 |
| B26F | C11M | -0.065 |
| B27F | C11M | 0.057 |
| B28M | C11M | -0.060 |
| B29F | C11M | -0.022 |
| B30F | C11M | -0.019 |
| B31M | C11M | 0.013 |
| B32M | C11M | -0.051 |
| B33M | C11M | 0.009 |
| B34M | C11M | 0.130 |
| B35M | C11M | -0.006 |
| B36M | C11M | 0.024 |
| B37M | C11M | -0.066 |
| C1M | C11M | 0.030 |
| C2M | C11M | -0.014 |
| C3M | C11M | 0.037 |
| C4M | C11M | 0.041 |
| C5M | C11M | 0.035 |
| C6M | C11M | -0.064 |
| C7M | C11M | 0.057 |
| C8M | C11M | 0.015 |
| C9M | C11M | 0.028 |
| C10M | C11M | -0.003 |
| B1 | C12M | -0.007 |
| B2 | C12M | -0.133 |
| B3 | C12M | -0.111 |
| B4 | C12M | -0.014 |
| B5 | C12M | -0.077 |
| B6 | C12M | 0.017 |
| B7 | C12M | 0.084 |
| B8F | C12M | 0.126 |
| B9F | C12M | 0.165 |
| B10F | C12M | 0.080 |
| B11F | C12M | 0.021 |
| B12F | C12M | -0.037 |
| B13M | C12M | -0.040 |
| B14F | C12M | 0.032 |
| B15M | C12M | -0.023 |
| B16F | C12M | 0.024 |
| B17F | C12M | 0.053 |
| B18M | C12M | -0.068 |
| B19M | C12M | -0.032 |
| B20F | C12M | -0.031 |
| B21F | C12M | -0.034 |
| B22F | C12M | 0.074 |
| B23F | C12M | 0.001 |
| B24F | C12M | -0.059 |
| B25F | C12M | -0.063 |
| B26F | C12M | 0.006 |
| B27F | C12M | -0.004 |
| B28M | C12M | 0.017 |
| B29F | C12M | 0.083 |
| B30F | C12M | -0.095 |
| B31M | C12M | -0.102 |
| B32M | C12M | 0.124 |
| B33M | C12M | 0.012 |
| B34M | C12M | -0.022 |
| B35M | C12M | 0.093 |
| B36M | C12M | -0.070 |
| B37M | C12M | -0.067 |
| C1M | C12M | -0.016 |
| C2M | C12M | 0.320 |
| C3M | C12M | -0.060 |
| C4M | C12M | -0.015 |
| C5M | C12M | 0.008 |
| C6M | C12M | -0.047 |
| C7M | C12M | -0.104 |
| C8M | C12M | -0.105 |
| C9M | C12M | 0.131 |
| C10M | C12M | -0.079 |
| C11M | C12M | -0.089 |
| B1 | C13M | -0.021 |
| B2 | C13M | -0.100 |
| B3 | C13M | -0.078 |
| B4 | C13M | -0.082 |
| B5 | C13M | -0.041 |
| B6 | C13M | 0.079 |
| B7 | C13M | 0.059 |
| B8F | C13M | 0.099 |
| B9F | C13M | 0.220 |
| B10F | C13M | 0.137 |
| B11F | C13M | 0.135 |
| B12F | C13M | -0.093 |
| B13M | C13M | -0.086 |
| B14F | C13M | -0.019 |
| B15M | C13M | -0.040 |
| B16F | C13M | -0.124 |
| B17F | C13M | -0.014 |
| B18M | C13M | -0.041 |
| B19M | C13M | -0.064 |
| B20F | C13M | 0.007 |
| B21F | C13M | -0.071 |
| B22F | C13M | -0.069 |
| B23F | C13M | -0.022 |
| B24F | C13M | -0.059 |
| B25F | C13M | -0.028 |
| B26F | C13M | 0.015 |
| B27F | C13M | -0.055 |
| B28M | C13M | -0.064 |
| B29F | C13M | 0.069 |
| B30F | C13M | -0.111 |
| B31M | C13M | 0.030 |
| B32M | C13M | 0.081 |
| B33M | C13M | -0.053 |
| B34M | C13M | -0.091 |
| B35M | C13M | 0.029 |
| B36M | C13M | 0.053 |
| B37M | C13M | 0.082 |
| C1M | C13M | 0.011 |
| C2M | C13M | -0.002 |
| C3M | C13M | -0.083 |
| C4M | C13M | -0.072 |
| C5M | C13M | -0.039 |
| C6M | C13M | 0.163 |
| C7M | C13M | -0.089 |
| C8M | C13M | -0.021 |
| C9M | C13M | -0.029 |
| C10M | C13M | 0.188 |
| C11M | C13M | 0.005 |
| C12M | C13M | 0.261 |
| B1 | C14M | 0.113 |
| B2 | C14M | -0.014 |
| B3 | C14M | -0.122 |
| B4 | C14M | 0.009 |
| B5 | C14M | -0.001 |
| B6 | C14M | 0.044 |
| B7 | C14M | 0.061 |
| B8F | C14M | -0.085 |
| B9F | C14M | -0.035 |
| B10F | C14M | 0.042 |
| B11F | C14M | -0.038 |
| B12F | C14M | -0.063 |
| B13M | C14M | 0.100 |
| B14F | C14M | -0.020 |
| B15M | C14M | -0.087 |
| B16F | C14M | -0.024 |
| B17F | C14M | -0.047 |
| B18M | C14M | -0.080 |
| B19M | C14M | 0.181 |
| B20F | C14M | -0.039 |
| B21F | C14M | 0.015 |
| B22F | C14M | -0.003 |
| B23F | C14M | -0.009 |
| B24F | C14M | -0.077 |
| B25F | C14M | 0.007 |
| B26F | C14M | 0.044 |
| B27F | C14M | -0.039 |
| B28M | C14M | -0.015 |
| B29F | C14M | -0.081 |
| B30F | C14M | -0.071 |
| B31M | C14M | 0.121 |
| B32M | C14M | 0.026 |
| B33M | C14M | -0.111 |
| B34M | C14M | -0.050 |
| B35M | C14M | -0.084 |
| B36M | C14M | 0.014 |
| B37M | C14M | 0.059 |
| C1M | C14M | -0.007 |
| C2M | C14M | -0.044 |
| C3M | C14M | 0.074 |
| C4M | C14M | 0.155 |
| C5M | C14M | 0.082 |
| C6M | C14M | -0.029 |
| C7M | C14M | 0.035 |
| C8M | C14M | 0.011 |
| C9M | C14M | -0.029 |
| C10M | C14M | 0.003 |
| C11M | C14M | -0.047 |
| C12M | C14M | -0.029 |
| C13M | C14M | -0.023 |
| B1 | C15M | -0.004 |
| B2 | C15M | -0.019 |
| B3 | C15M | -0.095 |
| B4 | C15M | -0.031 |
| B5 | C15M | 0.065 |
| B6 | C15M | -0.009 |
| B7 | C15M | 0.033 |
| B8F | C15M | -0.012 |
| B9F | C15M | -0.025 |
| B10F | C15M | -0.020 |
| B11F | C15M | 0.023 |
| B12F | C15M | -0.040 |
| B13M | C15M | 0.004 |
| B14F | C15M | -0.035 |
| B15M | C15M | 0.022 |
| B16F | C15M | -0.037 |
| B17F | C15M | 0.057 |
| B18M | C15M | -0.049 |
| B19M | C15M | 0.035 |
| B20F | C15M | -0.019 |
| B21F | C15M | 0.064 |
| B22F | C15M | -0.052 |
| B23F | C15M | -0.037 |
| B24F | C15M | -0.018 |
| B25F | C15M | -0.063 |
| B26F | C15M | -0.035 |
| B27F | C15M | -0.036 |
| B28M | C15M | -0.006 |
| B29F | C15M | 0.001 |
| B30F | C15M | -0.023 |
| B31M | C15M | -0.002 |
| B32M | C15M | -0.007 |
| B33M | C15M | 0.004 |
| B34M | C15M | -0.026 |
| B35M | C15M | -0.038 |
| B36M | C15M | -0.048 |
| B37M | C15M | -0.015 |
| C1M | C15M | 0.009 |
| C2M | C15M | 0.073 |
| C3M | C15M | 0.016 |
| C4M | C15M | 0.010 |
| C5M | C15M | -0.052 |
| C6M | C15M | -0.078 |
| C7M | C15M | -0.033 |
| C8M | C15M | 0.031 |
| C9M | C15M | 0.075 |
| C10M | C15M | -0.012 |
| C11M | C15M | -0.042 |
| C12M | C15M | 0.169 |
| C13M | C15M | -0.024 |
| C14M | C15M | 0.054 |
| B1 | C16M | -0.045 |
| B2 | C16M | 0.020 |
| B3 | C16M | -0.085 |
| B4 | C16M | -0.048 |
| B5 | C16M | 0.010 |
| B6 | C16M | -0.036 |
| B7 | C16M | -0.012 |
| B8F | C16M | -0.036 |
| B9F | C16M | 0.024 |
| B10F | C16M | 0.034 |
| B11F | C16M | -0.047 |
| B12F | C16M | -0.012 |
| B13M | C16M | -0.010 |
| B14F | C16M | 0.027 |
| B15M | C16M | 0.001 |
| B16F | C16M | 0.061 |
| B17F | C16M | 0.142 |
| B18M | C16M | 0.043 |
| B19M | C16M | 0.016 |
| B20F | C16M | -0.031 |
| B21F | C16M | 0.064 |
| B22F | C16M | -0.015 |
| B23F | C16M | -0.031 |
| B24F | C16M | 0.102 |
| B25F | C16M | -0.014 |
| B26F | C16M | 0.094 |
| B27F | C16M | 0.017 |
| B28M | C16M | 0.043 |
| B29F | C16M | -0.076 |
| B30F | C16M | 0.041 |
| B31M | C16M | -0.057 |
| B32M | C16M | 0.033 |
| B33M | C16M | 0.022 |
| B34M | C16M | -0.103 |
| B35M | C16M | -0.018 |
| B36M | C16M | -0.054 |
| B37M | C16M | 0.040 |
| C1M | C16M | 0.042 |
| C2M | C16M | -0.057 |
| C3M | C16M | 0.013 |
| C4M | C16M | 0.149 |
| C5M | C16M | -0.038 |
| C6M | C16M | -0.021 |
| C7M | C16M | -0.019 |
| C8M | C16M | 0.038 |
| C9M | C16M | -0.058 |
| C10M | C16M | -0.103 |
| C11M | C16M | -0.043 |
| C12M | C16M | 0.053 |
| C13M | C16M | 0.032 |
| C14M | C16M | 0.062 |
| C15M | C16M | 0.026 |
| B1 | C17M | 0.007 |
| B2 | C17M | -0.065 |
| B3 | C17M | -0.108 |
| B4 | C17M | -0.100 |
| B5 | C17M | 0.010 |
| B6 | C17M | 0.018 |
| B7 | C17M | 0.017 |
| B8F | C17M | -0.007 |
| B9F | C17M | 0.007 |
| B10F | C17M | -0.045 |
| B11F | C17M | -0.008 |
| B12F | C17M | 0.008 |
| B13M | C17M | -0.005 |
| B14F | C17M | -0.078 |
| B15M | C17M | 0.045 |
| B16F | C17M | 0.007 |
| B17F | C17M | 0.087 |
| B18M | C17M | 0.027 |
| B19M | C17M | 0.024 |
| B20F | C17M | -0.053 |
| B21F | C17M | 0.003 |
| B22F | C17M | -0.079 |
| B23F | C17M | -0.050 |
| B24F | C17M | -0.040 |
| B25F | C17M | -0.116 |
| B26F | C17M | -0.055 |
| B27F | C17M | -0.011 |
| B28M | C17M | -0.011 |
| B29F | C17M | -0.034 |
| B30F | C17M | -0.021 |
| B31M | C17M | 0.052 |
| B32M | C17M | -0.055 |
| B33M | C17M | -0.006 |
| B34M | C17M | 0.038 |
| B35M | C17M | -0.035 |
| B36M | C17M | -0.057 |
| B37M | C17M | -0.034 |
| C1M | C17M | 0.007 |
| C2M | C17M | 0.028 |
| C3M | C17M | 0.049 |
| C4M | C17M | 0.013 |
| C5M | C17M | -0.059 |
| C6M | C17M | -0.126 |
| C7M | C17M | -0.031 |
| C8M | C17M | 0.009 |
| C9M | C17M | 0.054 |
| C10M | C17M | 0.004 |
| C11M | C17M | 0.014 |
| C12M | C17M | 0.031 |
| C13M | C17M | -0.039 |
| C14M | C17M | 0.006 |
| C15M | C17M | 0.178 |
| C16M | C17M | -0.033 |
| B1 | C18M | -0.102 |
| B2 | C18M | 0.012 |
| B3 | C18M | -0.110 |
| B4 | C18M | 0.041 |
| B5 | C18M | 0.010 |
| B6 | C18M | -0.074 |
| B7 | C18M | -0.062 |
| B8F | C18M | -0.050 |
| B9F | C18M | -0.045 |
| B10F | C18M | -0.113 |
| B11F | C18M | -0.065 |
| B12F | C18M | -0.015 |
| B13M | C18M | 0.066 |
| B14F | C18M | 0.009 |
| B15M | C18M | -0.126 |
| B16F | C18M | -0.088 |
| B17F | C18M | -0.072 |
| B18M | C18M | 0.008 |
| B19M | C18M | -0.036 |
| B20F | C18M | 0.013 |
| B21F | C18M | 0.042 |
| B22F | C18M | -0.092 |
| B23F | C18M | -0.097 |
| B24F | C18M | 0.051 |
| B25F | C18M | 0.188 |
| B26F | C18M | -0.065 |
| B27F | C18M | -0.057 |
| B28M | C18M | -0.055 |
| B29F | C18M | -0.066 |
| B30F | C18M | 0.039 |
| B31M | C18M | 0.021 |
| B32M | C18M | -0.112 |
| B33M | C18M | -0.143 |
| B34M | C18M | -0.018 |
| B35M | C18M | -0.035 |
| B36M | C18M | -0.086 |
| B37M | C18M | 0.037 |
| C1M | C18M | 0.004 |
| C2M | C18M | -0.036 |
| C3M | C18M | -0.079 |
| C4M | C18M | -0.010 |
| C5M | C18M | 0.020 |
| C6M | C18M | -0.043 |
| C7M | C18M | -0.008 |
| C8M | C18M | -0.023 |
| C9M | C18M | 0.018 |
| C10M | C18M | 0.127 |
| C11M | C18M | -0.092 |
| C12M | C18M | -0.041 |
| C13M | C18M | 0.312 |
| C14M | C18M | 0.112 |
| C15M | C18M | 0.056 |
| C16M | C18M | -0.023 |
| C17M | C18M | 0.030 |
| B1 | C19M | 0.007 |
| B2 | C19M | 0.059 |
| B3 | C19M | -0.084 |
| B4 | C19M | -0.082 |
| B5 | C19M | -0.014 |
| B6 | C19M | -0.038 |
| B7 | C19M | -0.032 |
| B8F | C19M | 0.013 |
| B9F | C19M | -0.021 |
| B10F | C19M | -0.010 |
| B11F | C19M | -0.042 |
| B12F | C19M | 0.006 |
| B13M | C19M | 0.132 |
| B14F | C19M | -0.063 |
| B15M | C19M | -0.053 |
| B16F | C19M | -0.005 |
| B17F | C19M | 0.015 |
| B18M | C19M | 0.065 |
| B19M | C19M | 0.018 |
| B20F | C19M | -0.020 |
| B21F | C19M | -0.031 |
| B22F | C19M | 0.000 |
| B23F | C19M | -0.040 |
| B24F | C19M | 0.063 |
| B25F | C19M | -0.046 |
| B26F | C19M | -0.016 |
| B27F | C19M | -0.073 |
| B28M | C19M | -0.060 |
| B29F | C19M | 0.002 |
| B30F | C19M | -0.038 |
| B31M | C19M | 0.086 |
| B32M | C19M | 0.030 |
| B33M | C19M | 0.020 |
| B34M | C19M | -0.003 |
| B35M | C19M | -0.003 |
| B36M | C19M | 0.042 |
| B37M | C19M | 0.044 |
| C1M | C19M | 0.089 |
| C2M | C19M | 0.024 |
| C3M | C19M | 0.164 |
| C4M | C19M | 0.067 |
| C5M | C19M | -0.031 |
| C6M | C19M | 0.013 |
| C7M | C19M | 0.097 |
| C8M | C19M | -0.001 |
| C9M | C19M | -0.010 |
| C10M | C19M | 0.025 |
| C11M | C19M | 0.000 |
| C12M | C19M | -0.126 |
| C13M | C19M | -0.054 |
| C14M | C19M | 0.039 |
| C15M | C19M | 0.017 |
| C16M | C19M | 0.019 |
| C17M | C19M | 0.085 |
| C18M | C19M | -0.048 |
| B1 | C20M | -0.067 |
| B2 | C20M | 0.031 |
| B3 | C20M | -0.015 |
| B4 | C20M | 0.012 |
| B5 | C20M | -0.034 |
| B6 | C20M | 0.006 |
| B7 | C20M | 0.003 |
| B8F | C20M | 0.010 |
| B9F | C20M | -0.056 |
| B10F | C20M | -0.051 |
| B11F | C20M | 0.011 |
| B12F | C20M | -0.017 |
| B13M | C20M | 0.014 |
| B14F | C20M | 0.064 |
| B15M | C20M | -0.018 |
| B16F | C20M | -0.074 |
| B17F | C20M | -0.014 |
| B18M | C20M | -0.058 |
| B19M | C20M | -0.093 |
| B20F | C20M | -0.037 |
| B21F | C20M | -0.014 |
| B22F | C20M | 0.008 |
| B23F | C20M | 0.007 |
| B24F | C20M | -0.018 |
| B25F | C20M | -0.010 |
| B26F | C20M | -0.070 |
| B27F | C20M | 0.034 |
| B28M | C20M | -0.069 |
| B29F | C20M | -0.019 |
| B30F | C20M | -0.050 |
| B31M | C20M | -0.024 |
| B32M | C20M | -0.086 |
| B33M | C20M | -0.038 |
| B34M | C20M | 0.032 |
| B35M | C20M | -0.052 |
| B36M | C20M | 0.055 |
| B37M | C20M | -0.025 |
| C1M | C20M | -0.010 |
| C2M | C20M | 0.185 |
| C3M | C20M | 0.045 |
| C4M | C20M | 0.012 |
| C5M | C20M | 0.093 |
| C6M | C20M | 0.012 |
| C7M | C20M | 0.074 |
| C8M | C20M | -0.052 |
| C9M | C20M | 0.142 |
| C10M | C20M | 0.002 |
| C11M | C20M | 0.080 |
| C12M | C20M | 0.061 |
| C13M | C20M | -0.059 |
| C14M | C20M | -0.054 |
| C15M | C20M | -0.049 |
| C16M | C20M | -0.081 |
| C17M | C20M | -0.029 |
| C18M | C20M | -0.036 |
| C19M | C20M | 0.061 |
| B1 | C21M | -0.051 |
| B2 | C21M | -0.028 |
| B3 | C21M | -0.007 |
| B4 | C21M | 0.007 |
| B5 | C21M | -0.037 |
| B6 | C21M | -0.046 |
| B7 | C21M | 0.019 |
| B8F | C21M | -0.060 |
| B9F | C21M | -0.069 |
| B10F | C21M | -0.115 |
| B11F | C21M | -0.047 |
| B12F | C21M | 0.015 |
| B13M | C21M | 0.084 |
| B14F | C21M | 0.125 |
| B15M | C21M | -0.064 |
| B16F | C21M | -0.010 |
| B17F | C21M | 0.150 |
| B18M | C21M | -0.057 |
| B19M | C21M | -0.045 |
| B20F | C21M | 0.072 |
| B21F | C21M | 0.000 |
| B22F | C21M | -0.075 |
| B23F | C21M | -0.004 |
| B24F | C21M | -0.085 |
| B25F | C21M | -0.099 |
| B26F | C21M | 0.044 |
| B27F | C21M | 0.076 |
| B28M | C21M | 0.335 |
| B29F | C21M | -0.064 |
| B30F | C21M | -0.057 |
| B31M | C21M | -0.016 |
| B32M | C21M | -0.113 |
| B33M | C21M | -0.079 |
| B34M | C21M | 0.021 |
| B35M | C21M | -0.034 |
| B36M | C21M | -0.065 |
| B37M | C21M | -0.027 |
| C1M | C21M | -0.073 |
| C2M | C21M | -0.037 |
| C3M | C21M | 0.001 |
| C4M | C21M | 0.001 |
| C5M | C21M | -0.089 |
| C6M | C21M | 0.038 |
| C7M | C21M | 0.037 |
| C8M | C21M | 0.234 |
| C9M | C21M | -0.043 |
| C10M | C21M | -0.023 |
| C11M | C21M | -0.082 |
| C12M | C21M | 0.002 |
| C13M | C21M | -0.081 |
| C14M | C21M | 0.054 |
| C15M | C21M | 0.031 |
| C16M | C21M | -0.024 |
| C17M | C21M | 0.065 |
| C18M | C21M | 0.033 |
| C19M | C21M | 0.026 |
| C20M | C21M | 0.025 |
| B1 | C22M | 0.004 |
| B2 | C22M | -0.009 |
| B3 | C22M | -0.131 |
| B4 | C22M | -0.012 |
| B5 | C22M | -0.044 |
| B6 | C22M | -0.063 |
| B7 | C22M | -0.038 |
| B8F | C22M | 0.020 |
| B9F | C22M | -0.007 |
| B10F | C22M | -0.130 |
| B11F | C22M | 0.017 |
| B12F | C22M | -0.104 |
| B13M | C22M | 0.090 |
| B14F | C22M | -0.044 |
| B15M | C22M | -0.073 |
| B16F | C22M | -0.071 |
| B17F | C22M | -0.075 |
| B18M | C22M | 0.076 |
| B19M | C22M | -0.076 |
| B20F | C22M | -0.076 |
| B21F | C22M | -0.002 |
| B22F | C22M | -0.033 |
| B23F | C22M | -0.027 |
| B24F | C22M | 0.042 |
| B25F | C22M | 0.042 |
| B26F | C22M | -0.030 |
| B27F | C22M | 0.016 |
| B28M | C22M | -0.041 |
| B29F | C22M | 0.021 |
| B30F | C22M | -0.048 |
| B31M | C22M | 0.024 |
| B32M | C22M | -0.099 |
| B33M | C22M | -0.127 |
| B34M | C22M | -0.094 |
| B35M | C22M | -0.075 |
| B36M | C22M | -0.036 |
| B37M | C22M | 0.066 |
| C1M | C22M | 0.257 |
| C2M | C22M | -0.019 |
| C3M | C22M | 0.014 |
| C4M | C22M | 0.018 |
| C5M | C22M | 0.063 |
| C6M | C22M | 0.001 |
| C7M | C22M | -0.011 |
| C8M | C22M | -0.070 |
| C9M | C22M | -0.027 |
| C10M | C22M | 0.029 |
| C11M | C22M | 0.024 |
| C12M | C22M | -0.059 |
| C13M | C22M | 0.003 |
| C14M | C22M | 0.012 |
| C15M | C22M | -0.008 |
| C16M | C22M | -0.070 |
| C17M | C22M | 0.007 |
| C18M | C22M | 0.061 |
| C19M | C22M | 0.093 |
| C20M | C22M | 0.003 |
| C21M | C22M | -0.059 |
| B1 | C23M | -0.020 |
| B2 | C23M | -0.033 |
| B3 | C23M | -0.095 |
| B4 | C23M | -0.050 |
| B5 | C23M | -0.019 |
| B6 | C23M | 0.024 |
| B7 | C23M | -0.005 |
| B8F | C23M | 0.048 |
| B9F | C23M | 0.000 |
| B10F | C23M | -0.121 |
| B11F | C23M | -0.107 |
| B12F | C23M | 0.060 |
| B13M | C23M | 0.065 |
| B14F | C23M | -0.054 |
| B15M | C23M | -0.048 |
| B16F | C23M | -0.076 |
| B17F | C23M | -0.051 |
| B18M | C23M | -0.040 |
| B19M | C23M | 0.007 |
| B20F | C23M | 0.023 |
| B21F | C23M | 0.018 |
| B22F | C23M | -0.024 |
| B23F | C23M | -0.086 |
| B24F | C23M | -0.091 |
| B25F | C23M | -0.003 |
| B26F | C23M | -0.079 |
| B27F | C23M | -0.024 |
| B28M | C23M | -0.077 |
| B29F | C23M | 0.016 |
| B30F | C23M | -0.039 |
| B31M | C23M | 0.082 |
| B32M | C23M | -0.067 |
| B33M | C23M | -0.081 |
| B34M | C23M | 0.000 |
| B35M | C23M | 0.024 |
| B36M | C23M | -0.001 |
| B37M | C23M | -0.049 |
| C1M | C23M | -0.058 |
| C2M | C23M | 0.020 |
| C3M | C23M | 0.009 |
| C4M | C23M | -0.011 |
| C5M | C23M | -0.054 |
| C6M | C23M | -0.077 |
| C7M | C23M | 0.062 |
| C8M | C23M | 0.005 |
| C9M | C23M | 0.034 |
| C10M | C23M | -0.007 |
| C11M | C23M | -0.005 |
| C12M | C23M | 0.010 |
| C13M | C23M | 0.032 |
| C14M | C23M | 0.011 |
| C15M | C23M | 0.076 |
| C16M | C23M | -0.062 |
| C17M | C23M | 0.082 |
| C18M | C23M | 0.414 |
| C19M | C23M | 0.036 |
| C20M | C23M | -0.014 |
| C21M | C23M | 0.023 |
| C22M | C23M | 0.036 |
| B1 | C24M | -0.009 |
| B2 | C24M | -0.037 |
| B3 | C24M | -0.052 |
| B4 | C24M | -0.080 |
| B5 | C24M | 0.049 |
| B6 | C24M | -0.055 |
| B7 | C24M | -0.038 |
| B8F | C24M | -0.060 |
| B9F | C24M | 0.065 |
| B10F | C24M | -0.018 |
| B11F | C24M | -0.114 |
| B12F | C24M | 0.042 |
| B13M | C24M | -0.033 |
| B14F | C24M | 0.036 |
| B15M | C24M | -0.069 |
| B16F | C24M | -0.056 |
| B17F | C24M | -0.030 |
| B18M | C24M | 0.035 |
| B19M | C24M | -0.008 |
| B20F | C24M | 0.210 |
| B21F | C24M | -0.005 |
| B22F | C24M | 0.020 |
| B23F | C24M | -0.059 |
| B24F | C24M | 0.002 |
| B25F | C24M | 0.006 |
| B26F | C24M | -0.050 |
| B27F | C24M | -0.033 |
| B28M | C24M | -0.061 |
| B29F | C24M | -0.015 |
| B30F | C24M | -0.072 |
| B31M | C24M | 0.013 |
| B32M | C24M | -0.014 |
| B33M | C24M | -0.010 |
| B34M | C24M | -0.022 |
| B35M | C24M | -0.042 |
| B36M | C24M | 0.041 |
| B37M | C24M | -0.051 |
| C1M | C24M | -0.050 |
| C2M | C24M | 0.028 |
| C3M | C24M | -0.075 |
| C4M | C24M | -0.042 |
| C5M | C24M | 0.144 |
| C6M | C24M | 0.192 |
| C7M | C24M | 0.078 |
| C8M | C24M | 0.114 |
| C9M | C24M | -0.024 |
| C10M | C24M | 0.056 |
| C11M | C24M | -0.064 |
| C12M | C24M | -0.040 |
| C13M | C24M | -0.017 |
| C14M | C24M | 0.026 |
| C15M | C24M | -0.017 |
| C16M | C24M | 0.008 |
| C17M | C24M | -0.061 |
| C18M | C24M | 0.012 |
| C19M | C24M | -0.032 |
| C20M | C24M | 0.025 |
| C21M | C24M | 0.096 |
| C22M | C24M | -0.032 |
| C23M | C24M | 0.023 |
| B1 | C25M | -0.017 |
| B2 | C25M | -0.046 |
| B3 | C25M | -0.090 |
| B4 | C25M | -0.067 |
| B5 | C25M | 0.081 |
| B6 | C25M | 0.042 |
| B7 | C25M | 0.017 |
| B8F | C25M | -0.023 |
| B9F | C25M | -0.014 |
| B10F | C25M | -0.076 |
| B11F | C25M | 0.026 |
| B12F | C25M | 0.033 |
| B13M | C25M | 0.019 |
| B14F | C25M | 0.010 |
| B15M | C25M | -0.043 |
| B16F | C25M | -0.019 |
| B17F | C25M | 0.004 |
| B18M | C25M | 0.017 |
| B19M | C25M | 0.095 |
| B20F | C25M | 0.067 |
| B21F | C25M | -0.017 |
| B22F | C25M | 0.070 |
| B23F | C25M | 0.062 |
| B24F | C25M | 0.015 |
| B25F | C25M | -0.071 |
| B26F | C25M | -0.017 |
| B27F | C25M | -0.015 |
| B28M | C25M | 0.011 |
| B29F | C25M | -0.071 |
| B30F | C25M | -0.074 |
| B31M | C25M | 0.007 |
| B32M | C25M | 0.007 |
| B33M | C25M | -0.050 |
| B34M | C25M | -0.069 |
| B35M | C25M | -0.075 |
| B36M | C25M | 0.160 |
| B37M | C25M | -0.010 |
| C1M | C25M | 0.029 |
| C2M | C25M | 0.015 |
| C3M | C25M | 0.066 |
| C4M | C25M | 0.014 |
| C5M | C25M | 0.072 |
| C6M | C25M | 0.016 |
| C7M | C25M | -0.026 |
| C8M | C25M | -0.085 |
| C9M | C25M | -0.001 |
| C10M | C25M | 0.050 |
| C11M | C25M | -0.010 |
| C12M | C25M | -0.020 |
| C13M | C25M | -0.053 |
| C14M | C25M | 0.037 |
| C15M | C25M | 0.000 |
| C16M | C25M | 0.010 |
| C17M | C25M | 0.001 |
| C18M | C25M | -0.039 |
| C19M | C25M | 0.043 |
| C20M | C25M | 0.090 |
| C21M | C25M | -0.024 |
| C22M | C25M | 0.037 |
| C23M | C25M | -0.002 |
| C24M | C25M | 0.089 |
| B1 | C26M | -0.005 |
| B2 | C26M | 0.070 |
| B3 | C26M | -0.050 |
| B4 | C26M | -0.039 |
| B5 | C26M | 0.044 |
| B6 | C26M | -0.093 |
| B7 | C26M | -0.071 |
| B8F | C26M | -0.074 |
| B9F | C26M | -0.008 |
| B10F | C26M | -0.064 |
| B11F | C26M | -0.065 |
| B12F | C26M | -0.026 |
| B13M | C26M | 0.065 |
| B14F | C26M | -0.055 |
| B15M | C26M | 0.128 |
| B16F | C26M | 0.085 |
| B17F | C26M | -0.056 |
| B18M | C26M | 0.000 |
| B19M | C26M | 0.054 |
| B20F | C26M | 0.042 |
| B21F | C26M | -0.026 |
| B22F | C26M | 0.013 |
| B23F | C26M | -0.036 |
| B24F | C26M | -0.065 |
| B25F | C26M | -0.075 |
| B26F | C26M | 0.021 |
| B27F | C26M | -0.063 |
| B28M | C26M | 0.051 |
| B29F | C26M | -0.078 |
| B30F | C26M | -0.009 |
| B31M | C26M | 0.030 |
| B32M | C26M | -0.043 |
| B33M | C26M | 0.016 |
| B34M | C26M | 0.120 |
| B35M | C26M | -0.080 |
| B36M | C26M | 0.003 |
| B37M | C26M | -0.075 |
| C1M | C26M | 0.006 |
| C2M | C26M | 0.053 |
| C3M | C26M | 0.001 |
| C4M | C26M | -0.064 |
| C5M | C26M | -0.052 |
| C6M | C26M | -0.031 |
| C7M | C26M | 0.058 |
| C8M | C26M | 0.080 |
| C9M | C26M | 0.046 |
| C10M | C26M | -0.065 |
| C11M | C26M | 0.046 |
| C12M | C26M | -0.005 |
| C13M | C26M | -0.048 |
| C14M | C26M | -0.017 |
| C15M | C26M | 0.035 |
| C16M | C26M | -0.009 |
| C17M | C26M | -0.009 |
| C18M | C26M | 0.032 |
| C19M | C26M | 0.025 |
| C20M | C26M | -0.014 |
| C21M | C26M | 0.058 |
| C22M | C26M | 0.012 |
| C23M | C26M | 0.092 |
| C24M | C26M | 0.054 |
| C25M | C26M | 0.015 |
| B1 | C27M | 0.022 |
| B2 | C27M | 0.029 |
| B3 | C27M | -0.053 |
| B4 | C27M | -0.049 |
| B5 | C27M | 0.022 |
| B6 | C27M | -0.052 |
| B7 | C27M | -0.024 |
| B8F | C27M | -0.050 |
| B9F | C27M | -0.036 |
| B10F | C27M | -0.088 |
| B11F | C27M | 0.010 |
| B12F | C27M | -0.071 |
| B13M | C27M | -0.012 |
| B14F | C27M | 0.029 |
| B15M | C27M | 0.126 |
| B16F | C27M | 0.054 |
| B17F | C27M | -0.061 |
| B18M | C27M | 0.004 |
| B19M | C27M | 0.056 |
| B20F | C27M | -0.009 |
| B21F | C27M | 0.009 |
| B22F | C27M | 0.027 |
| B23F | C27M | -0.050 |
| B24F | C27M | -0.114 |
| B25F | C27M | -0.107 |
| B26F | C27M | 0.039 |
| B27F | C27M | -0.025 |
| B28M | C27M | -0.009 |
| B29F | C27M | -0.066 |
| B30F | C27M | -0.027 |
| B31M | C27M | -0.009 |
| B32M | C27M | -0.061 |
| B33M | C27M | -0.018 |
| B34M | C27M | 0.050 |
| B35M | C27M | -0.046 |
| B36M | C27M | 0.044 |
| B37M | C27M | 0.068 |
| C1M | C27M | 0.046 |
| C2M | C27M | -0.003 |
| C3M | C27M | -0.002 |
| C4M | C27M | -0.007 |
| C5M | C27M | -0.013 |
| C6M | C27M | -0.100 |
| C7M | C27M | 0.073 |
| C8M | C27M | 0.158 |
| C9M | C27M | 0.043 |
| C10M | C27M | 0.000 |
| C11M | C27M | 0.026 |
| C12M | C27M | -0.098 |
| C13M | C27M | -0.046 |
| C14M | C27M | 0.092 |
| C15M | C27M | 0.001 |
| C16M | C27M | 0.011 |
| C17M | C27M | -0.059 |
| C18M | C27M | -0.012 |
| C19M | C27M | -0.043 |
| C20M | C27M | 0.073 |
| C21M | C27M | -0.017 |
| C22M | C27M | 0.013 |
| C23M | C27M | -0.016 |
| C24M | C27M | -0.026 |
| C25M | C27M | -0.006 |
| C26M | C27M | 0.190 |
| B1 | C28M | -0.077 |
| B2 | C28M | 0.023 |
| B3 | C28M | 0.087 |
| B4 | C28M | -0.048 |
| B5 | C28M | -0.060 |
| B6 | C28M | -0.034 |
| B7 | C28M | 0.002 |
| B8F | C28M | -0.055 |
| B9F | C28M | -0.040 |
| B10F | C28M | 0.020 |
| B11F | C28M | -0.078 |
| B12F | C28M | 0.097 |
| B13M | C28M | 0.029 |
| B14F | C28M | 0.065 |
| B15M | C28M | 0.001 |
| B16F | C28M | 0.155 |
| B17F | C28M | -0.050 |
| B18M | C28M | 0.172 |
| B19M | C28M | -0.027 |
| B20F | C28M | 0.012 |
| B21F | C28M | 0.012 |
| B22F | C28M | -0.007 |
| B23F | C28M | -0.056 |
| B24F | C28M | -0.029 |
| B25F | C28M | -0.100 |
| B26F | C28M | 0.102 |
| B27F | C28M | 0.146 |
| B28M | C28M | -0.069 |
| B29F | C28M | -0.097 |
| B30F | C28M | 0.041 |
| B31M | C28M | -0.003 |
| B32M | C28M | 0.032 |
| B33M | C28M | -0.083 |
| B34M | C28M | -0.004 |
| B35M | C28M | 0.021 |
| B36M | C28M | -0.094 |
| B37M | C28M | -0.024 |
| C1M | C28M | 0.068 |
| C2M | C28M | -0.037 |
| C3M | C28M | -0.025 |
| C4M | C28M | 0.018 |
| C5M | C28M | -0.057 |
| C6M | C28M | -0.042 |
| C7M | C28M | -0.023 |
| C8M | C28M | 0.099 |
| C9M | C28M | -0.045 |
| C10M | C28M | -0.063 |
| C11M | C28M | -0.048 |
| C12M | C28M | -0.028 |
| C13M | C28M | -0.071 |
| C14M | C28M | -0.016 |
| C15M | C28M | -0.045 |
| C16M | C28M | 0.040 |
| C17M | C28M | 0.078 |
| C18M | C28M | -0.040 |
| C19M | C28M | 0.092 |
| C20M | C28M | -0.081 |
| C21M | C28M | 0.146 |
| C22M | C28M | 0.346 |
| C23M | C28M | -0.063 |
| C24M | C28M | 0.009 |
| C25M | C28M | -0.087 |
| C26M | C28M | -0.029 |
| C27M | C28M | -0.038 |
| B1 | C29M | 0.004 |
| B2 | C29M | 0.016 |
| B3 | C29M | -0.030 |
| B4 | C29M | -0.007 |
| B5 | C29M | 0.008 |
| B6 | C29M | 0.023 |
| B7 | C29M | 0.025 |
| B8F | C29M | -0.012 |
| B9F | C29M | -0.062 |
| B10F | C29M | 0.019 |
| B11F | C29M | -0.024 |
| B12F | C29M | -0.020 |
| B13M | C29M | -0.003 |
| B14F | C29M | -0.028 |
| B15M | C29M | 0.046 |
| B16F | C29M | -0.033 |
| B17F | C29M | -0.027 |
| B18M | C29M | -0.052 |
| B19M | C29M | 0.032 |
| B20F | C29M | -0.006 |
| B21F | C29M | -0.042 |
| B22F | C29M | -0.010 |
| B23F | C29M | -0.059 |
| B24F | C29M | 0.045 |
| B25F | C29M | -0.020 |
| B26F | C29M | -0.033 |
| B27F | C29M | -0.065 |
| B28M | C29M | -0.080 |
| B29F | C29M | -0.049 |
| B30F | C29M | -0.023 |
| B31M | C29M | -0.027 |
| B32M | C29M | 0.016 |
| B33M | C29M | -0.041 |
| B34M | C29M | 0.038 |
| B35M | C29M | -0.019 |
| B36M | C29M | 0.009 |
| B37M | C29M | -0.003 |
| C1M | C29M | 0.199 |
| C2M | C29M | -0.038 |
| C3M | C29M | -0.005 |
| C4M | C29M | -0.011 |
| C5M | C29M | 0.002 |
| C6M | C29M | -0.059 |
| C7M | C29M | 0.006 |
| C8M | C29M | 0.036 |
| C9M | C29M | 0.070 |
| C10M | C29M | -0.028 |
| C11M | C29M | 0.028 |
| C12M | C29M | -0.100 |
| C13M | C29M | -0.068 |
| C14M | C29M | 0.017 |
| C15M | C29M | 0.067 |
| C16M | C29M | 0.035 |
| C17M | C29M | -0.002 |
| C18M | C29M | 0.005 |
| C19M | C29M | 0.029 |
| C20M | C29M | -0.030 |
| C21M | C29M | -0.028 |
| C22M | C29M | 0.093 |
| C23M | C29M | -0.022 |
| C24M | C29M | -0.009 |
| C25M | C29M | -0.007 |
| C26M | C29M | 0.000 |
| C27M | C29M | 0.035 |
| C28M | C29M | 0.001 |
| B1 | C30M | 0.054 |
| B2 | C30M | -0.029 |
| B3 | C30M | -0.022 |
| B4 | C30M | -0.019 |
| B5 | C30M | 0.028 |
| B6 | C30M | 0.017 |
| B7 | C30M | 0.011 |
| B8F | C30M | -0.052 |
| B9F | C30M | -0.068 |
| B10F | C30M | -0.084 |
| B11F | C30M | -0.042 |
| B12F | C30M | 0.093 |
| B13M | C30M | -0.010 |
| B14F | C30M | -0.026 |
| B15M | C30M | 0.033 |
| B16F | C30M | -0.057 |
| B17F | C30M | -0.057 |
| B18M | C30M | -0.040 |
| B19M | C30M | 0.039 |
| B20F | C30M | 0.012 |
| B21F | C30M | -0.028 |
| B22F | C30M | -0.046 |
| B23F | C30M | -0.039 |
| B24F | C30M | -0.035 |
| B25F | C30M | -0.097 |
| B26F | C30M | -0.053 |
| B27F | C30M | -0.040 |
| B28M | C30M | -0.061 |
| B29F | C30M | 0.005 |
| B30F | C30M | -0.072 |
| B31M | C30M | -0.029 |
| B32M | C30M | -0.087 |
| B33M | C30M | -0.031 |
| B34M | C30M | 0.094 |
| B35M | C30M | 0.098 |
| B36M | C30M | 0.007 |
| B37M | C30M | -0.022 |
| C1M | C30M | 0.045 |
| C2M | C30M | -0.046 |
| C3M | C30M | 0.043 |
| C4M | C30M | -0.002 |
| C5M | C30M | -0.016 |
| C6M | C30M | -0.043 |
| C7M | C30M | 0.084 |
| C8M | C30M | 0.093 |
| C9M | C30M | 0.078 |
| C10M | C30M | -0.018 |
| C11M | C30M | 0.096 |
| C12M | C30M | -0.046 |
| C13M | C30M | -0.023 |
| C14M | C30M | 0.000 |
| C15M | C30M | -0.010 |
| C16M | C30M | -0.058 |
| C17M | C30M | 0.122 |
| C18M | C30M | 0.014 |
| C19M | C30M | 0.025 |
| C20M | C30M | 0.013 |
| C21M | C30M | 0.044 |
| C22M | C30M | 0.068 |
| C23M | C30M | 0.030 |
| C24M | C30M | -0.026 |
| C25M | C30M | -0.012 |
| C26M | C30M | 0.017 |
| C27M | C30M | 0.038 |
| C28M | C30M | 0.212 |
| C29M | C30M | 0.059 |
| B1 | B1m | -0.009 |
| B2 | B1m | -0.049 |
| B3 | B1m | -0.104 |
| B4 | B1m | 0.040 |
| B5 | B1m | 0.086 |
| B6 | B1m | 0.027 |
| B7 | B1m | 0.065 |
| B8F | B1m | -0.019 |
| B9F | B1m | -0.016 |
| B10F | B1m | -0.076 |
| B11F | B1m | 0.273 |
| B12F | B1m | 0.057 |
| B13M | B1m | -0.039 |
| B14F | B1m | -0.020 |
| B15M | B1m | -0.019 |
| B16F | B1m | 0.008 |
| B17F | B1m | -0.060 |
| B18M | B1m | -0.048 |
| B19M | B1m | -0.030 |
| B20F | B1m | 0.036 |
| B21F | B1m | 0.010 |
| B22F | B1m | -0.067 |
| B23F | B1m | -0.077 |
| B24F | B1m | -0.073 |
| B25F | B1m | -0.004 |
| B26F | B1m | -0.040 |
| B27F | B1m | -0.036 |
| B28M | B1m | -0.038 |
| B29F | B1m | 0.000 |
| B30F | B1m | -0.037 |
| B31M | B1m | -0.068 |
| B32M | B1m | 0.013 |
| B33M | B1m | -0.035 |
| B34M | B1m | -0.045 |
| B35M | B1m | 0.088 |
| B36M | B1m | -0.095 |
| B37M | B1m | -0.002 |
| C1M | B1m | -0.019 |
| C2M | B1m | -0.042 |
| C3M | B1m | 0.002 |
| C4M | B1m | -0.035 |
| C5M | B1m | -0.057 |
| C6M | B1m | -0.039 |
| C7M | B1m | -0.026 |
| C8M | B1m | -0.030 |
| C9M | B1m | 0.039 |
| C10M | B1m | 0.022 |
| C11M | B1m | -0.011 |
| C12M | B1m | 0.005 |
| C13M | B1m | 0.004 |
| C14M | B1m | -0.048 |
| C15M | B1m | 0.068 |
| C16M | B1m | -0.037 |
| C17M | B1m | 0.158 |
| C18M | B1m | 0.021 |
| C19M | B1m | -0.002 |
| C20M | B1m | -0.050 |
| C21M | B1m | 0.002 |
| C22M | B1m | -0.036 |
| C23M | B1m | 0.020 |
| C24M | B1m | -0.038 |
| C25M | B1m | -0.017 |
| C26M | B1m | -0.029 |
| C27M | B1m | -0.060 |
| C28M | B1m | -0.006 |
| C29M | B1m | 0.007 |
| C30M | B1m | 0.159 |
| B1 | B2m | -0.016 |
| B2 | B2m | 0.004 |
| B3 | B2m | -0.060 |
| B4 | B2m | -0.063 |
| B5 | B2m | 0.028 |
| B6 | B2m | -0.051 |
| B7 | B2m | 0.013 |
| B8F | B2m | 0.013 |
| B9F | B2m | -0.067 |
| B10F | B2m | -0.077 |
| B11F | B2m | -0.016 |
| B12F | B2m | -0.032 |
| B13M | B2m | -0.011 |
| B14F | B2m | -0.014 |
| B15M | B2m | 0.061 |
| B16F | B2m | -0.053 |
| B17F | B2m | -0.065 |
| B18M | B2m | -0.031 |
| B19M | B2m | 0.000 |
| B20F | B2m | -0.039 |
| B21F | B2m | -0.024 |
| B22F | B2m | -0.039 |
| B23F | B2m | -0.025 |
| B24F | B2m | -0.050 |
| B25F | B2m | -0.053 |
| B26F | B2m | -0.020 |
| B27F | B2m | -0.021 |
| B28M | B2m | -0.038 |
| B29F | B2m | -0.052 |
| B30F | B2m | -0.011 |
| B31M | B2m | 0.009 |
| B32M | B2m | -0.029 |
| B33M | B2m | -0.049 |
| B34M | B2m | 0.012 |
| B35M | B2m | -0.041 |
| B36M | B2m | -0.019 |
| B37M | B2m | -0.062 |
| C1M | B2m | 0.016 |
| C2M | B2m | -0.035 |
| C3M | B2m | -0.011 |
| C4M | B2m | -0.015 |
| C5M | B2m | -0.070 |
| C6M | B2m | -0.081 |
| C7M | B2m | 0.018 |
| C8M | B2m | 0.035 |
| C9M | B2m | 0.077 |
| C10M | B2m | -0.025 |
| C11M | B2m | 0.015 |
| C12M | B2m | -0.066 |
| C13M | B2m | -0.062 |
| C14M | B2m | -0.011 |
| C15M | B2m | 0.053 |
| C16M | B2m | 0.034 |
| C17M | B2m | 0.051 |
| C18M | B2m | -0.042 |
| C19M | B2m | -0.004 |
| C20M | B2m | -0.004 |
| C21M | B2m | -0.016 |
| C22M | B2m | -0.054 |
| C23M | B2m | 0.020 |
| C24M | B2m | 0.008 |
| C25M | B2m | 0.010 |
| C26M | B2m | 0.053 |
| C27M | B2m | 0.038 |
| C28M | B2m | -0.008 |
| C29M | B2m | 0.096 |
| C30M | B2m | 0.036 |
| B1m | B2m | 0.026 |
| B1 | B3m | -0.068 |
| B2 | B3m | 0.048 |
| B3 | B3m | 0.067 |
| B4 | B3m | 0.007 |
| B5 | B3m | -0.072 |
| B6 | B3m | -0.010 |
| B7 | B3m | -0.006 |
| B8F | B3m | -0.008 |
| B9F | B3m | 0.007 |
| B10F | B3m | -0.060 |
| B11F | B3m | 0.018 |
| B12F | B3m | 0.033 |
| B13M | B3m | 0.055 |
| B14F | B3m | -0.024 |
| B15M | B3m | 0.022 |
| B16F | B3m | -0.049 |
| B17F | B3m | 0.047 |
| B18M | B3m | -0.008 |
| B19M | B3m | -0.090 |
| B20F | B3m | -0.087 |
| B21F | B3m | -0.022 |
| B22F | B3m | -0.075 |
| B23F | B3m | 0.055 |
| B24F | B3m | -0.031 |
| B25F | B3m | -0.001 |
| B26F | B3m | -0.004 |
| B27F | B3m | -0.019 |
| B28M | B3m | 0.008 |
| B29F | B3m | 0.126 |
| B30F | B3m | -0.027 |
| B31M | B3m | -0.077 |
| B32M | B3m | -0.093 |
| B33M | B3m | 0.021 |
| B34M | B3m | -0.025 |
| B35M | B3m | 0.107 |
| B36M | B3m | -0.081 |
| B37M | B3m | 0.065 |
| C1M | B3m | -0.106 |
| C2M | B3m | -0.077 |
| C3M | B3m | 0.007 |
| C4M | B3m | 0.008 |
| C5M | B3m | -0.103 |
| C6M | B3m | 0.083 |
| C7M | B3m | -0.012 |
| C8M | B3m | -0.001 |
| C9M | B3m | -0.058 |
| C10M | B3m | -0.072 |
| C11M | B3m | -0.020 |
| C12M | B3m | 0.008 |
| C13M | B3m | -0.030 |
| C14M | B3m | -0.041 |
| C15M | B3m | -0.015 |
| C16M | B3m | 0.034 |
| C17M | B3m | 0.003 |
| C18M | B3m | -0.012 |
| C19M | B3m | 0.006 |
| C20M | B3m | -0.002 |
| C21M | B3m | 0.007 |
| C22M | B3m | -0.059 |
| C23M | B3m | -0.001 |
| C24M | B3m | -0.115 |
| C25M | B3m | -0.027 |
| C26M | B3m | -0.060 |
| C27M | B3m | -0.030 |
| C28M | B3m | -0.054 |
| C29M | B3m | -0.057 |
| C30M | B3m | 0.023 |
| B1m | B3m | 0.016 |
| B2m | B3m | -0.046 |
| B1 | B4m | -0.040 |
| B2 | B4m | 0.053 |
| B3 | B4m | -0.011 |
| B4 | B4m | -0.055 |
| B5 | B4m | -0.018 |
| B6 | B4m | -0.045 |
| B7 | B4m | -0.063 |
| B8F | B4m | 0.033 |
| B9F | B4m | -0.047 |
| B10F | B4m | -0.069 |
| B11F | B4m | -0.004 |
| B12F | B4m | -0.018 |
| B13M | B4m | -0.024 |
| B14F | B4m | -0.067 |
| B15M | B4m | 0.157 |
| B16F | B4m | -0.040 |
| B17F | B4m | -0.074 |
| B18M | B4m | -0.051 |
| B19M | B4m | -0.033 |
| B20F | B4m | -0.041 |
| B21F | B4m | -0.025 |
| B22F | B4m | 0.002 |
| B23F | B4m | -0.019 |
| B24F | B4m | -0.029 |
| B25F | B4m | -0.066 |
| B26F | B4m | -0.036 |
| B27F | B4m | -0.013 |
| B28M | B4m | -0.040 |
| B29F | B4m | -0.047 |
| B30F | B4m | -0.021 |
| B31M | B4m | -0.027 |
| B32M | B4m | -0.079 |
| B33M | B4m | 0.003 |
| B34M | B4m | 0.047 |
| B35M | B4m | 0.020 |
| B36M | B4m | 0.002 |
| B37M | B4m | -0.033 |
| C1M | B4m | -0.010 |
| C2M | B4m | 0.015 |
| C3M | B4m | 0.030 |
| C4M | B4m | -0.041 |
| C5M | B4m | -0.021 |
| C6M | B4m | -0.070 |
| C7M | B4m | 0.061 |
| C8M | B4m | 0.011 |
| C9M | B4m | 0.003 |
| C10M | B4m | -0.050 |
| C11M | B4m | 0.042 |
| C12M | B4m | -0.070 |
| C13M | B4m | -0.089 |
| C14M | B4m | -0.079 |
| C15M | B4m | -0.034 |
| C16M | B4m | 0.021 |
| C17M | B4m | -0.070 |
| C18M | B4m | -0.040 |
| C19M | B4m | 0.024 |
| C20M | B4m | -0.009 |
| C21M | B4m | -0.036 |
| C22M | B4m | -0.015 |
| C23M | B4m | 0.011 |
| C24M | B4m | -0.024 |
| C25M | B4m | -0.031 |
| C26M | B4m | 0.036 |
| C27M | B4m | 0.002 |
| C28M | B4m | 0.039 |
| C29M | B4m | 0.017 |
| C30M | B4m | 0.033 |
| B1m | B4m | -0.002 |
| B2m | B4m | 0.127 |
| B3m | B4m | 0.040 |
| B1 | B5m | -0.076 |
| B2 | B5m | -0.065 |
| B3 | B5m | 0.269 |
| B4 | B5m | 0.060 |
| B5 | B5m | 0.022 |
| B6 | B5m | -0.041 |
| B7 | B5m | -0.003 |
| B8F | B5m | -0.019 |
| B9F | B5m | -0.061 |
| B10F | B5m | -0.076 |
| B11F | B5m | 0.023 |
| B12F | B5m | -0.027 |
| B13M | B5m | 0.029 |
| B14F | B5m | -0.047 |
| B15M | B5m | -0.019 |
| B16F | B5m | -0.049 |
| B17F | B5m | -0.052 |
| B18M | B5m | -0.070 |
| B19M | B5m | -0.084 |
| B20F | B5m | -0.032 |
| B21F | B5m | -0.009 |
| B22F | B5m | -0.054 |
| B23F | B5m | -0.071 |
| B24F | B5m | 0.024 |
| B25F | B5m | 0.025 |
| B26F | B5m | -0.070 |
| B27F | B5m | -0.029 |
| B28M | B5m | -0.058 |
| B29F | B5m | 0.091 |
| B30F | B5m | -0.051 |
| B31M | B5m | 0.055 |
| B32M | B5m | -0.042 |
| B33M | B5m | -0.009 |
| B34M | B5m | -0.005 |
| B35M | B5m | 0.001 |
| B36M | B5m | -0.084 |
| B37M | B5m | 0.006 |
| C1M | B5m | -0.026 |
| C2M | B5m | -0.038 |
| C3M | B5m | 0.002 |
| C4M | B5m | -0.071 |
| C5M | B5m | -0.012 |
| C6M | B5m | -0.088 |
| C7M | B5m | -0.010 |
| C8M | B5m | -0.071 |
| C9M | B5m | -0.024 |
| C10M | B5m | 0.001 |
| C11M | B5m | -0.010 |
| C12M | B5m | -0.036 |
| C13M | B5m | -0.063 |
| C14M | B5m | -0.065 |
| C15M | B5m | 0.028 |
| C16M | B5m | -0.081 |
| C17M | B5m | -0.024 |
| C18M | B5m | 0.009 |
| C19M | B5m | -0.013 |
| C20M | B5m | -0.048 |
| C21M | B5m | -0.026 |
| C22M | B5m | 0.021 |
| C23M | B5m | 0.018 |
| C24M | B5m | -0.043 |
| C25M | B5m | -0.064 |
| C26M | B5m | -0.030 |
| C27M | B5m | -0.008 |
| C28M | B5m | -0.086 |
| C29M | B5m | 0.039 |
| C30M | B5m | -0.068 |
| B1m | B5m | -0.006 |
| B2m | B5m | 0.021 |
| B3m | B5m | 0.096 |
| B4m | B5m | 0.062 |
| B1 | B6m | -0.063 |
| B2 | B6m | -0.046 |
| B3 | B6m | -0.044 |
| B4 | B6m | 0.024 |
| B5 | B6m | 0.017 |
| B6 | B6m | -0.053 |
| B7 | B6m | -0.042 |
| B8F | B6m | 0.024 |
| B9F | B6m | -0.067 |
| B10F | B6m | -0.074 |
| B11F | B6m | 0.038 |
| B12F | B6m | -0.014 |
| B13M | B6m | -0.024 |
| B14F | B6m | -0.060 |
| B15M | B6m | 0.037 |
| B16F | B6m | 0.005 |
| B17F | B6m | -0.033 |
| B18M | B6m | -0.103 |
| B19M | B6m | -0.104 |
| B20F | B6m | -0.060 |
| B21F | B6m | -0.034 |
| B22F | B6m | -0.089 |
| B23F | B6m | -0.077 |
| B24F | B6m | 0.002 |
| B25F | B6m | -0.027 |
| B26F | B6m | -0.064 |
| B27F | B6m | 0.013 |
| B28M | B6m | -0.040 |
| B29F | B6m | -0.032 |
| B30F | B6m | 0.004 |
| B31M | B6m | 0.023 |
| B32M | B6m | -0.105 |
| B33M | B6m | -0.057 |
| B34M | B6m | 0.009 |
| B35M | B6m | -0.004 |
| B36M | B6m | -0.089 |
| B37M | B6m | 0.008 |
| C1M | B6m | -0.025 |
| C2M | B6m | -0.050 |
| C3M | B6m | 0.021 |
| C4M | B6m | -0.046 |
| C5M | B6m | 0.027 |
| C6M | B6m | -0.093 |
| C7M | B6m | 0.006 |
| C8M | B6m | -0.057 |
| C9M | B6m | -0.025 |
| C10M | B6m | 0.029 |
| C11M | B6m | 0.056 |
| C12M | B6m | -0.115 |
| C13M | B6m | -0.082 |
| C14M | B6m | -0.038 |
| C15M | B6m | -0.050 |
| C16M | B6m | -0.029 |
| C17M | B6m | 0.016 |
| C18M | B6m | -0.049 |
| C19M | B6m | -0.015 |
| C20M | B6m | -0.036 |
| C21M | B6m | -0.077 |
| C22M | B6m | -0.013 |
| C23M | B6m | -0.039 |
| C24M | B6m | -0.094 |
| C25M | B6m | -0.075 |
| C26M | B6m | -0.085 |
| C27M | B6m | 0.007 |
| C28M | B6m | -0.053 |
| C29M | B6m | 0.065 |
| C30M | B6m | -0.022 |
| B1m | B6m | -0.016 |
| B2m | B6m | 0.119 |
| B3m | B6m | -0.028 |
| B4m | B6m | 0.128 |
| B5m | B6m | 0.146 |
| B1 | B7m | -0.108 |
| B2 | B7m | -0.044 |
| B3 | B7m | -0.010 |
| B4 | B7m | 0.071 |
| B5 | B7m | -0.021 |
| B6 | B7m | 0.136 |
| B7 | B7m | 0.036 |
| B8F | B7m | 0.014 |
| B9F | B7m | -0.015 |
| B10F | B7m | -0.032 |
| B11F | B7m | 0.012 |
| B12F | B7m | -0.069 |
| B13M | B7m | 0.011 |
| B14F | B7m | 0.047 |
| B15M | B7m | -0.030 |
| B16F | B7m | 0.036 |
| B17F | B7m | -0.029 |
| B18M | B7m | -0.061 |
| B19M | B7m | -0.016 |
| B20F | B7m | 0.003 |
| B21F | B7m | 0.130 |
| B22F | B7m | 0.030 |
| B23F | B7m | -0.027 |
| B24F | B7m | -0.047 |
| B25F | B7m | 0.037 |
| B26F | B7m | -0.052 |
| B27F | B7m | 0.080 |
| B28M | B7m | 0.004 |
| B29F | B7m | -0.038 |
| B30F | B7m | 0.011 |
| B31M | B7m | -0.061 |
| B32M | B7m | -0.117 |
| B33M | B7m | -0.019 |
| B34M | B7m | -0.055 |
| B35M | B7m | -0.105 |
| B36M | B7m | -0.025 |
| B37M | B7m | -0.057 |
| C1M | B7m | 0.022 |
| C2M | B7m | 0.044 |
| C3M | B7m | -0.026 |
| C4M | B7m | -0.019 |
| C5M | B7m | 0.003 |
| C6M | B7m | -0.121 |
| C7M | B7m | -0.038 |
| C8M | B7m | 0.016 |
| C9M | B7m | 0.004 |
| C10M | B7m | -0.017 |
| C11M | B7m | 0.043 |
| C12M | B7m | -0.016 |
| C13M | B7m | -0.044 |
| C14M | B7m | -0.003 |
| C15M | B7m | 0.139 |
| C16M | B7m | 0.000 |
| C17M | B7m | 0.042 |
| C18M | B7m | 0.103 |
| C19M | B7m | -0.061 |
| C20M | B7m | 0.017 |
| C21M | B7m | 0.058 |
| C22M | B7m | 0.045 |
| C23M | B7m | 0.026 |
| C24M | B7m | -0.030 |
| C25M | B7m | 0.001 |
| C26M | B7m | -0.004 |
| C27M | B7m | 0.064 |
| C28M | B7m | -0.037 |
| C29M | B7m | 0.048 |
| C30M | B7m | -0.025 |
| B1m | B7m | -0.052 |
| B2m | B7m | -0.038 |
| B3m | B7m | 0.008 |
| B4m | B7m | -0.044 |
| B5m | B7m | 0.095 |
| B6m | B7m | -0.048 |
| B1 | B8m | 0.018 |
| B2 | B8m | 0.298 |
| B3 | B8m | -0.078 |
| B4 | B8m | 0.024 |
| B5 | B8m | 0.025 |
| B6 | B8m | 0.145 |
| B7 | B8m | 0.043 |
| B8F | B8m | -0.047 |
| B9F | B8m | -0.037 |
| B10F | B8m | 0.008 |
| B11F | B8m | 0.060 |
| B12F | B8m | -0.033 |
| B13M | B8m | -0.006 |
| B14F | B8m | 0.027 |
| B15M | B8m | -0.053 |
| B16F | B8m | -0.109 |
| B17F | B8m | -0.098 |
| B18M | B8m | -0.089 |
| B19M | B8m | 0.061 |
| B20F | B8m | -0.032 |
| B21F | B8m | -0.001 |
| B22F | B8m | -0.085 |
| B23F | B8m | -0.017 |
| B24F | B8m | -0.019 |
| B25F | B8m | -0.018 |
| B26F | B8m | 0.067 |
| B27F | B8m | -0.008 |
| B28M | B8m | -0.016 |
| B29F | B8m | -0.082 |
| B30F | B8m | -0.059 |
| B31M | B8m | -0.057 |
| B32M | B8m | -0.061 |
| B33M | B8m | -0.055 |
| B34M | B8m | -0.112 |
| B35M | B8m | -0.105 |
| B36M | B8m | 0.005 |
| B37M | B8m | -0.057 |
| C1M | B8m | -0.063 |
| C2M | B8m | -0.015 |
| C3M | B8m | -0.052 |
| C4M | B8m | -0.037 |
| C5M | B8m | -0.056 |
| C6M | B8m | 0.110 |
| C7M | B8m | -0.055 |
| C8M | B8m | 0.029 |
| C9M | B8m | -0.050 |
| C10M | B8m | -0.008 |
| C11M | B8m | -0.028 |
| C12M | B8m | -0.032 |
| C13M | B8m | -0.001 |
| C14M | B8m | 0.048 |
| C15M | B8m | 0.021 |
| C16M | B8m | -0.017 |
| C17M | B8m | 0.158 |
| C18M | B8m | 0.035 |
| C19M | B8m | -0.056 |
| C20M | B8m | 0.058 |
| C21M | B8m | 0.058 |
| C22M | B8m | -0.027 |
| C23M | B8m | -0.021 |
| C24M | B8m | -0.020 |
| C25M | B8m | 0.045 |
| C26M | B8m | -0.058 |
| C27M | B8m | -0.007 |
| C28M | B8m | -0.068 |
| C29M | B8m | -0.013 |
| C30M | B8m | 0.060 |
| B1m | B8m | 0.044 |
| B2m | B8m | 0.015 |
| B3m | B8m | -0.029 |
| B4m | B8m | -0.083 |
| B5m | B8m | -0.039 |
| B6m | B8m | -0.070 |
| B7m | B8m | 0.049 |
| B1 | B9m | -0.071 |
| B2 | B9m | -0.031 |
| B3 | B9m | -0.002 |
| B4 | B9m | -0.028 |
| B5 | B9m | -0.034 |
| B6 | B9m | -0.056 |
| B7 | B9m | -0.030 |
| B8F | B9m | 0.082 |
| B9F | B9m | 0.012 |
| B10F | B9m | -0.074 |
| B11F | B9m | 0.104 |
| B12F | B9m | -0.040 |
| B13M | B9m | -0.064 |
| B14F | B9m | -0.051 |
| B15M | B9m | 0.052 |
| B16F | B9m | -0.064 |
| B17F | B9m | -0.087 |
| B18M | B9m | 0.062 |
| B19M | B9m | -0.085 |
| B20F | B9m | -0.094 |
| B21F | B9m | -0.067 |
| B22F | B9m | -0.015 |
| B23F | B9m | -0.123 |
| B24F | B9m | -0.012 |
| B25F | B9m | -0.013 |
| B26F | B9m | -0.015 |
| B27F | B9m | 0.001 |
| B28M | B9m | -0.025 |
| B29F | B9m | -0.062 |
| B30F | B9m | -0.071 |
| B31M | B9m | -0.044 |
| B32M | B9m | -0.039 |
| B33M | B9m | 0.007 |
| B34M | B9m | 0.059 |
| B35M | B9m | -0.041 |
| B36M | B9m | -0.037 |
| B37M | B9m | -0.002 |
| C1M | B9m | 0.016 |
| C2M | B9m | 0.069 |
| C3M | B9m | 0.030 |
| C4M | B9m | -0.014 |
| C5M | B9m | -0.082 |
| C6M | B9m | 0.151 |
| C7M | B9m | 0.094 |
| C8M | B9m | 0.003 |
| C9M | B9m | -0.005 |
| C10M | B9m | 0.005 |
| C11M | B9m | 0.025 |
| C12M | B9m | -0.069 |
| C13M | B9m | 0.021 |
| C14M | B9m | -0.057 |
| C15M | B9m | -0.065 |
| C16M | B9m | -0.044 |
| C17M | B9m | -0.015 |
| C18M | B9m | -0.048 |
| C19M | B9m | 0.040 |
| C20M | B9m | 0.022 |
| C21M | B9m | -0.102 |
| C22M | B9m | 0.126 |
| C23M | B9m | -0.036 |
| C24M | B9m | -0.053 |
| C25M | B9m | -0.063 |
| C26M | B9m | -0.087 |
| C27M | B9m | 0.003 |
| C28M | B9m | 0.016 |
| C29M | B9m | -0.008 |
| C30M | B9m | -0.039 |
| B1m | B9m | -0.037 |
| B2m | B9m | 0.033 |
| B3m | B9m | -0.026 |
| B4m | B9m | 0.076 |
| B5m | B9m | 0.025 |
| B6m | B9m | 0.075 |
| B7m | B9m | -0.078 |
| B8m | B9m | 0.024 |
| B1 | B10m | -0.010 |
| B2 | B10m | -0.075 |
| B3 | B10m | 0.025 |
| B4 | B10m | -0.030 |
| B5 | B10m | -0.052 |
| B6 | B10m | 0.069 |
| B7 | B10m | 0.059 |
| B8F | B10m | 0.028 |
| B9F | B10m | -0.017 |
| B10F | B10m | -0.012 |
| B11F | B10m | -0.009 |
| B12F | B10m | -0.074 |
| B13M | B10m | -0.053 |
| B14F | B10m | -0.021 |
| B15M | B10m | 0.123 |
| B16F | B10m | -0.092 |
| B17F | B10m | -0.065 |
| B18M | B10m | 0.176 |
| B19M | B10m | -0.009 |
| B20F | B10m | -0.049 |
| B21F | B10m | -0.078 |
| B22F | B10m | -0.024 |
| B23F | B10m | -0.096 |
| B24F | B10m | -0.064 |
| B25F | B10m | -0.032 |
| B26F | B10m | -0.023 |
| B27F | B10m | 0.006 |
| B28M | B10m | -0.009 |
| B29F | B10m | -0.054 |
| B30F | B10m | -0.074 |
| B31M | B10m | -0.096 |
| B32M | B10m | -0.024 |
| B33M | B10m | -0.038 |
| B34M | B10m | 0.057 |
| B35M | B10m | -0.049 |
| B36M | B10m | 0.014 |
| B37M | B10m | -0.034 |
| C1M | B10m | 0.147 |
| C2M | B10m | -0.033 |
| C3M | B10m | -0.039 |
| C4M | B10m | -0.053 |
| C5M | B10m | -0.032 |
| C6M | B10m | -0.067 |
| C7M | B10m | -0.029 |
| C8M | B10m | -0.003 |
| C9M | B10m | 0.030 |
| C10M | B10m | -0.047 |
| C11M | B10m | 0.061 |
| C12M | B10m | -0.035 |
| C13M | B10m | 0.005 |
| C14M | B10m | -0.072 |
| C15M | B10m | -0.068 |
| C16M | B10m | -0.037 |
| C17M | B10m | -0.062 |
| C18M | B10m | -0.110 |
| C19M | B10m | -0.056 |
| C20M | B10m | -0.013 |
| C21M | B10m | -0.055 |
| C22M | B10m | 0.091 |
| C23M | B10m | -0.078 |
| C24M | B10m | -0.085 |
| C25M | B10m | -0.030 |
| C26M | B10m | -0.030 |
| C27M | B10m | 0.017 |
| C28M | B10m | -0.046 |
| C29M | B10m | 0.179 |
| C30M | B10m | 0.053 |
| B1m | B10m | -0.024 |
| B2m | B10m | 0.062 |
| B3m | B10m | -0.010 |
| B4m | B10m | 0.103 |
| B5m | B10m | 0.027 |
| B6m | B10m | 0.042 |
| B7m | B10m | 0.038 |
| B8m | B10m | -0.030 |
| B9m | B10m | 0.060 |
| B1 | B11m | -0.017 |
| B2 | B11m | -0.075 |
| B3 | B11m | -0.005 |
| B4 | B11m | 0.030 |
| B5 | B11m | -0.015 |
| B6 | B11m | 0.294 |
| B7 | B11m | -0.014 |
| B8F | B11m | 0.081 |
| B9F | B11m | 0.051 |
| B10F | B11m | -0.019 |
| B11F | B11m | -0.022 |
| B12F | B11m | -0.036 |
| B13M | B11m | -0.061 |
| B14F | B11m | 0.072 |
| B15M | B11m | -0.089 |
| B16F | B11m | 0.011 |
| B17F | B11m | -0.122 |
| B18M | B11m | -0.093 |
| B19M | B11m | 0.076 |
| B20F | B11m | 0.111 |
| B21F | B11m | 0.101 |
| B22F | B11m | -0.018 |
| B23F | B11m | -0.070 |
| B24F | B11m | -0.110 |
| B25F | B11m | 0.071 |
| B26F | B11m | 0.024 |
| B27F | B11m | 0.105 |
| B28M | B11m | -0.072 |
| B29F | B11m | 0.107 |
| B30F | B11m | -0.028 |
| B31M | B11m | -0.073 |
| B32M | B11m | 0.036 |
| B33M | B11m | -0.072 |
| B34M | B11m | 0.008 |
| B35M | B11m | -0.005 |
| B36M | B11m | 0.067 |
| B37M | B11m | -0.096 |
| C1M | B11m | -0.056 |
| C2M | B11m | -0.003 |
| C3M | B11m | 0.013 |
| C4M | B11m | -0.018 |
| C5M | B11m | -0.008 |
| C6M | B11m | -0.106 |
| C7M | B11m | 0.012 |
| C8M | B11m | -0.074 |
| C9M | B11m | 0.013 |
| C10M | B11m | 0.038 |
| C11M | B11m | 0.041 |
| C12M | B11m | -0.050 |
| C13M | B11m | 0.002 |
| C14M | B11m | -0.010 |
| C15M | B11m | -0.030 |
| C16M | B11m | -0.033 |
| C17M | B11m | -0.065 |
| C18M | B11m | -0.033 |
| C19M | B11m | -0.020 |
| C20M | B11m | 0.004 |
| C21M | B11m | -0.067 |
| C22M | B11m | -0.044 |
| C23M | B11m | 0.053 |
| C24M | B11m | -0.029 |
| C25M | B11m | 0.075 |
| C26M | B11m | -0.049 |
| C27M | B11m | -0.034 |
| C28M | B11m | -0.021 |
| C29M | B11m | 0.030 |
| C30M | B11m | -0.006 |
| B1m | B11m | -0.012 |
| B2m | B11m | -0.026 |
| B3m | B11m | -0.049 |
| B4m | B11m | -0.006 |
| B5m | B11m | -0.054 |
| B6m | B11m | -0.056 |
| B7m | B11m | 0.091 |
| B8m | B11m | 0.074 |
| B9m | B11m | -0.063 |
| B10m | B11m | 0.044 |
| B1 | B12m | -0.003 |
| B2 | B12m | -0.030 |
| B3 | B12m | 0.000 |
| B4 | B12m | 0.056 |
| B5 | B12m | 0.010 |
| B6 | B12m | -0.015 |
| B7 | B12m | -0.029 |
| B8F | B12m | 0.122 |
| B9F | B12m | 0.070 |
| B10F | B12m | 0.029 |
| B11F | B12m | -0.053 |
| B12F | B12m | -0.074 |
| B13M | B12m | -0.070 |
| B14F | B12m | -0.026 |
| B15M | B12m | 0.000 |
| B16F | B12m | 0.006 |
| B17F | B12m | -0.122 |
| B18M | B12m | -0.098 |
| B19M | B12m | -0.105 |
| B20F | B12m | -0.035 |
| B21F | B12m | 0.063 |
| B22F | B12m | 0.000 |
| B23F | B12m | -0.009 |
| B24F | B12m | -0.072 |
| B25F | B12m | -0.006 |
| B26F | B12m | -0.064 |
| B27F | B12m | 0.068 |
| B28M | B12m | -0.026 |
| B29F | B12m | -0.074 |
| B30F | B12m | -0.017 |
| B31M | B12m | -0.044 |
| B32M | B12m | -0.014 |
| B33M | B12m | -0.005 |
| B34M | B12m | -0.038 |
| B35M | B12m | 0.015 |
| B36M | B12m | -0.033 |
| B37M | B12m | -0.132 |
| C1M | B12m | -0.043 |
| C2M | B12m | 0.135 |
| C3M | B12m | -0.011 |
| C4M | B12m | -0.032 |
| C5M | B12m | 0.009 |
| C6M | B12m | -0.036 |
| C7M | B12m | 0.080 |
| C8M | B12m | -0.063 |
| C9M | B12m | -0.057 |
| C10M | B12m | -0.037 |
| C11M | B12m | 0.049 |
| C12M | B12m | 0.044 |
| C13M | B12m | -0.027 |
| C14M | B12m | -0.033 |
| C15M | B12m | -0.068 |
| C16M | B12m | -0.012 |
| C17M | B12m | -0.104 |
| C18M | B12m | -0.028 |
| C19M | B12m | -0.021 |
| C20M | B12m | 0.033 |
| C21M | B12m | -0.045 |
| C22M | B12m | -0.012 |
| C23M | B12m | -0.022 |
| C24M | B12m | 0.011 |
| C25M | B12m | -0.050 |
| C26M | B12m | -0.004 |
| C27M | B12m | 0.000 |
| C28M | B12m | -0.030 |
| C29M | B12m | -0.019 |
| C30M | B12m | -0.079 |
| B1m | B12m | -0.066 |
| B2m | B12m | 0.034 |
| B3m | B12m | -0.071 |
| B4m | B12m | 0.109 |
| B5m | B12m | 0.010 |
| B6m | B12m | 0.113 |
| B7m | B12m | 0.048 |
| B8m | B12m | -0.050 |
| B9m | B12m | 0.123 |
| B10m | B12m | 0.017 |
| B11m | B12m | 0.036 |
| B1 | B13m | -0.073 |
| B2 | B13m | -0.015 |
| B3 | B13m | -0.013 |
| B4 | B13m | 0.002 |
| B5 | B13m | 0.057 |
| B6 | B13m | -0.006 |
| B7 | B13m | -0.085 |
| B8F | B13m | -0.063 |
| B9F | B13m | -0.058 |
| B10F | B13m | -0.032 |
| B11F | B13m | -0.014 |
| B12F | B13m | 0.009 |
| B13M | B13m | 0.011 |
| B14F | B13m | -0.037 |
| B15M | B13m | -0.029 |
| B16F | B13m | 0.042 |
| B17F | B13m | -0.019 |
| B18M | B13m | -0.070 |
| B19M | B13m | -0.016 |
| B20F | B13m | -0.032 |
| B21F | B13m | 0.025 |
| B22F | B13m | -0.049 |
| B23F | B13m | -0.066 |
| B24F | B13m | -0.010 |
| B25F | B13m | -0.010 |
| B26F | B13m | -0.100 |
| B27F | B13m | -0.034 |
| B28M | B13m | -0.060 |
| B29F | B13m | -0.042 |
| B30F | B13m | -0.034 |
| B31M | B13m | 0.063 |
| B32M | B13m | 0.002 |
| B33M | B13m | 0.042 |
| B34M | B13m | -0.049 |
| B35M | B13m | 0.022 |
| B36M | B13m | -0.067 |
| B37M | B13m | -0.024 |
| C1M | B13m | -0.039 |
| C2M | B13m | 0.009 |
| C3M | B13m | 0.028 |
| C4M | B13m | -0.038 |
| C5M | B13m | 0.058 |
| C6M | B13m | -0.014 |
| C7M | B13m | -0.023 |
| C8M | B13m | -0.100 |
| C9M | B13m | -0.056 |
| C10M | B13m | 0.010 |
| C11M | B13m | 0.050 |
| C12M | B13m | -0.039 |
| C13M | B13m | -0.115 |
| C14M | B13m | 0.002 |
| C15M | B13m | -0.021 |
| C16M | B13m | -0.062 |
| C17M | B13m | 0.020 |
| C18M | B13m | -0.100 |
| C19M | B13m | 0.058 |
| C20M | B13m | -0.001 |
| C21M | B13m | -0.109 |
| C22M | B13m | -0.062 |
| C23M | B13m | -0.083 |
| C24M | B13m | -0.003 |
| C25M | B13m | -0.023 |
| C26M | B13m | -0.042 |
| C27M | B13m | -0.063 |
| C28M | B13m | -0.055 |
| C29M | B13m | -0.016 |
| C30M | B13m | -0.072 |
| B1m | B13m | 0.011 |
| B2m | B13m | 0.054 |
| B3m | B13m | -0.058 |
| B4m | B13m | 0.042 |
| B5m | B13m | 0.180 |
| B6m | B13m | 0.131 |
| B7m | B13m | -0.011 |
| B8m | B13m | -0.043 |
| B9m | B13m | -0.025 |
| B10m | B13m | -0.030 |
| B11m | B13m | 0.002 |
| B12m | B13m | 0.112 |
| B1 | B14m | -0.020 |
| B2 | B14m | -0.089 |
| B3 | B14m | -0.024 |
| B4 | B14m | 0.011 |
| B5 | B14m | -0.009 |
| B6 | B14m | -0.090 |
| B7 | B14m | 0.027 |
| B8F | B14m | -0.008 |
| B9F | B14m | -0.011 |
| B10F | B14m | 0.052 |
| B11F | B14m | -0.012 |
| B12F | B14m | -0.038 |
| B13M | B14m | -0.088 |
| B14F | B14m | -0.034 |
| B15M | B14m | -0.033 |
| B16F | B14m | -0.035 |
| B17F | B14m | -0.055 |
| B18M | B14m | -0.049 |
| B19M | B14m | -0.118 |
| B20F | B14m | -0.084 |
| B21F | B14m | -0.063 |
| B22F | B14m | -0.046 |
| B23F | B14m | -0.018 |
| B24F | B14m | -0.031 |
| B25F | B14m | 0.070 |
| B26F | B14m | -0.079 |
| B27F | B14m | -0.034 |
| B28M | B14m | -0.045 |
| B29F | B14m | -0.051 |
| B30F | B14m | -0.034 |
| B31M | B14m | -0.062 |
| B32M | B14m | 0.025 |
| B33M | B14m | 0.020 |
| B34M | B14m | -0.045 |
| B35M | B14m | 0.013 |
| B36M | B14m | -0.080 |
| B37M | B14m | -0.023 |
| C1M | B14m | -0.071 |
| C2M | B14m | -0.014 |
| C3M | B14m | -0.048 |
| C4M | B14m | -0.030 |
| C5M | B14m | -0.043 |
| C6M | B14m | 0.058 |
| C7M | B14m | 0.005 |
| C8M | B14m | -0.092 |
| C9M | B14m | -0.033 |
| C10M | B14m | -0.043 |
| C11M | B14m | 0.019 |
| C12M | B14m | 0.024 |
| C13M | B14m | 0.092 |
| C14M | B14m | 0.022 |
| C15M | B14m | -0.036 |
| C16M | B14m | -0.040 |
| C17M | B14m | -0.036 |
| C18M | B14m | 0.042 |
| C19M | B14m | -0.026 |
| C20M | B14m | -0.062 |
| C21M | B14m | -0.116 |
| C22M | B14m | 0.023 |
| C23M | B14m | -0.008 |
| C24M | B14m | 0.002 |
| C25M | B14m | -0.051 |
| C26M | B14m | -0.021 |
| C27M | B14m | -0.069 |
| C28M | B14m | -0.080 |
| C29M | B14m | -0.011 |
| C30M | B14m | -0.087 |
| B1m | B14m | 0.028 |
| B2m | B14m | 0.037 |
| B3m | B14m | -0.038 |
| B4m | B14m | -0.025 |
| B5m | B14m | 0.151 |
| B6m | B14m | 0.083 |
| B7m | B14m | -0.122 |
| B8m | B14m | -0.070 |
| B9m | B14m | 0.088 |
| B10m | B14m | -0.046 |
| B11m | B14m | -0.078 |
| B12m | B14m | 0.105 |
| B13m | B14m | 0.265 |
| B1 | B15m | -0.075 |
| B2 | B15m | -0.021 |
| B3 | B15m | -0.031 |
| B4 | B15m | 0.066 |
| B5 | B15m | -0.014 |
| B6 | B15m | -0.030 |
| B7 | B15m | -0.019 |
| B8F | B15m | -0.058 |
| B9F | B15m | -0.027 |
| B10F | B15m | -0.002 |
| B11F | B15m | -0.018 |
| B12F | B15m | -0.078 |
| B13M | B15m | -0.047 |
| B14F | B15m | -0.023 |
| B15M | B15m | -0.061 |
| B16F | B15m | 0.006 |
| B17F | B15m | -0.079 |
| B18M | B15m | -0.035 |
| B19M | B15m | -0.094 |
| B20F | B15m | 0.023 |
| B21F | B15m | 0.039 |
| B22F | B15m | -0.026 |
| B23F | B15m | -0.100 |
| B24F | B15m | -0.059 |
| B25F | B15m | 0.104 |
| B26F | B15m | -0.038 |
| B27F | B15m | -0.002 |
| B28M | B15m | -0.038 |
| B29F | B15m | -0.041 |
| B30F | B15m | 0.059 |
| B31M | B15m | -0.089 |
| B32M | B15m | -0.082 |
| B33M | B15m | -0.046 |
| B34M | B15m | -0.083 |
| B35M | B15m | -0.096 |
| B36M | B15m | -0.046 |
| B37M | B15m | -0.028 |
| C1M | B15m | -0.033 |
| C2M | B15m | 0.024 |
| C3M | B15m | -0.103 |
| C4M | B15m | -0.083 |
| C5M | B15m | 0.022 |
| C6M | B15m | 0.097 |
| C7M | B15m | -0.020 |
| C8M | B15m | -0.004 |
| C9M | B15m | -0.044 |
| C10M | B15m | 0.012 |
| C11M | B15m | 0.037 |
| C12M | B15m | -0.038 |
| C13M | B15m | -0.028 |
| C14M | B15m | -0.097 |
| C15M | B15m | 0.013 |
| C16M | B15m | -0.038 |
| C17M | B15m | -0.090 |
| C18M | B15m | 0.031 |
| C19M | B15m | -0.072 |
| C20M | B15m | 0.001 |
| C21M | B15m | -0.083 |
| C22M | B15m | 0.087 |
| C23M | B15m | -0.021 |
| C24M | B15m | 0.077 |
| C25M | B15m | -0.074 |
| C26M | B15m | 0.041 |
| C27M | B15m | -0.030 |
| C28M | B15m | -0.051 |
| C29M | B15m | 0.005 |
| C30M | B15m | -0.075 |
| B1m | B15m | 0.018 |
| B2m | B15m | 0.027 |
| B3m | B15m | -0.072 |
| B4m | B15m | -0.031 |
| B5m | B15m | 0.000 |
| B6m | B15m | 0.018 |
| B7m | B15m | 0.078 |
| B8m | B15m | 0.021 |
| B9m | B15m | 0.041 |
| B10m | B15m | -0.047 |
| B11m | B15m | -0.033 |
| B12m | B15m | 0.119 |
| B13m | B15m | 0.139 |
| B14m | B15m | 0.236 |
| B1 | B16m | -0.066 |
| B2 | B16m | -0.013 |
| B3 | B16m | 0.188 |
| B4 | B16m | -0.042 |
| B5 | B16m | 0.052 |
| B6 | B16m | -0.061 |
| B7 | B16m | -0.093 |
| B8F | B16m | 0.294 |
| B9F | B16m | -0.046 |
| B10F | B16m | -0.014 |
| B11F | B16m | -0.054 |
| B12F | B16m | 0.029 |
| B13M | B16m | -0.008 |
| B14F | B16m | -0.058 |
| B15M | B16m | 0.027 |
| B16F | B16m | -0.041 |
| B17F | B16m | -0.051 |
| B18M | B16m | -0.061 |
| B19M | B16m | -0.051 |
| B20F | B16m | 0.013 |
| B21F | B16m | -0.047 |
| B22F | B16m | -0.051 |
| B23F | B16m | -0.073 |
| B24F | B16m | 0.026 |
| B25F | B16m | -0.038 |
| B26F | B16m | -0.008 |
| B27F | B16m | -0.035 |
| B28M | B16m | -0.077 |
| B29F | B16m | 0.015 |
| B30F | B16m | 0.015 |
| B31M | B16m | 0.177 |
| B32M | B16m | 0.017 |
| B33M | B16m | 0.029 |
| B34M | B16m | 0.082 |
| B35M | B16m | 0.006 |
| B36M | B16m | -0.073 |
| B37M | B16m | -0.022 |
| C1M | B16m | -0.082 |
| C2M | B16m | -0.060 |
| C3M | B16m | -0.056 |
| C4M | B16m | -0.042 |
| C5M | B16m | 0.012 |
| C6M | B16m | -0.027 |
| C7M | B16m | 0.034 |
| C8M | B16m | -0.023 |
| C9M | B16m | -0.062 |
| C10M | B16m | -0.076 |
| C11M | B16m | 0.028 |
| C12M | B16m | -0.093 |
| C13M | B16m | -0.100 |
| C14M | B16m | -0.060 |
| C15M | B16m | -0.003 |
| C16M | B16m | 0.042 |
| C17M | B16m | -0.102 |
| C18M | B16m | -0.008 |
| C19M | B16m | -0.036 |
| C20M | B16m | -0.071 |
| C21M | B16m | -0.028 |
| C22M | B16m | -0.063 |
| C23M | B16m | -0.037 |
| C24M | B16m | 0.021 |
| C25M | B16m | -0.044 |
| C26M | B16m | 0.046 |
| C27M | B16m | -0.025 |
| C28M | B16m | -0.055 |
| C29M | B16m | 0.048 |
| C30M | B16m | -0.029 |
| B1m | B16m | -0.033 |
| B2m | B16m | 0.016 |
| B3m | B16m | 0.033 |
| B4m | B16m | 0.137 |
| B5m | B16m | 0.268 |
| B6m | B16m | 0.091 |
| B7m | B16m | -0.064 |
| B8m | B16m | -0.102 |
| B9m | B16m | -0.042 |
| B10m | B16m | 0.050 |
| B11m | B16m | 0.000 |
| B12m | B16m | -0.002 |
| B13m | B16m | 0.016 |
| B14m | B16m | -0.032 |
| B15m | B16m | -0.067 |
| B1 | B17m | -0.029 |
| B2 | B17m | -0.002 |
| B3 | B17m | -0.062 |
| B4 | B17m | -0.009 |
| B5 | B17m | 0.054 |
| B6 | B17m | -0.028 |
| B7 | B17m | -0.027 |
| B8F | B17m | -0.045 |
| B9F | B17m | -0.058 |
| B10F | B17m | -0.006 |
| B11F | B17m | -0.021 |
| B12F | B17m | -0.011 |
| B13M | B17m | -0.021 |
| B14F | B17m | -0.054 |
| B15M | B17m | -0.075 |
| B16F | B17m | 0.016 |
| B17F | B17m | -0.030 |
| B18M | B17m | -0.093 |
| B19M | B17m | -0.010 |
| B20F | B17m | -0.071 |
| B21F | B17m | 0.110 |
| B22F | B17m | -0.068 |
| B23F | B17m | -0.036 |
| B24F | B17m | 0.045 |
| B25F | B17m | 0.081 |
| B26F | B17m | -0.013 |
| B27F | B17m | -0.017 |
| B28M | B17m | -0.033 |
| B29F | B17m | -0.127 |
| B30F | B17m | 0.019 |
| B31M | B17m | -0.050 |
| B32M | B17m | -0.050 |
| B33M | B17m | -0.030 |
| B34M | B17m | -0.095 |
| B35M | B17m | -0.091 |
| B36M | B17m | -0.020 |
| B37M | B17m | -0.034 |
| C1M | B17m | 0.014 |
| C2M | B17m | -0.004 |
| C3M | B17m | 0.005 |
| C4M | B17m | -0.017 |
| C5M | B17m | 0.012 |
| C6M | B17m | -0.054 |
| C7M | B17m | -0.069 |
| C8M | B17m | -0.049 |
| C9M | B17m | -0.043 |
| C10M | B17m | -0.001 |
| C11M | B17m | -0.017 |
| C12M | B17m | -0.084 |
| C13M | B17m | -0.091 |
| C14M | B17m | -0.005 |
| C15M | B17m | 0.162 |
| C16M | B17m | 0.024 |
| C17M | B17m | 0.047 |
| C18M | B17m | 0.083 |
| C19M | B17m | 0.009 |
| C20M | B17m | 0.017 |
| C21M | B17m | -0.010 |
| C22M | B17m | 0.016 |
| C23M | B17m | -0.037 |
| C24M | B17m | -0.021 |
| C25M | B17m | 0.005 |
| C26M | B17m | -0.005 |
| C27M | B17m | -0.018 |
| C28M | B17m | -0.054 |
| C29M | B17m | 0.051 |
| C30M | B17m | -0.045 |
| B1m | B17m | 0.027 |
| B2m | B17m | 0.028 |
| B3m | B17m | 0.310 |
| B4m | B17m | -0.052 |
| B5m | B17m | -0.009 |
| B6m | B17m | -0.044 |
| B7m | B17m | 0.230 |
| B8m | B17m | 0.074 |
| B9m | B17m | -0.044 |
| B10m | B17m | -0.048 |
| B11m | B17m | -0.035 |
| B12m | B17m | 0.016 |
| B13m | B17m | 0.016 |
| B14m | B17m | -0.080 |
| B15m | B17m | 0.174 |
| B16m | B17m | -0.039 |
| B1 | B18m | -0.058 |
| B2 | B18m | -0.064 |
| B3 | B18m | 0.035 |
| B4 | B18m | 0.073 |
| B5 | B18m | -0.031 |
| B6 | B18m | -0.036 |
| B7 | B18m | -0.019 |
| B8F | B18m | -0.108 |
| B9F | B18m | -0.056 |
| B10F | B18m | 0.082 |
| B11F | B18m | -0.055 |
| B12F | B18m | -0.044 |
| B13M | B18m | -0.060 |
| B14F | B18m | -0.026 |
| B15M | B18m | 0.042 |
| B16F | B18m | -0.035 |
| B17F | B18m | -0.074 |
| B18M | B18m | -0.070 |
| B19M | B18m | -0.043 |
| B20F | B18m | -0.080 |
| B21F | B18m | -0.019 |
| B22F | B18m | -0.017 |
| B23F | B18m | -0.071 |
| B24F | B18m | -0.099 |
| B25F | B18m | 0.025 |
| B26F | B18m | -0.035 |
| B27F | B18m | -0.020 |
| B28M | B18m | -0.066 |
| B29F | B18m | -0.122 |
| B30F | B18m | -0.007 |
| B31M | B18m | -0.041 |
| B32M | B18m | 0.062 |
| B33M | B18m | -0.024 |
| B34M | B18m | 0.037 |
| B35M | B18m | -0.064 |
| B36M | B18m | -0.001 |
| B37M | B18m | -0.026 |
| C1M | B18m | -0.047 |
| C2M | B18m | -0.020 |
| C3M | B18m | -0.061 |
| C4M | B18m | -0.012 |
| C5M | B18m | 0.001 |
| C6M | B18m | 0.128 |
| C7M | B18m | -0.068 |
| C8M | B18m | -0.058 |
| C9M | B18m | -0.021 |
| C10M | B18m | -0.076 |
| C11M | B18m | 0.067 |
| C12M | B18m | -0.028 |
| C13M | B18m | 0.021 |
| C14M | B18m | -0.031 |
| C15M | B18m | -0.016 |
| C16M | B18m | -0.026 |
| C17M | B18m | -0.027 |
| C18M | B18m | -0.107 |
| C19M | B18m | -0.051 |
| C20M | B18m | -0.019 |
| C21M | B18m | -0.085 |
| C22M | B18m | -0.098 |
| C23M | B18m | -0.085 |
| C24M | B18m | -0.029 |
| C25M | B18m | -0.021 |
| C26M | B18m | -0.047 |
| C27M | B18m | -0.041 |
| C28M | B18m | -0.051 |
| C29M | B18m | 0.019 |
| C30M | B18m | -0.048 |
| B1m | B18m | 0.013 |
| B2m | B18m | 0.105 |
| B3m | B18m | 0.044 |
| B4m | B18m | 0.053 |
| B5m | B18m | 0.116 |
| B6m | B18m | 0.055 |
| B7m | B18m | -0.058 |
| B8m | B18m | -0.024 |
| B9m | B18m | -0.008 |
| B10m | B18m | 0.126 |
| B11m | B18m | 0.004 |
| B12m | B18m | -0.023 |
| B13m | B18m | 0.193 |
| B14m | B18m | 0.144 |
| B15m | B18m | 0.047 |
| B16m | B18m | 0.128 |
| B17m | B18m | 0.014 |
| B1 | B19m | 0.029 |
| B2 | B19m | -0.071 |
| B3 | B19m | 0.114 |
| B4 | B19m | 0.006 |
| B5 | B19m | 0.023 |
| B6 | B19m | -0.058 |
| B7 | B19m | -0.014 |
| B8F | B19m | -0.052 |
| B9F | B19m | -0.069 |
| B10F | B19m | -0.014 |
| B11F | B19m | -0.039 |
| B12F | B19m | 0.032 |
| B13M | B19m | -0.060 |
| B14F | B19m | 0.020 |
| B15M | B19m | 0.097 |
| B16F | B19m | -0.002 |
| B17F | B19m | -0.005 |
| B18M | B19m | -0.001 |
| B19M | B19m | 0.020 |
| B20F | B19m | -0.037 |
| B21F | B19m | 0.026 |
| B22F | B19m | 0.165 |
| B23F | B19m | 0.039 |
| B24F | B19m | -0.003 |
| B25F | B19m | -0.087 |
| B26F | B19m | -0.081 |
| B27F | B19m | -0.034 |
| B28M | B19m | 0.048 |
| B29F | B19m | -0.078 |
| B30F | B19m | 0.041 |
| B31M | B19m | -0.024 |
| B32M | B19m | 0.021 |
| B33M | B19m | 0.010 |
| B34M | B19m | 0.077 |
| B35M | B19m | -0.015 |
| B36M | B19m | 0.204 |
| B37M | B19m | -0.072 |
| C1M | B19m | 0.130 |
| C2M | B19m | 0.068 |
| C3M | B19m | 0.050 |
| C4M | B19m | -0.030 |
| C5M | B19m | 0.014 |
| C6M | B19m | -0.074 |
| C7M | B19m | -0.046 |
| C8M | B19m | 0.008 |
| C9M | B19m | 0.001 |
| C10M | B19m | -0.013 |
| C11M | B19m | 0.041 |
| C12M | B19m | -0.003 |
| C13M | B19m | -0.032 |
| C14M | B19m | -0.065 |
| C15M | B19m | -0.029 |
| C16M | B19m | 0.043 |
| C17M | B19m | -0.079 |
| C18M | B19m | -0.081 |
| C19M | B19m | -0.005 |
| C20M | B19m | 0.045 |
| C21M | B19m | -0.060 |
| C22M | B19m | -0.060 |
| C23M | B19m | -0.046 |
| C24M | B19m | -0.012 |
| C25M | B19m | 0.046 |
| C26M | B19m | 0.003 |
| C27M | B19m | -0.023 |
| C28M | B19m | -0.093 |
| C29M | B19m | -0.019 |
| C30M | B19m | 0.012 |
| B1m | B19m | -0.035 |
| B2m | B19m | 0.037 |
| B3m | B19m | -0.079 |
| B4m | B19m | 0.096 |
| B5m | B19m | -0.047 |
| B6m | B19m | -0.019 |
| B7m | B19m | -0.063 |
| B8m | B19m | -0.025 |
| B9m | B19m | 0.007 |
| B10m | B19m | 0.010 |
| B11m | B19m | -0.042 |
| B12m | B19m | 0.048 |
| B13m | B19m | 0.030 |
| B14m | B19m | 0.011 |
| B15m | B19m | 0.001 |
| B16m | B19m | -0.002 |
| B17m | B19m | 0.000 |
| B18m | B19m | 0.027 |
| B1 | B20m | -0.083 |
| B2 | B20m | 0.063 |
| B3 | B20m | -0.016 |
| B4 | B20m | 0.044 |
| B5 | B20m | 0.016 |
| B6 | B20m | -0.086 |
| B7 | B20m | -0.044 |
| B8F | B20m | -0.037 |
| B9F | B20m | -0.061 |
| B10F | B20m | -0.048 |
| B11F | B20m | 0.011 |
| B12F | B20m | 0.005 |
| B13M | B20m | -0.111 |
| B14F | B20m | 0.070 |
| B15M | B20m | 0.047 |
| B16F | B20m | 0.048 |
| B17F | B20m | -0.050 |
| B18M | B20m | -0.037 |
| B19M | B20m | -0.122 |
| B20F | B20m | -0.017 |
| B21F | B20m | 0.103 |
| B22F | B20m | -0.051 |
| B23F | B20m | 0.000 |
| B24F | B20m | -0.059 |
| B25F | B20m | 0.065 |
| B26F | B20m | -0.058 |
| B27F | B20m | 0.104 |
| B28M | B20m | -0.061 |
| B29F | B20m | -0.060 |
| B30F | B20m | 0.035 |
| B31M | B20m | -0.029 |
| B32M | B20m | -0.014 |
| B33M | B20m | -0.028 |
| B34M | B20m | 0.040 |
| B35M | B20m | -0.026 |
| B36M | B20m | -0.078 |
| B37M | B20m | -0.012 |
| C1M | B20m | -0.068 |
| C2M | B20m | 0.049 |
| C3M | B20m | 0.045 |
| C4M | B20m | -0.036 |
| C5M | B20m | -0.025 |
| C6M | B20m | -0.033 |
| C7M | B20m | 0.077 |
| C8M | B20m | -0.065 |
| C9M | B20m | -0.037 |
| C10M | B20m | -0.008 |
| C11M | B20m | 0.026 |
| C12M | B20m | -0.036 |
| C13M | B20m | -0.086 |
| C14M | B20m | -0.065 |
| C15M | B20m | -0.037 |
| C16M | B20m | -0.042 |
| C17M | B20m | 0.016 |
| C18M | B20m | -0.030 |
| C19M | B20m | -0.038 |
| C20M | B20m | 0.003 |
| C21M | B20m | -0.136 |
| C22M | B20m | -0.079 |
| C23M | B20m | -0.039 |
| C24M | B20m | -0.010 |
| C25M | B20m | -0.077 |
| C26M | B20m | -0.062 |
| C27M | B20m | -0.033 |
| C28M | B20m | 0.055 |
| C29M | B20m | -0.043 |
| C30M | B20m | -0.093 |
| B1m | B20m | 0.009 |
| B2m | B20m | -0.011 |
| B3m | B20m | 0.001 |
| B4m | B20m | 0.002 |
| B5m | B20m | 0.056 |
| B6m | B20m | 0.062 |
| B7m | B20m | -0.111 |
| B8m | B20m | -0.065 |
| B9m | B20m | 0.091 |
| B10m | B20m | -0.072 |
| B11m | B20m | 0.040 |
| B12m | B20m | 0.070 |
| B13m | B20m | 0.211 |
| B14m | B20m | 0.208 |
| B15m | B20m | 0.097 |
| B16m | B20m | -0.012 |
| B17m | B20m | -0.049 |
| B18m | B20m | 0.085 |
| B19m | B20m | -0.023 |
| B1 | B21m | 0.020 |
| B2 | B21m | -0.002 |
| B3 | B21m | -0.074 |
| B4 | B21m | -0.048 |
| B5 | B21m | -0.035 |
| B6 | B21m | -0.044 |
| B7 | B21m | -0.053 |
| B8F | B21m | 0.090 |
| B9F | B21m | 0.021 |
| B10F | B21m | -0.094 |
| B11F | B21m | -0.003 |
| B12F | B21m | -0.007 |
| B13M | B21m | -0.093 |
| B14F | B21m | -0.015 |
| B15M | B21m | 0.049 |
| B16F | B21m | -0.006 |
| B17F | B21m | -0.121 |
| B18M | B21m | -0.032 |
| B19M | B21m | -0.105 |
| B20F | B21m | 0.086 |
| B21F | B21m | -0.029 |
| B22F | B21m | -0.086 |
| B23F | B21m | -0.078 |
| B24F | B21m | -0.109 |
| B25F | B21m | 0.176 |
| B26F | B21m | -0.032 |
| B27F | B21m | 0.014 |
| B28M | B21m | -0.041 |
| B29F | B21m | 0.018 |
| B30F | B21m | -0.030 |
| B31M | B21m | -0.032 |
| B32M | B21m | 0.013 |
| B33M | B21m | -0.085 |
| B34M | B21m | 0.075 |
| B35M | B21m | -0.035 |
| B36M | B21m | 0.000 |
| B37M | B21m | 0.003 |
| C1M | B21m | -0.054 |
| C2M | B21m | 0.021 |
| C3M | B21m | 0.021 |
| C4M | B21m | -0.005 |
| C5M | B21m | 0.006 |
| C6M | B21m | -0.063 |
| C7M | B21m | 0.043 |
| C8M | B21m | -0.040 |
| C9M | B21m | -0.019 |
| C10M | B21m | 0.085 |
| C11M | B21m | -0.034 |
| C12M | B21m | -0.043 |
| C13M | B21m | 0.007 |
| C14M | B21m | -0.052 |
| C15M | B21m | -0.044 |
| C16M | B21m | -0.018 |
| C17M | B21m | -0.026 |
| C18M | B21m | -0.039 |
| C19M | B21m | -0.033 |
| C20M | B21m | -0.084 |
| C21M | B21m | -0.095 |
| C22M | B21m | -0.065 |
| C23M | B21m | 0.059 |
| C24M | B21m | -0.007 |
| C25M | B21m | -0.074 |
| C26M | B21m | -0.036 |
| C27M | B21m | -0.001 |
| C28M | B21m | -0.066 |
| C29M | B21m | 0.001 |
| C30M | B21m | -0.055 |
| B1m | B21m | 0.060 |
| B2m | B21m | 0.042 |
| B3m | B21m | -0.072 |
| B4m | B21m | 0.020 |
| B5m | B21m | 0.034 |
| B6m | B21m | 0.071 |
| B7m | B21m | -0.116 |
| B8m | B21m | -0.138 |
| B9m | B21m | 0.138 |
| B10m | B21m | -0.005 |
| B11m | B21m | 0.058 |
| B12m | B21m | 0.034 |
| B13m | B21m | 0.051 |
| B14m | B21m | 0.156 |
| B15m | B21m | 0.086 |
| B16m | B21m | 0.006 |
| B17m | B21m | -0.033 |
| B18m | B21m | 0.079 |
| B19m | B21m | -0.028 |
| B20m | B21m | 0.230 |
| B1 | B22m | -0.074 |
| B2 | B22m | 0.009 |
| B3 | B22m | -0.028 |
| B4 | B22m | -0.051 |
| B5 | B22m | 0.049 |
| B6 | B22m | 0.038 |
| B7 | B22m | 0.014 |
| B8F | B22m | -0.038 |
| B9F | B22m | 0.035 |
| B10F | B22m | -0.047 |
| B11F | B22m | 0.070 |
| B12F | B22m | 0.014 |
| B13M | B22m | -0.064 |
| B14F | B22m | 0.052 |
| B15M | B22m | -0.006 |
| B16F | B22m | -0.097 |
| B17F | B22m | -0.069 |
| B18M | B22m | 0.051 |
| B19M | B22m | 0.018 |
| B20F | B22m | 0.063 |
| B21F | B22m | -0.071 |
| B22F | B22m | 0.028 |
| B23F | B22m | -0.053 |
| B24F | B22m | 0.006 |
| B25F | B22m | -0.086 |
| B26F | B22m | 0.059 |
| B27F | B22m | -0.038 |
| B28M | B22m | -0.049 |
| B29F | B22m | -0.066 |
| B30F | B22m | -0.056 |
| B31M | B22m | -0.035 |
| B32M | B22m | -0.025 |
| B33M | B22m | 0.029 |
| B34M | B22m | -0.002 |
| B35M | B22m | -0.074 |
| B36M | B22m | -0.007 |
| B37M | B22m | -0.006 |
| C1M | B22m | -0.041 |
| C2M | B22m | 0.030 |
| C3M | B22m | -0.048 |
| C4M | B22m | -0.063 |
| C5M | B22m | -0.098 |
| C6M | B22m | 0.165 |
| C7M | B22m | 0.070 |
| C8M | B22m | 0.069 |
| C9M | B22m | 0.003 |
| C10M | B22m | -0.036 |
| C11M | B22m | -0.003 |
| C12M | B22m | -0.031 |
| C13M | B22m | 0.073 |
| C14M | B22m | -0.054 |
| C15M | B22m | -0.061 |
| C16M | B22m | -0.021 |
| C17M | B22m | 0.060 |
| C18M | B22m | 0.010 |
| C19M | B22m | -0.063 |
| C20M | B22m | 0.008 |
| C21M | B22m | -0.057 |
| C22M | B22m | -0.042 |
| C23M | B22m | -0.022 |
| C24M | B22m | 0.052 |
| C25M | B22m | 0.044 |
| C26M | B22m | 0.014 |
| C27M | B22m | 0.077 |
| C28M | B22m | -0.039 |
| C29M | B22m | -0.023 |
| C30M | B22m | 0.064 |
| B1m | B22m | -0.007 |
| B2m | B22m | 0.010 |
| B3m | B22m | -0.060 |
| B4m | B22m | -0.061 |
| B5m | B22m | -0.006 |
| B6m | B22m | -0.066 |
| B7m | B22m | 0.020 |
| B8m | B22m | 0.225 |
| B9m | B22m | 0.177 |
| B10m | B22m | 0.008 |
| B11m | B22m | -0.020 |
| B12m | B22m | -0.039 |
| B13m | B22m | -0.049 |
| B14m | B22m | -0.014 |
| B15m | B22m | -0.029 |
| B16m | B22m | -0.059 |
| B17m | B22m | -0.089 |
| B18m | B22m | -0.055 |
| B19m | B22m | -0.090 |
| B20m | B22m | 0.046 |
| B21m | B22m | -0.028 |
| B1 | B23m | 0.002 |
| B2 | B23m | -0.120 |
| B3 | B23m | -0.061 |
| B4 | B23m | 0.059 |
| B5 | B23m | -0.023 |
| B6 | B23m | -0.016 |
| B7 | B23m | -0.060 |
| B8F | B23m | -0.081 |
| B9F | B23m | -0.023 |
| B10F | B23m | -0.050 |
| B11F | B23m | -0.022 |
| B12F | B23m | 0.022 |
| B13M | B23m | -0.041 |
| B14F | B23m | -0.067 |
| B15M | B23m | -0.030 |
| B16F | B23m | 0.046 |
| B17F | B23m | 0.046 |
| B18M | B23m | -0.099 |
| B19M | B23m | -0.034 |
| B20F | B23m | 0.017 |
| B21F | B23m | 0.010 |
| B22F | B23m | -0.073 |
| B23F | B23m | -0.122 |
| B24F | B23m | -0.034 |
| B25F | B23m | 0.178 |
| B26F | B23m | 0.054 |
| B27F | B23m | 0.058 |
| B28M | B23m | 0.171 |
| B29F | B23m | -0.109 |
| B30F | B23m | -0.025 |
| B31M | B23m | -0.057 |
| B32M | B23m | -0.034 |
| B33M | B23m | 0.027 |
| B34M | B23m | -0.046 |
| B35M | B23m | -0.026 |
| B36M | B23m | -0.036 |
| B37M | B23m | -0.022 |
| C1M | B23m | -0.125 |
| C2M | B23m | -0.050 |
| C3M | B23m | -0.042 |
| C4M | B23m | -0.070 |
| C5M | B23m | -0.025 |
| C6M | B23m | 0.152 |
| C7M | B23m | -0.001 |
| C8M | B23m | -0.098 |
| C9M | B23m | -0.098 |
| C10M | B23m | 0.060 |
| C11M | B23m | -0.061 |
| C12M | B23m | -0.068 |
| C13M | B23m | -0.077 |
| C14M | B23m | -0.113 |
| C15M | B23m | -0.068 |
| C16M | B23m | -0.010 |
| C17M | B23m | 0.013 |
| C18M | B23m | -0.072 |
| C19M | B23m | -0.079 |
| C20M | B23m | -0.095 |
| C21M | B23m | 0.087 |
| C22M | B23m | -0.039 |
| C23M | B23m | -0.079 |
| C24M | B23m | 0.053 |
| C25M | B23m | -0.047 |
| C26M | B23m | -0.030 |
| C27M | B23m | -0.094 |
| C28M | B23m | -0.054 |
| C29M | B23m | -0.069 |
| C30M | B23m | -0.066 |
| B1m | B23m | 0.061 |
| B2m | B23m | -0.049 |
| B3m | B23m | -0.008 |
| B4m | B23m | -0.055 |
| B5m | B23m | -0.022 |
| B6m | B23m | -0.023 |
| B7m | B23m | -0.128 |
| B8m | B23m | 0.084 |
| B9m | B23m | 0.075 |
| B10m | B23m | -0.043 |
| B11m | B23m | 0.055 |
| B12m | B23m | -0.014 |
| B13m | B23m | -0.005 |
| B14m | B23m | 0.094 |
| B15m | B23m | 0.140 |
| B16m | B23m | 0.021 |
| B17m | B23m | -0.046 |
| B18m | B23m | 0.133 |
| B19m | B23m | -0.097 |
| B20m | B23m | 0.157 |
| B21m | B23m | 0.179 |
| B22m | B23m | 0.082 |
| B1 | B24m | -0.041 |
| B2 | B24m | -0.034 |
| B3 | B24m | -0.116 |
| B4 | B24m | -0.006 |
| B5 | B24m | 0.009 |
| B6 | B24m | 0.059 |
| B7 | B24m | 0.001 |
| B8F | B24m | 0.039 |
| B9F | B24m | 0.170 |
| B10F | B24m | 0.012 |
| B11F | B24m | -0.027 |
| B12F | B24m | 0.017 |
| B13M | B24m | -0.030 |
| B14F | B24m | 0.038 |
| B15M | B24m | -0.059 |
| B16F | B24m | -0.026 |
| B17F | B24m | -0.010 |
| B18M | B24m | -0.010 |
| B19M | B24m | -0.031 |
| B20F | B24m | 0.066 |
| B21F | B24m | -0.039 |
| B22F | B24m | 0.013 |
| B23F | B24m | -0.047 |
| B24F | B24m | 0.049 |
| B25F | B24m | -0.031 |
| B26F | B24m | 0.137 |
| B27F | B24m | 0.001 |
| B28M | B24m | 0.062 |
| B29F | B24m | 0.019 |
| B30F | B24m | -0.001 |
| B31M | B24m | -0.142 |
| B32M | B24m | 0.013 |
| B33M | B24m | 0.019 |
| B34M | B24m | -0.069 |
| B35M | B24m | -0.116 |
| B36M | B24m | 0.013 |
| B37M | B24m | 0.069 |
| C1M | B24m | -0.084 |
| C2M | B24m | -0.010 |
| C3M | B24m | -0.065 |
| C4M | B24m | -0.029 |
| C5M | B24m | -0.022 |
| C6M | B24m | 0.126 |
| C7M | B24m | -0.002 |
| C8M | B24m | 0.006 |
| C9M | B24m | -0.098 |
| C10M | B24m | 0.067 |
| C11M | B24m | -0.092 |
| C12M | B24m | -0.030 |
| C13M | B24m | 0.066 |
| C14M | B24m | 0.007 |
| C15M | B24m | -0.026 |
| C16M | B24m | 0.029 |
| C17M | B24m | 0.003 |
| C18M | B24m | 0.014 |
| C19M | B24m | -0.006 |
| C20M | B24m | -0.036 |
| C21M | B24m | 0.011 |
| C22M | B24m | 0.018 |
| C23M | B24m | -0.013 |
| C24M | B24m | 0.016 |
| C25M | B24m | 0.066 |
| C26M | B24m | 0.018 |
| C27M | B24m | 0.044 |
| C28M | B24m | -0.063 |
| C29M | B24m | -0.058 |
| C30M | B24m | -0.016 |
| B1m | B24m | 0.011 |
| B2m | B24m | -0.084 |
| B3m | B24m | 0.008 |
| B4m | B24m | -0.055 |
| B5m | B24m | -0.063 |
| B6m | B24m | -0.081 |
| B7m | B24m | 0.030 |
| B8m | B24m | 0.144 |
| B9m | B24m | 0.005 |
| B10m | B24m | -0.016 |
| B11m | B24m | 0.051 |
| B12m | B24m | -0.038 |
| B13m | B24m | -0.129 |
| B14m | B24m | -0.012 |
| B15m | B24m | 0.002 |
| B16m | B24m | -0.016 |
| B17m | B24m | 0.011 |
| B18m | B24m | -0.047 |
| B19m | B24m | -0.047 |
| B20m | B24m | -0.062 |
| B21m | B24m | 0.024 |
| B22m | B24m | 0.135 |
| B23m | B24m | 0.075 |
| B1 | B25m | -0.089 |
| B2 | B25m | -0.017 |
| B3 | B25m | -0.058 |
| B4 | B25m | -0.012 |
| B5 | B25m | -0.024 |
| B6 | B25m | -0.054 |
| B7 | B25m | -0.033 |
| B8F | B25m | -0.050 |
| B9F | B25m | -0.088 |
| B10F | B25m | -0.093 |
| B11F | B25m | -0.019 |
| B12F | B25m | -0.053 |
| B13M | B25m | -0.041 |
| B14F | B25m | -0.061 |
| B15M | B25m | -0.013 |
| B16F | B25m | -0.044 |
| B17F | B25m | -0.061 |
| B18M | B25m | -0.059 |
| B19M | B25m | -0.051 |
| B20F | B25m | -0.025 |
| B21F | B25m | -0.017 |
| B22F | B25m | -0.116 |
| B23F | B25m | -0.054 |
| B24F | B25m | -0.048 |
| B25F | B25m | -0.046 |
| B26F | B25m | 0.170 |
| B27F | B25m | 0.002 |
| B28M | B25m | 0.135 |
| B29F | B25m | -0.070 |
| B30F | B25m | -0.054 |
| B31M | B25m | -0.071 |
| B32M | B25m | 0.168 |
| B33M | B25m | -0.037 |
| B34M | B25m | -0.020 |
| B35M | B25m | -0.080 |
| B36M | B25m | -0.093 |
| B37M | B25m | -0.044 |
| C1M | B25m | -0.071 |
| C2M | B25m | -0.069 |
| C3M | B25m | -0.002 |
| C4M | B25m | -0.041 |
| C5M | B25m | -0.059 |
| C6M | B25m | -0.025 |
| C7M | B25m | 0.021 |
| C8M | B25m | -0.025 |
| C9M | B25m | -0.043 |
| C10M | B25m | -0.075 |
| C11M | B25m | 0.001 |
| C12M | B25m | -0.041 |
| C13M | B25m | -0.072 |
| C14M | B25m | -0.033 |
| C15M | B25m | -0.026 |
| C16M | B25m | 0.040 |
| C17M | B25m | -0.056 |
| C18M | B25m | -0.049 |
| C19M | B25m | -0.024 |
| C20M | B25m | -0.001 |
| C21M | B25m | 0.009 |
| C22M | B25m | -0.057 |
| C23M | B25m | -0.041 |
| C24M | B25m | -0.043 |
| C25M | B25m | 0.004 |
| C26M | B25m | 0.051 |
| C27M | B25m | -0.040 |
| C28M | B25m | -0.052 |
| C29M | B25m | -0.022 |
| C30M | B25m | 0.034 |
| B1m | B25m | 0.003 |
| B2m | B25m | 0.111 |
| B3m | B25m | -0.003 |
| B4m | B25m | 0.140 |
| B5m | B25m | -0.022 |
| B6m | B25m | 0.269 |
| B7m | B25m | -0.011 |
| B8m | B25m | -0.033 |
| B9m | B25m | 0.011 |
| B10m | B25m | 0.059 |
| B11m | B25m | -0.025 |
| B12m | B25m | 0.030 |
| B13m | B25m | -0.021 |
| B14m | B25m | -0.031 |
| B15m | B25m | 0.025 |
| B16m | B25m | 0.050 |
| B17m | B25m | 0.007 |
| B18m | B25m | 0.046 |
| B19m | B25m | -0.043 |
| B20m | B25m | -0.079 |
| B21m | B25m | -0.017 |
| B22m | B25m | -0.005 |
| B23m | B25m | -0.013 |
| B24m | B25m | -0.009 |
| B1 | B26m | -0.059 |
| B2 | B26m | -0.060 |
| B3 | B26m | -0.062 |
| B4 | B26m | -0.002 |
| B5 | B26m | -0.006 |
| B6 | B26m | -0.064 |
| B7 | B26m | 0.019 |
| B8F | B26m | -0.012 |
| B9F | B26m | -0.042 |
| B10F | B26m | -0.022 |
| B11F | B26m | -0.026 |
| B12F | B26m | -0.039 |
| B13M | B26m | -0.053 |
| B14F | B26m | -0.043 |
| B15M | B26m | -0.085 |
| B16F | B26m | 0.030 |
| B17F | B26m | -0.069 |
| B18M | B26m | 0.166 |
| B19M | B26m | 0.039 |
| B20F | B26m | -0.064 |
| B21F | B26m | -0.050 |
| B22F | B26m | 0.052 |
| B23F | B26m | -0.073 |
| B24F | B26m | -0.056 |
| B25F | B26m | -0.095 |
| B26F | B26m | -0.075 |
| B27F | B26m | -0.051 |
| B28M | B26m | -0.080 |
| B29F | B26m | -0.058 |
| B30F | B26m | -0.071 |
| B31M | B26m | -0.076 |
| B32M | B26m | 0.130 |
| B33M | B26m | 0.123 |
| B34M | B26m | -0.080 |
| B35M | B26m | 0.025 |
| B36M | B26m | -0.103 |
| B37M | B26m | -0.013 |
| C1M | B26m | -0.067 |
| C2M | B26m | -0.038 |
| C3M | B26m | -0.048 |
| C4M | B26m | -0.083 |
| C5M | B26m | -0.003 |
| C6M | B26m | 0.034 |
| C7M | B26m | 0.001 |
| C8M | B26m | -0.078 |
| C9M | B26m | -0.046 |
| C10M | B26m | -0.016 |
| C11M | B26m | -0.028 |
| C12M | B26m | 0.080 |
| C13M | B26m | -0.011 |
| C14M | B26m | -0.054 |
| C15M | B26m | -0.054 |
| C16M | B26m | -0.071 |
| C17M | B26m | 0.016 |
| C18M | B26m | -0.025 |
| C19M | B26m | -0.032 |
| C20M | B26m | 0.023 |
| C21M | B26m | -0.044 |
| C22M | B26m | -0.014 |
| C23M | B26m | -0.084 |
| C24M | B26m | 0.017 |
| C25M | B26m | -0.007 |
| C26M | B26m | -0.078 |
| C27M | B26m | -0.033 |
| C28M | B26m | -0.070 |
| C29M | B26m | -0.063 |
| C30M | B26m | 0.037 |
| B1m | B26m | 0.051 |
| B2m | B26m | 0.002 |
| B3m | B26m | 0.007 |
| B4m | B26m | -0.003 |
| B5m | B26m | -0.083 |
| B6m | B26m | 0.204 |
| B7m | B26m | -0.083 |
| B8m | B26m | 0.070 |
| B9m | B26m | -0.015 |
| B10m | B26m | 0.211 |
| B11m | B26m | -0.054 |
| B12m | B26m | 0.057 |
| B13m | B26m | 0.039 |
| B14m | B26m | 0.029 |
| B15m | B26m | 0.075 |
| B16m | B26m | -0.066 |
| B17m | B26m | -0.012 |
| B18m | B26m | 0.060 |
| B19m | B26m | -0.035 |
| B20m | B26m | -0.009 |
| B21m | B26m | -0.094 |
| B22m | B26m | 0.031 |
| B23m | B26m | 0.092 |
| B24m | B26m | -0.015 |
| B25m | B26m | 0.231 |
| B1 | B27m | 0.022 |
| B2 | B27m | -0.136 |
| B3 | B27m | -0.100 |
| B4 | B27m | 0.039 |
| B5 | B27m | -0.007 |
| B6 | B27m | -0.014 |
| B7 | B27m | -0.024 |
| B8F | B27m | -0.053 |
| B9F | B27m | -0.016 |
| B10F | B27m | -0.073 |
| B11F | B27m | -0.064 |
| B12F | B27m | 0.090 |
| B13M | B27m | -0.004 |
| B14F | B27m | -0.070 |
| B15M | B27m | -0.004 |
| B16F | B27m | -0.003 |
| B17F | B27m | 0.004 |
| B18M | B27m | -0.046 |
| B19M | B27m | -0.028 |
| B20F | B27m | 0.077 |
| B21F | B27m | 0.017 |
| B22F | B27m | -0.116 |
| B23F | B27m | -0.088 |
| B24F | B27m | 0.004 |
| B25F | B27m | 0.113 |
| B26F | B27m | -0.029 |
| B27F | B27m | 0.084 |
| B28M | B27m | 0.037 |
| B29F | B27m | -0.065 |
| B30F | B27m | -0.016 |
| B31M | B27m | 0.007 |
| B32M | B27m | -0.110 |
| B33M | B27m | -0.022 |
| B34M | B27m | -0.055 |
| B35M | B27m | -0.051 |
| B36M | B27m | -0.004 |
| B37M | B27m | -0.014 |
| C1M | B27m | -0.102 |
| C2M | B27m | -0.068 |
| C3M | B27m | -0.003 |
| C4M | B27m | -0.056 |
| C5M | B27m | 0.100 |
| C6M | B27m | 0.027 |
| C7M | B27m | -0.011 |
| C8M | B27m | 0.010 |
| C9M | B27m | -0.110 |
| C10M | B27m | 0.077 |
| C11M | B27m | -0.053 |
| C12M | B27m | -0.031 |
| C13M | B27m | -0.111 |
| C14M | B27m | -0.077 |
| C15M | B27m | -0.079 |
| C16M | B27m | -0.010 |
| C17M | B27m | -0.048 |
| C18M | B27m | -0.058 |
| C19M | B27m | -0.104 |
| C20M | B27m | -0.060 |
| C21M | B27m | -0.067 |
| C22M | B27m | -0.063 |
| C23M | B27m | 0.011 |
| C24M | B27m | 0.080 |
| C25M | B27m | 0.003 |
| C26M | B27m | -0.076 |
| C27M | B27m | -0.046 |
| C28M | B27m | -0.089 |
| C29M | B27m | -0.068 |
| C30M | B27m | -0.080 |
| B1m | B27m | -0.084 |
| B2m | B27m | -0.041 |
| B3m | B27m | -0.069 |
| B4m | B27m | 0.041 |
| B5m | B27m | 0.051 |
| B6m | B27m | 0.203 |
| B7m | B27m | -0.004 |
| B8m | B27m | -0.004 |
| B9m | B27m | -0.018 |
| B10m | B27m | -0.027 |
| B11m | B27m | 0.054 |
| B12m | B27m | 0.028 |
| B13m | B27m | 0.029 |
| B14m | B27m | 0.057 |
| B15m | B27m | 0.107 |
| B16m | B27m | 0.041 |
| B17m | B27m | -0.053 |
| B18m | B27m | -0.036 |
| B19m | B27m | -0.018 |
| B20m | B27m | 0.089 |
| B21m | B27m | 0.159 |
| B22m | B27m | -0.027 |
| B23m | B27m | 0.178 |
| B24m | B27m | 0.053 |
| B25m | B27m | 0.122 |
| B26m | B27m | 0.067 |
| B1 | B28m | -0.110 |
| B2 | B28m | -0.098 |
| B3 | B28m | -0.049 |
| B4 | B28m | 0.029 |
| B5 | B28m | 0.025 |
| B6 | B28m | -0.032 |
| B7 | B28m | 0.007 |
| B8F | B28m | -0.060 |
| B9F | B28m | -0.028 |
| B10F | B28m | -0.076 |
| B11F | B28m | -0.033 |
| B12F | B28m | 0.078 |
| B13M | B28m | -0.034 |
| B14F | B28m | -0.085 |
| B15M | B28m | 0.040 |
| B16F | B28m | -0.043 |
| B17F | B28m | 0.021 |
| B18M | B28m | -0.040 |
| B19M | B28m | -0.060 |
| B20F | B28m | -0.038 |
| B21F | B28m | -0.092 |
| B22F | B28m | 0.003 |
| B23F | B28m | -0.087 |
| B24F | B28m | -0.046 |
| B25F | B28m | 0.047 |
| B26F | B28m | -0.086 |
| B27F | B28m | -0.073 |
| B28M | B28m | 0.002 |
| B29F | B28m | -0.009 |
| B30F | B28m | -0.072 |
| B31M | B28m | -0.090 |
| B32M | B28m | -0.041 |
| B33M | B28m | 0.051 |
| B34M | B28m | -0.070 |
| B35M | B28m | 0.025 |
| B36M | B28m | -0.085 |
| B37M | B28m | -0.089 |
| C1M | B28m | -0.086 |
| C2M | B28m | 0.090 |
| C3M | B28m | -0.064 |
| C4M | B28m | -0.121 |
| C5M | B28m | -0.095 |
| C6M | B28m | 0.108 |
| C7M | B28m | -0.051 |
| C8M | B28m | -0.079 |
| C9M | B28m | -0.068 |
| C10M | B28m | -0.053 |
| C11M | B28m | -0.003 |
| C12M | B28m | 0.125 |
| C13M | B28m | 0.056 |
| C14M | B28m | -0.077 |
| C15M | B28m | 0.022 |
| C16M | B28m | -0.043 |
| C17M | B28m | -0.021 |
| C18M | B28m | 0.075 |
| C19M | B28m | -0.063 |
| C20M | B28m | -0.020 |
| C21M | B28m | -0.076 |
| C22M | B28m | -0.038 |
| C23M | B28m | 0.018 |
| C24M | B28m | 0.022 |
| C25M | B28m | -0.010 |
| C26M | B28m | 0.040 |
| C27M | B28m | -0.128 |
| C28M | B28m | -0.099 |
| C29M | B28m | -0.058 |
| C30M | B28m | -0.042 |
| B1m | B28m | 0.043 |
| B2m | B28m | 0.063 |
| B3m | B28m | 0.064 |
| B4m | B28m | 0.088 |
| B5m | B28m | 0.003 |
| B6m | B28m | 0.158 |
| B7m | B28m | -0.055 |
| B8m | B28m | -0.076 |
| B9m | B28m | 0.044 |
| B10m | B28m | 0.013 |
| B11m | B28m | -0.088 |
| B12m | B28m | 0.042 |
| B13m | B28m | 0.104 |
| B14m | B28m | 0.182 |
| B15m | B28m | 0.096 |
| B16m | B28m | 0.023 |
| B17m | B28m | -0.069 |
| B18m | B28m | 0.151 |
| B19m | B28m | 0.049 |
| B20m | B28m | -0.003 |
| B21m | B28m | -0.023 |
| B22m | B28m | 0.007 |
| B23m | B28m | 0.115 |
| B24m | B28m | -0.055 |
| B25m | B28m | 0.155 |
| B26m | B28m | 0.184 |
| B27m | B28m | 0.089 |
| B1 | B29m | 0.152 |
| B2 | B29m | 0.050 |
| B3 | B29m | 0.045 |
| B4 | B29m | -0.066 |
| B5 | B29m | -0.070 |
| B6 | B29m | -0.077 |
| B7 | B29m | -0.074 |
| B8F | B29m | -0.065 |
| B9F | B29m | -0.049 |
| B10F | B29m | -0.132 |
| B11F | B29m | -0.065 |
| B12F | B29m | -0.030 |
| B13M | B29m | -0.095 |
| B14F | B29m | -0.054 |
| B15M | B29m | -0.152 |
| B16F | B29m | -0.053 |
| B17F | B29m | -0.051 |
| B18M | B29m | 0.006 |
| B19M | B29m | -0.094 |
| B20F | B29m | -0.003 |
| B21F | B29m | -0.065 |
| B22F | B29m | -0.029 |
| B23F | B29m | 0.302 |
| B24F | B29m | -0.048 |
| B25F | B29m | -0.036 |
| B26F | B29m | -0.061 |
| B27F | B29m | -0.046 |
| B28M | B29m | -0.055 |
| B29F | B29m | 0.268 |
| B30F | B29m | -0.074 |
| B31M | B29m | -0.060 |
| B32M | B29m | -0.088 |
| B33M | B29m | -0.026 |
| B34M | B29m | -0.065 |
| B35M | B29m | -0.104 |
| B36M | B29m | 0.038 |
| B37M | B29m | -0.112 |
| C1M | B29m | -0.053 |
| C2M | B29m | -0.019 |
| C3M | B29m | 0.000 |
| C4M | B29m | -0.016 |
| C5M | B29m | -0.040 |
| C6M | B29m | -0.086 |
| C7M | B29m | -0.047 |
| C8M | B29m | -0.070 |
| C9M | B29m | -0.088 |
| C10M | B29m | -0.040 |
| C11M | B29m | -0.055 |
| C12M | B29m | -0.050 |
| C13M | B29m | -0.120 |
| C14M | B29m | 0.184 |
| C15M | B29m | -0.049 |
| C16M | B29m | -0.013 |
| C17M | B29m | 0.023 |
| C18M | B29m | -0.110 |
| C19M | B29m | -0.004 |
| C20M | B29m | 0.236 |
| C21M | B29m | -0.072 |
| C22M | B29m | 0.081 |
| C23M | B29m | -0.045 |
| C24M | B29m | 0.008 |
| C25M | B29m | 0.025 |
| C26M | B29m | -0.029 |
| C27M | B29m | 0.156 |
| C28M | B29m | -0.032 |
| C29M | B29m | -0.112 |
| C30M | B29m | -0.053 |
| B1m | B29m | -0.084 |
| B2m | B29m | 0.006 |
| B3m | B29m | -0.008 |
| B4m | B29m | -0.009 |
| B5m | B29m | -0.112 |
| B6m | B29m | -0.046 |
| B7m | B29m | -0.003 |
| B8m | B29m | -0.041 |
| B9m | B29m | -0.033 |
| B10m | B29m | -0.091 |
| B11m | B29m | -0.053 |
| B12m | B29m | -0.037 |
| B13m | B29m | -0.052 |
| B14m | B29m | -0.023 |
| B15m | B29m | -0.013 |
| B16m | B29m | -0.083 |
| B17m | B29m | -0.036 |
| B18m | B29m | -0.114 |
| B19m | B29m | 0.034 |
| B20m | B29m | -0.098 |
| B21m | B29m | -0.064 |
| B22m | B29m | -0.023 |
| B23m | B29m | -0.018 |
| B24m | B29m | 0.003 |
| B25m | B29m | 0.070 |
| B26m | B29m | -0.021 |
| B27m | B29m | 0.096 |
| B28m | B29m | 0.004 |
| B1 | B30m | 0.130 |
| B2 | B30m | 0.021 |
| B3 | B30m | -0.043 |
| B4 | B30m | 0.094 |
| B5 | B30m | 0.027 |
| B6 | B30m | -0.011 |
| B7 | B30m | 0.002 |
| B8F | B30m | -0.096 |
| B9F | B30m | -0.078 |
| B10F | B30m | -0.106 |
| B11F | B30m | -0.004 |
| B12F | B30m | -0.021 |
| B13M | B30m | -0.052 |
| B14F | B30m | -0.007 |
| B15M | B30m | -0.048 |
| B16F | B30m | 0.042 |
| B17F | B30m | -0.022 |
| B18M | B30m | -0.064 |
| B19M | B30m | -0.003 |
| B20F | B30m | 0.030 |
| B21F | B30m | 0.100 |
| B22F | B30m | -0.056 |
| B23F | B30m | 0.138 |
| B24F | B30m | -0.086 |
| B25F | B30m | 0.052 |
| B26F | B30m | 0.010 |
| B27F | B30m | 0.115 |
| B28M | B30m | 0.032 |
| B29F | B30m | 0.056 |
| B30F | B30m | -0.044 |
| B31M | B30m | -0.004 |
| B32M | B30m | -0.027 |
| B33M | B30m | -0.061 |
| B34M | B30m | -0.040 |
| B35M | B30m | -0.077 |
| B36M | B30m | -0.035 |
| B37M | B30m | -0.082 |
| C1M | B30m | 0.004 |
| C2M | B30m | -0.118 |
| C3M | B30m | 0.034 |
| C4M | B30m | -0.013 |
| C5M | B30m | -0.038 |
| C6M | B30m | -0.019 |
| C7M | B30m | -0.028 |
| C8M | B30m | -0.082 |
| C9M | B30m | -0.064 |
| C10M | B30m | 0.023 |
| C11M | B30m | -0.017 |
| C12M | B30m | 0.050 |
| C13M | B30m | -0.004 |
| C14M | B30m | -0.003 |
| C15M | B30m | -0.040 |
| C16M | B30m | 0.095 |
| C17M | B30m | -0.055 |
| C18M | B30m | -0.081 |
| C19M | B30m | -0.128 |
| C20M | B30m | -0.074 |
| C21M | B30m | -0.036 |
| C22M | B30m | -0.006 |
| C23M | B30m | -0.056 |
| C24M | B30m | -0.011 |
| C25M | B30m | 0.019 |
| C26M | B30m | -0.040 |
| C27M | B30m | -0.039 |
| C28M | B30m | -0.058 |
| C29M | B30m | -0.077 |
| C30M | B30m | -0.090 |
| B1m | B30m | -0.049 |
| B2m | B30m | -0.040 |
| B3m | B30m | -0.019 |
| B4m | B30m | -0.086 |
| B5m | B30m | -0.023 |
| B6m | B30m | 0.077 |
| B7m | B30m | -0.004 |
| B8m | B30m | -0.024 |
| B9m | B30m | -0.030 |
| B10m | B30m | -0.049 |
| B11m | B30m | 0.082 |
| B12m | B30m | -0.034 |
| B13m | B30m | -0.076 |
| B14m | B30m | -0.038 |
| B15m | B30m | -0.056 |
| B16m | B30m | -0.014 |
| B17m | B30m | -0.086 |
| B18m | B30m | 0.090 |
| B19m | B30m | 0.027 |
| B20m | B30m | 0.087 |
| B21m | B30m | 0.047 |
| B22m | B30m | 0.011 |
| B23m | B30m | 0.206 |
| B24m | B30m | -0.030 |
| B25m | B30m | 0.098 |
| B26m | B30m | 0.070 |
| B27m | B30m | 0.206 |
| B28m | B30m | 0.081 |
| B29m | B30m | 0.154 |
