# Supplementary material for: Questioning inbreeding: Could outbreeding affect productivity in the North African catfish in Thailand?
Source: PLoS One. 2024 May 6;19(5):e0302584. doi: 10.1371/journal.pone.0302584 (PMC11073742; doi:10.1371/journal.pone.0302584)
Supplement: S13 Table — (DOCX) [file pone.0302584.s013.docx]

**S13 Table.** Pairwise comparison of genetic relatedness (*r*) values for 31 individuals from the Nakhon Nayok population.

| **Sample 1** | **Sample 2** | ***r*** |
| --- | --- | --- |
| N1F | N2M | -0.031 |
| N1F | N3F | -0.038 |
| N2M | N3F | -0.070 |
| N1F | N4M | -0.061 |
| N2M | N4M | 0.056 |
| N3F | N4M | -0.014 |
| N1F | N5M | -0.042 |
| N2M | N5M | -0.009 |
| N3F | N5M | -0.106 |
| N4M | N5M | 0.003 |
| N1F | N6M | -0.021 |
| N2M | N6M | -0.067 |
| N3F | N6M | -0.094 |
| N4M | N6M | 0.048 |
| N5M | N6M | -0.035 |
| N1F | N7F | -0.055 |
| N2M | N7F | 0.048 |
| N3F | N7F | -0.067 |
| N4M | N7F | -0.036 |
| N5M | N7F | -0.099 |
| N6M | N7F | 0.058 |
| N1F | N8F | 0.013 |
| N2M | N8F | -0.011 |
| N3F | N8F | 0.001 |
| N4M | N8F | -0.124 |
| N5M | N8F | -0.133 |
| N6M | N8F | -0.033 |
| N7F | N8F | -0.008 |
| N1F | N9F | -0.054 |
| N2M | N9F | -0.022 |
| N3F | N9F | 0.013 |
| N4M | N9F | 0.079 |
| N5M | N9F | -0.034 |
| N6M | N9F | -0.010 |
| N7F | N9F | 0.103 |
| N8F | N9F | 0.140 |
| N1F | N10F | -0.034 |
| N2M | N10F | 0.023 |
| N3F | N10F | -0.112 |
| N4M | N10F | 0.007 |
| N5M | N10F | -0.109 |
| N6M | N10F | -0.058 |
| N7F | N10F | -0.080 |
| N8F | N10F | 0.148 |
| N9F | N10F | -0.081 |
| N1F | N11M | 0.135 |
| N2M | N11M | -0.017 |
| N3F | N11M | 0.023 |
| N4M | N11M | -0.083 |
| N5M | N11M | -0.072 |
| N6M | N11M | -0.038 |
| N7F | N11M | 0.155 |
| N8F | N11M | -0.010 |
| N9F | N11M | -0.067 |
| N10F | N11M | -0.024 |
| N1F | N12M | -0.014 |
| N2M | N12M | -0.008 |
| N3F | N12M | -0.147 |
| N4M | N12M | 0.067 |
| N5M | N12M | 0.000 |
| N6M | N12M | -0.068 |
| N7F | N12M | 0.071 |
| N8F | N12M | -0.106 |
| N9F | N12M | 0.044 |
| N10F | N12M | -0.028 |
| N11M | N12M | -0.022 |
| N1F | N13F | 0.006 |
| N2M | N13F | 0.000 |
| N3F | N13F | -0.072 |
| N4M | N13F | -0.044 |
| N5M | N13F | -0.044 |
| N6M | N13F | -0.049 |
| N7F | N13F | -0.101 |
| N8F | N13F | 0.026 |
| N9F | N13F | -0.003 |
| N10F | N13F | -0.030 |
| N11M | N13F | -0.053 |
| N12M | N13F | -0.046 |
| N1F | N15F | -0.013 |
| N2M | N15F | 0.067 |
| N3F | N15F | -0.075 |
| N4M | N15F | -0.028 |
| N5M | N15F | -0.011 |
| N6M | N15F | -0.093 |
| N7F | N15F | -0.022 |
| N8F | N15F | 0.059 |
| N9F | N15F | 0.068 |
| N10F | N15F | -0.046 |
| N11M | N15F | 0.046 |
| N12M | N15F | 0.005 |
| N13F | N15F | 0.059 |
| N1F | N16M | 0.055 |
| N2M | N16M | -0.021 |
| N3F | N16M | -0.041 |
| N4M | N16M | -0.116 |
| N5M | N16M | -0.122 |
| N6M | N16M | -0.048 |
| N7F | N16M | 0.009 |
| N8F | N16M | 0.060 |
| N9F | N16M | -0.056 |
| N10F | N16M | 0.040 |
| N11M | N16M | -0.021 |
| N12M | N16M | -0.046 |
| N13F | N16M | -0.009 |
| N15F | N16M | 0.240 |
| N1F | N17F | -0.070 |
| N2M | N17F | -0.014 |
| N3F | N17F | -0.082 |
| N4M | N17F | -0.064 |
| N5M | N17F | 0.126 |
| N6M | N17F | -0.036 |
| N7F | N17F | 0.008 |
| N8F | N17F | -0.165 |
| N9F | N17F | -0.032 |
| N10F | N17F | 0.136 |
| N11M | N17F | -0.045 |
| N12M | N17F | 0.073 |
| N13F | N17F | -0.040 |
| N15F | N17F | -0.069 |
| N16M | N17F | -0.033 |
| N1F | N18F | -0.099 |
| N2M | N18F | 0.084 |
| N3F | N18F | 0.058 |
| N4M | N18F | -0.068 |
| N5M | N18F | -0.135 |
| N6M | N18F | -0.056 |
| N7F | N18F | -0.056 |
| N8F | N18F | -0.171 |
| N9F | N18F | 0.011 |
| N10F | N18F | -0.062 |
| N11M | N18F | -0.085 |
| N12M | N18F | 0.009 |
| N13F | N18F | -0.003 |
| N15F | N18F | -0.033 |
| N16M | N18F | -0.019 |
| N17F | N18F | -0.041 |
| N1F | N19F | -0.103 |
| N2M | N19F | -0.056 |
| N3F | N19F | 0.063 |
| N4M | N19F | -0.031 |
| N5M | N19F | -0.145 |
| N6M | N19F | 0.056 |
| N7F | N19F | 0.030 |
| N8F | N19F | -0.141 |
| N9F | N19F | 0.108 |
| N10F | N19F | -0.092 |
| N11M | N19F | -0.078 |
| N12M | N19F | -0.040 |
| N13F | N19F | -0.070 |
| N15F | N19F | -0.091 |
| N16M | N19F | -0.031 |
| N17F | N19F | 0.013 |
| N18F | N19F | 0.180 |
| N1F | N20F | -0.047 |
| N2M | N20F | -0.030 |
| N3F | N20F | -0.028 |
| N4M | N20F | 0.139 |
| N5M | N20F | 0.022 |
| N6M | N20F | -0.033 |
| N7F | N20F | -0.071 |
| N8F | N20F | -0.036 |
| N9F | N20F | -0.022 |
| N10F | N20F | -0.032 |
| N11M | N20F | -0.004 |
| N12M | N20F | -0.075 |
| N13F | N20F | -0.016 |
| N15F | N20F | 0.002 |
| N16M | N20F | -0.044 |
| N17F | N20F | -0.047 |
| N18F | N20F | -0.016 |
| N19F | N20F | 0.045 |
| N1F | N21F | -0.025 |
| N2M | N21F | 0.017 |
| N3F | N21F | 0.067 |
| N4M | N21F | -0.069 |
| N5M | N21F | 0.079 |
| N6M | N21F | -0.078 |
| N7F | N21F | -0.104 |
| N8F | N21F | -0.041 |
| N9F | N21F | -0.123 |
| N10F | N21F | -0.100 |
| N11M | N21F | -0.015 |
| N12M | N21F | -0.074 |
| N13F | N21F | 0.004 |
| N15F | N21F | -0.101 |
| N16M | N21F | -0.027 |
| N17F | N21F | -0.061 |
| N18F | N21F | 0.099 |
| N19F | N21F | 0.013 |
| N20F | N21F | -0.049 |
| N1F | N22F | 0.092 |
| N2M | N22F | -0.039 |
| N3F | N22F | 0.020 |
| N4M | N22F | 0.006 |
| N5M | N22F | -0.061 |
| N6M | N22F | 0.022 |
| N7F | N22F | -0.012 |
| N8F | N22F | -0.090 |
| N9F | N22F | -0.019 |
| N10F | N22F | -0.022 |
| N11M | N22F | 0.034 |
| N12M | N22F | -0.015 |
| N13F | N22F | -0.003 |
| N15F | N22F | -0.042 |
| N16M | N22F | -0.044 |
| N17F | N22F | -0.022 |
| N18F | N22F | -0.018 |
| N19F | N22F | -0.045 |
| N20F | N22F | 0.004 |
| N21F | N22F | 0.029 |
| N1F | N23F | -0.098 |
| N2M | N23F | -0.085 |
| N3F | N23F | -0.093 |
| N4M | N23F | -0.062 |
| N5M | N23F | 0.010 |
| N6M | N23F | 0.003 |
| N7F | N23F | -0.084 |
| N8F | N23F | 0.033 |
| N9F | N23F | -0.036 |
| N10F | N23F | -0.046 |
| N11M | N23F | -0.041 |
| N12M | N23F | -0.069 |
| N13F | N23F | -0.060 |
| N15F | N23F | -0.130 |
| N16M | N23F | -0.069 |
| N17F | N23F | -0.033 |
| N18F | N23F | -0.068 |
| N19F | N23F | -0.074 |
| N20F | N23F | -0.041 |
| N21F | N23F | -0.042 |
| N22F | N23F | 0.019 |
| N1F | N24F | 0.021 |
| N2M | N24F | -0.036 |
| N3F | N24F | -0.046 |
| N4M | N24F | -0.109 |
| N5M | N24F | -0.012 |
| N6M | N24F | -0.128 |
| N7F | N24F | 0.020 |
| N8F | N24F | 0.033 |
| N9F | N24F | -0.087 |
| N10F | N24F | -0.053 |
| N11M | N24F | -0.020 |
| N12M | N24F | -0.004 |
| N13F | N24F | 0.006 |
| N15F | N24F | -0.005 |
| N16M | N24F | -0.034 |
| N17F | N24F | -0.057 |
| N18F | N24F | -0.032 |
| N19F | N24F | -0.017 |
| N20F | N24F | -0.012 |
| N21F | N24F | 0.002 |
| N22F | N24F | -0.014 |
| N23F | N24F | 0.024 |
| N1F | N25M | 0.012 |
| N2M | N25M | 0.049 |
| N3F | N25M | 0.055 |
| N4M | N25M | -0.028 |
| N5M | N25M | -0.106 |
| N6M | N25M | 0.025 |
| N7F | N25M | -0.011 |
| N8F | N25M | -0.072 |
| N9F | N25M | -0.107 |
| N10F | N25M | -0.036 |
| N11M | N25M | 0.028 |
| N12M | N25M | -0.066 |
| N13F | N25M | 0.024 |
| N15F | N25M | -0.051 |
| N16M | N25M | -0.077 |
| N17F | N25M | -0.049 |
| N18F | N25M | 0.041 |
| N19F | N25M | 0.020 |
| N20F | N25M | 0.021 |
| N21F | N25M | 0.006 |
| N22F | N25M | 0.114 |
| N23F | N25M | -0.008 |
| N24F | N25M | -0.031 |
| N1F | N26M | -0.034 |
| N2M | N26M | -0.065 |
| N3F | N26M | 0.001 |
| N4M | N26M | 0.041 |
| N5M | N26M | -0.054 |
| N6M | N26M | 0.200 |
| N7F | N26M | -0.022 |
| N8F | N26M | -0.025 |
| N9F | N26M | -0.031 |
| N10F | N26M | -0.082 |
| N11M | N26M | -0.055 |
| N12M | N26M | -0.065 |
| N13F | N26M | -0.046 |
| N15F | N26M | -0.093 |
| N16M | N26M | -0.030 |
| N17F | N26M | -0.069 |
| N18F | N26M | 0.022 |
| N19F | N26M | 0.059 |
| N20F | N26M | 0.076 |
| N21F | N26M | -0.025 |
| N22F | N26M | -0.013 |
| N23F | N26M | 0.026 |
| N24F | N26M | -0.054 |
| N25M | N26M | -0.033 |
| N1F | N27M | 0.071 |
| N2M | N27M | -0.047 |
| N3F | N27M | -0.005 |
| N4M | N27M | -0.033 |
| N5M | N27M | 0.116 |
| N6M | N27M | -0.047 |
| N7F | N27M | -0.114 |
| N8F | N27M | -0.148 |
| N9F | N27M | -0.122 |
| N10F | N27M | 0.015 |
| N11M | N27M | -0.088 |
| N12M | N27M | -0.041 |
| N13F | N27M | -0.052 |
| N15F | N27M | -0.065 |
| N16M | N27M | -0.028 |
| N17F | N27M | 0.024 |
| N18F | N27M | 0.225 |
| N19F | N27M | 0.010 |
| N20F | N27M | -0.116 |
| N21F | N27M | 0.166 |
| N22F | N27M | -0.008 |
| N23F | N27M | -0.022 |
| N24F | N27M | 0.021 |
| N25M | N27M | -0.084 |
| N26M | N27M | -0.022 |
| N1F | N28M | -0.021 |
| N2M | N28M | -0.048 |
| N3F | N28M | -0.010 |
| N4M | N28M | -0.121 |
| N5M | N28M | -0.037 |
| N6M | N28M | -0.074 |
| N7F | N28M | -0.019 |
| N8F | N28M | -0.060 |
| N9F | N28M | -0.158 |
| N10F | N28M | 0.167 |
| N11M | N28M | 0.041 |
| N12M | N28M | 0.093 |
| N13F | N28M | -0.076 |
| N15F | N28M | -0.048 |
| N16M | N28M | -0.002 |
| N17F | N28M | 0.177 |
| N18F | N28M | -0.032 |
| N19F | N28M | -0.032 |
| N20F | N28M | -0.033 |
| N21F | N28M | 0.016 |
| N22F | N28M | -0.032 |
| N23F | N28M | -0.141 |
| N24F | N28M | -0.048 |
| N25M | N28M | -0.003 |
| N26M | N28M | -0.051 |
| N27M | N28M | 0.060 |
| N1F | N29M | -0.078 |
| N2M | N29M | -0.030 |
| N3F | N29M | -0.002 |
| N4M | N29M | -0.164 |
| N5M | N29M | -0.088 |
| N6M | N29M | 0.031 |
| N7F | N29M | 0.061 |
| N8F | N29M | 0.009 |
| N9F | N29M | -0.044 |
| N10F | N29M | -0.107 |
| N11M | N29M | -0.064 |
| N12M | N29M | -0.113 |
| N13F | N29M | -0.057 |
| N15F | N29M | -0.087 |
| N16M | N29M | -0.081 |
| N17F | N29M | -0.086 |
| N18F | N29M | -0.031 |
| N19F | N29M | 0.044 |
| N20F | N29M | -0.023 |
| N21F | N29M | -0.056 |
| N22F | N29M | -0.025 |
| N23F | N29M | -0.060 |
| N24F | N29M | 0.054 |
| N25M | N29M | 0.029 |
| N26M | N29M | -0.003 |
| N27M | N29M | -0.047 |
| N28M | N29M | -0.036 |
| N1F | N30M | -0.056 |
| N2M | N30M | -0.096 |
| N3F | N30M | -0.025 |
| N4M | N30M | -0.013 |
| N5M | N30M | -0.020 |
| N6M | N30M | -0.049 |
| N7F | N30M | -0.100 |
| N8F | N30M | 0.009 |
| N9F | N30M | -0.013 |
| N10F | N30M | -0.072 |
| N11M | N30M | -0.077 |
| N12M | N30M | -0.006 |
| N13F | N30M | 0.122 |
| N15F | N30M | -0.037 |
| N16M | N30M | -0.069 |
| N17F | N30M | -0.097 |
| N18F | N30M | -0.015 |
| N19F | N30M | -0.025 |
| N20F | N30M | 0.054 |
| N21F | N30M | -0.097 |
| N22F | N30M | -0.023 |
| N23F | N30M | 0.127 |
| N24F | N30M | 0.076 |
| N25M | N30M | 0.002 |
| N26M | N30M | -0.004 |
| N27M | N30M | -0.026 |
| N28M | N30M | -0.024 |
| N29M | N30M | 0.009 |
| N1F | N31M | 0.042 |
| N2M | N31M | -0.010 |
| N3F | N31M | -0.033 |
| N4M | N31M | 0.159 |
| N5M | N31M | -0.082 |
| N6M | N31M | -0.006 |
| N7F | N31M | -0.001 |
| N8F | N31M | -0.105 |
| N9F | N31M | 0.019 |
| N10F | N31M | -0.069 |
| N11M | N31M | 0.070 |
| N12M | N31M | -0.014 |
| N13F | N31M | -0.007 |
| N15F | N31M | -0.048 |
| N16M | N31M | 0.029 |
| N17F | N31M | 0.002 |
| N18F | N31M | -0.093 |
| N19F | N31M | -0.090 |
| N20F | N31M | 0.023 |
| N21F | N31M | -0.011 |
| N22F | N31M | -0.035 |
| N23F | N31M | -0.066 |
| N24F | N31M | -0.032 |
| N25M | N31M | -0.053 |
| N26M | N31M | 0.027 |
| N27M | N31M | -0.086 |
| N28M | N31M | -0.098 |
| N29M | N31M | -0.081 |
| N30M | N31M | -0.036 |
| N1F | N32M | -0.035 |
| N2M | N32M | -0.067 |
| N3F | N32M | -0.016 |
| N4M | N32M | -0.048 |
| N5M | N32M | -0.101 |
| N6M | N32M | -0.079 |
| N7F | N32M | -0.048 |
| N8F | N32M | 0.211 |
| N9F | N32M | -0.102 |
| N10F | N32M | 0.115 |
| N11M | N32M | -0.035 |
| N12M | N32M | -0.046 |
| N13F | N32M | -0.065 |
| N15F | N32M | -0.008 |
| N16M | N32M | 0.010 |
| N17F | N32M | -0.070 |
| N18F | N32M | -0.097 |
| N19F | N32M | -0.072 |
| N20F | N32M | -0.089 |
| N21F | N32M | -0.012 |
| N22F | N32M | -0.069 |
| N23F | N32M | -0.050 |
| N24F | N32M | 0.095 |
| N25M | N32M | -0.066 |
| N26M | N32M | -0.046 |
| N27M | N32M | -0.112 |
| N28M | N32M | -0.039 |
| N29M | N32M | 0.097 |
| N30M | N32M | -0.005 |
| N31M | N32M | -0.084 |
