# Supplementary material for: Questioning inbreeding: Could outbreeding affect productivity in the North African catfish in Thailand?
Source: PLoS One. 2024 May 6;19(5):e0302584. doi: 10.1371/journal.pone.0302584 (PMC11073742; doi:10.1371/journal.pone.0302584)
Supplement: S14 Table — The numbers indicate p-values with 110 permutations. (DOCX) [file pone.0302584.s014.docx]

**S14 Table.** Pairwise comparison of genetic differentiation (*F*_ST_), *F*_ST_^ENA^ values with ENA correction for null alleles, and *R*_ST_ values between three populations. The numbers indicate *p*-values with 110 permutations.

| **Combination^§^** | ***F*_ST_** | ***F*_ST_^ENA^** | ***R*_ST_** |
| --- | --- | --- | --- |
| SBR x KSN | 0.048* | 0.046* | 0.007 |
| SBR x NYK | 0.093 | 0.080 | 0.168 |
| KSN x NYK | 0.057 | 0.043* | 0.113 |

**p* value < 0.05.

**^§^**SBR, Sing Buri; KSN, Kalasin; NYK, Nakhon Nayok.
