# Supplementary material for: Questioning inbreeding: Could outbreeding affect productivity in the North African catfish in Thailand?
Source: PLoS One. 2024 May 6;19(5):e0302584. doi: 10.1371/journal.pone.0302584 (PMC11073742; doi:10.1371/journal.pone.0302584)
Supplement: S15 Table — (DOCX) [file pone.0302584.s015.docx]

**S15 Table.** Analysis of molecular variance (AMOVA) results for the North African catfish (*Clarias gariepinus*) based on 15 microsatellite loci.

| **Source of variation** | **df** | **Sum of squares** | **Variance components** | **Percentage of variation** |
| --- | --- | --- | --- | --- |
| among populations | 2 | 58.642 | 0.357 | 6 |
| among individual | 133 | 1085.111 | 2.449 | 40 |
| within individual | 136 | 443.500 | 3.261 | 54 |
| Total | 271 | 1587.254 | 6.067 | 100 |
