# Supplementary material for: Questioning inbreeding: Could outbreeding affect productivity in the North African catfish in Thailand?
Source: PLoS One. 2024 May 6;19(5):e0302584. doi: 10.1371/journal.pone.0302584 (PMC11073742; doi:10.1371/journal.pone.0302584)
Supplement: S16 Table — (DOCX) [file pone.0302584.s016.docx]

**S16 Table.** Nei’s genetic distance (*D*) values between three populations of the North African catfish (*Clarias gariepinus*).

| **Nei’s *D****** | SBR | KSN | NYK |
| --- | --- | --- | --- |
| SBR | 0.000 |  |  |
| KSN | 0.293 | 0.000 |  |
| NYK | 0.447 | 0.236 | 0.000 |

*SBR, Sing Buri; KSN, Kalasin; NYK, Nakhon Nayok.
