# Supplementary material for: Questioning inbreeding: Could outbreeding affect productivity in the North African catfish in Thailand?
Source: PLoS One. 2024 May 6;19(5):e0302584. doi: 10.1371/journal.pone.0302584 (PMC11073742; doi:10.1371/journal.pone.0302584)
Supplement: S17 Table — (DOCX) [file pone.0302584.s017.docx]

**S17 Table.** Wilcoxon sign rank test to evaluate mutation–drift equilibrium in 136 North African catfish (*Clarias gariepinus*) under different models.

| **Locality*** | **Wilcoxon test** | | **Mode-shift test** |
| --- | --- | --- | --- |
|  | **TPM** | **SMM** |  |
|  | **Probability for one tail test for *H* excess** | **Probability for one tail test for *H* excess** |  |
| SBR | 0.013 | 0.555 | normal L-shaped distribution |
| KSN | 0.000 | 0.906 | normal L-shaped distribution |
| NYK | 0.000 | 0.805 | normal L-shaped distribution |

*SBR, Sing Buri; KSN, Kalasin; NYK, Nakhon Nayok.

TPM, two-phased model of mutation; SMM, stepwise mutation model.
