# Supplementary material for: Questioning inbreeding: Could outbreeding affect productivity in the North African catfish in Thailand?
Source: PLoS One. 2024 May 6;19(5):e0302584. doi: 10.1371/journal.pone.0302584 (PMC11073742; doi:10.1371/journal.pone.0302584)
Supplement: S18 Table — (DOCX) [file pone.0302584.s018.docx]

**S18 Table.** The mean migration rates between populations and 95% confidence intervals for standard deviation determined via BayesAss using microsatellite data for North African catfish (*Clarias gariepinus*).

| **Direction of migration*** | **Posterior mean of migration rates** | **Standard deviation** |
| --- | --- | --- |
| SBR→SBR | 0.930 | 0.043 |
| KSN→SBR | 0.035 | 0.033 |
| NYK→SBR | 0.035 | 0.031 |
| SBR→KSN | 0.006 | 0.005 |
| KSN→KSN | 0.981 | 0.010 |
| NYK→KSN | 0.013 | 0.008 |
| SBR→NYK | 0.011 | 0.010 |
| KSN→NYK | 0.014 | 0.013 |
| NYK→NYK | 0.976 | 0.016 |

*SBR, Sing Buri; KSN, Kalasin; NYK, Nakhon Nayok.
