# Supplementary material for: Questioning inbreeding: Could outbreeding affect productivity in the North African catfish in Thailand?
Source: PLoS One. 2024 May 6;19(5):e0302584. doi: 10.1371/journal.pone.0302584 (PMC11073742; doi:10.1371/journal.pone.0302584)
Supplement: S19 Table — (DOCX) [file pone.0302584.s019.docx]

**S19 Table.** Mutation-scaled effective population sizes (Θ) in three populations of the North African catfish and asymmetric migration rates (*M*) between populations among 136 North African catfish (*Clarias gariepinus*) for the estimated with 15 microsatellite loci.

| **Parameter*** | **2.50%** | **25.00%** | **Mode** | **75.00%** | **97.50%** |
| --- | --- | --- | --- | --- | --- |
| population size (Θ) |  |  |  |  |  |
| SBR | 0.090 | 0.096 | 0.098 | 0.099 | 0.100 |
| KSN | 0.088 | 0.096 | 0.098 | 0.100 | 0.100 |
| NYK | 0.087 | 0.094 | 0.096 | 0.097 | 0.100 |
| immigration rate (*M*) |  |  |  |  |  |
| KSN→SBR | 0.000 | 5.333 | 12.333 | 19.333 | 30.667 |
| NYK→SBR | 60.667 | 86.667 | 105.667 | 120.667 | 130.667 |
| SBR→KSN | 0.000 | 5.333 | 13.000 | 20.000 | 32.000 |
| NYK→KSN | 90.667 | 112.000 | 121.667 | 130.000 | 152.667 |
| SBR→NYK | 0.000 | 11.333 | 28.333 | 38.000 | 60.000 |
| KSN→NYK | 7.333 | 18.000 | 26.333 | 34.000 | 46.000 |

*SBR, Sing Buri; KSN, Kalasin; NYK, Nakhon Nayok.
