# Supplementary material for: Questioning inbreeding: Could outbreeding affect productivity in the North African catfish in Thailand?
Source: PLoS One. 2024 May 6;19(5):e0302584. doi: 10.1371/journal.pone.0302584 (PMC11073742; doi:10.1371/journal.pone.0302584)
Supplement: S20 Table — (DOCX) [file pone.0302584.s020.docx]

**S20 Table.** The effective number of immigrants (*N*_m_) from population i into population j per generation in three populations of the North African catfish (*Clarias gariepinus*) estimated with 15 microsatellite loci.

| **Population*** | **SBR** | **KSN** | **NYK** |
| --- | --- | --- | --- |
| SBR |  | 0.320 | 0.677 |
| KSN | 0.302 |  | 0.629 |
| NYK | 2.591 | 2.992 |  |

*SBR, Sing Buri; KSN, Kalasin; NYK, Nakhon Nayok.
