# Supplementary material for: Questioning inbreeding: Could outbreeding affect productivity in the North African catfish in Thailand?
Source: PLoS One. 2024 May 6;19(5):e0302584. doi: 10.1371/journal.pone.0302584 (PMC11073742; doi:10.1371/journal.pone.0302584)
Supplement: S21 Table — (DOCX) [file pone.0302584.s021.docx]

**S21 Table.** Mutation-scaled effective population sizes (Θ) in three populations of the North African catfish (*Clarias gariepinus*) and asymmetric migration rates (*M*) between populations estimated with the mitochondrial DNA D-loop sequences.

| **Parameter*** | **2.50%** | **25.00%** | **Mode** | **75.00%** | **97.50%** |
| --- | --- | --- | --- | --- | --- |
| population size (Θ) |  |  |  |  |  |
| SBR | 0.093 | 0.097 | 0.098 | 0.099 | 0.100 |
| KSN | 0.092 | 0.097 | 0.098 | 0.099 | 0.100 |
| NYK | 0.080 | 0.094 | 0.098 | 0.100 | 0.100 |
| immigration rate (*M*) |  |  |  |  |  |
| KSN→SBR | 0.000 | 0.000 | 3.700 | 9.300 | 20.000 |
| NYK→SBR | 0.000 | 4.700 | 11.700 | 18.000 | 27.300 |
| SBR→KSN | 0.000 | 1.300 | 7.000 | 12.700 | 23.300 |
| NYK→KSN | 55.300 | 68.700 | 77.700 | 86.000 | 100.000 |
| SBR→NYK | 0.000 | 5.300 | 13.000 | 19.300 | 29.300 |
| KSN→NYK | 108.700 | 127.300 | 139.000 | 149.300 | 168.700 |

*SBR, Sing Buri; KSN, Kalasin; NYK, Nakhon Nayok.
