# Supplementary material for: Questioning inbreeding: Could outbreeding affect productivity in the North African catfish in Thailand?
Source: PLoS One. 2024 May 6;19(5):e0302584. doi: 10.1371/journal.pone.0302584 (PMC11073742; doi:10.1371/journal.pone.0302584)
Supplement: S22 Table — (DOCX) [file pone.0302584.s022.docx]

**S22 Table.** The effective number of immigrants (*N*_m_) from population i into population j per generation in three populations of the North African catfish (*Clarias gariepinus*) estimated by the mitochondrial DNA D-loop sequences.

| **Population* (i\j)** | **SBR** | **KSN** | **NYK** |
| --- | --- | --- | --- |
| SBR |  | 0.172 | 0.319 |
| KSN | 0.091 |  | 3.416 |
| NYK | 0.288 | 1.909 |  |

*SBR, Sing Buri; KSN, Kalasin; NYK, Nakhon Nayok.
