# Supplementary figures and images for: Questioning inbreeding: Could outbreeding affect productivity in the North African catfish in Thailand?
Source: PLoS One. 2024 May 6;19(5):e0302584. doi: 10.1371/journal.pone.0302584 (PMC11073742; doi:10.1371/journal.pone.0302584)

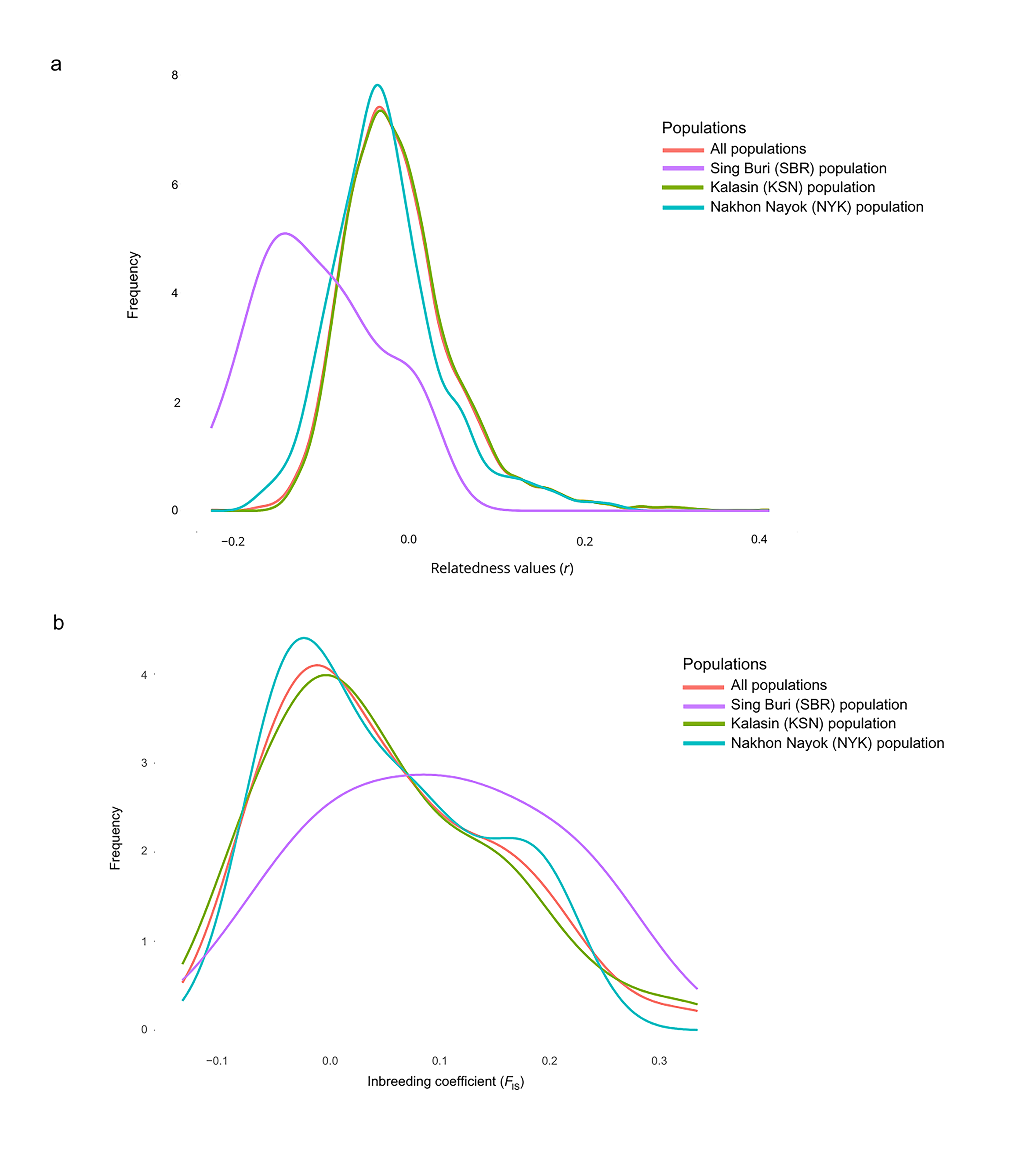

Supplement: S1 Fig — (A) pairwise relatedness values (r). (B) inbreeding coefficients (FIS). (TIF) [file pone.0302584.s023.tif]

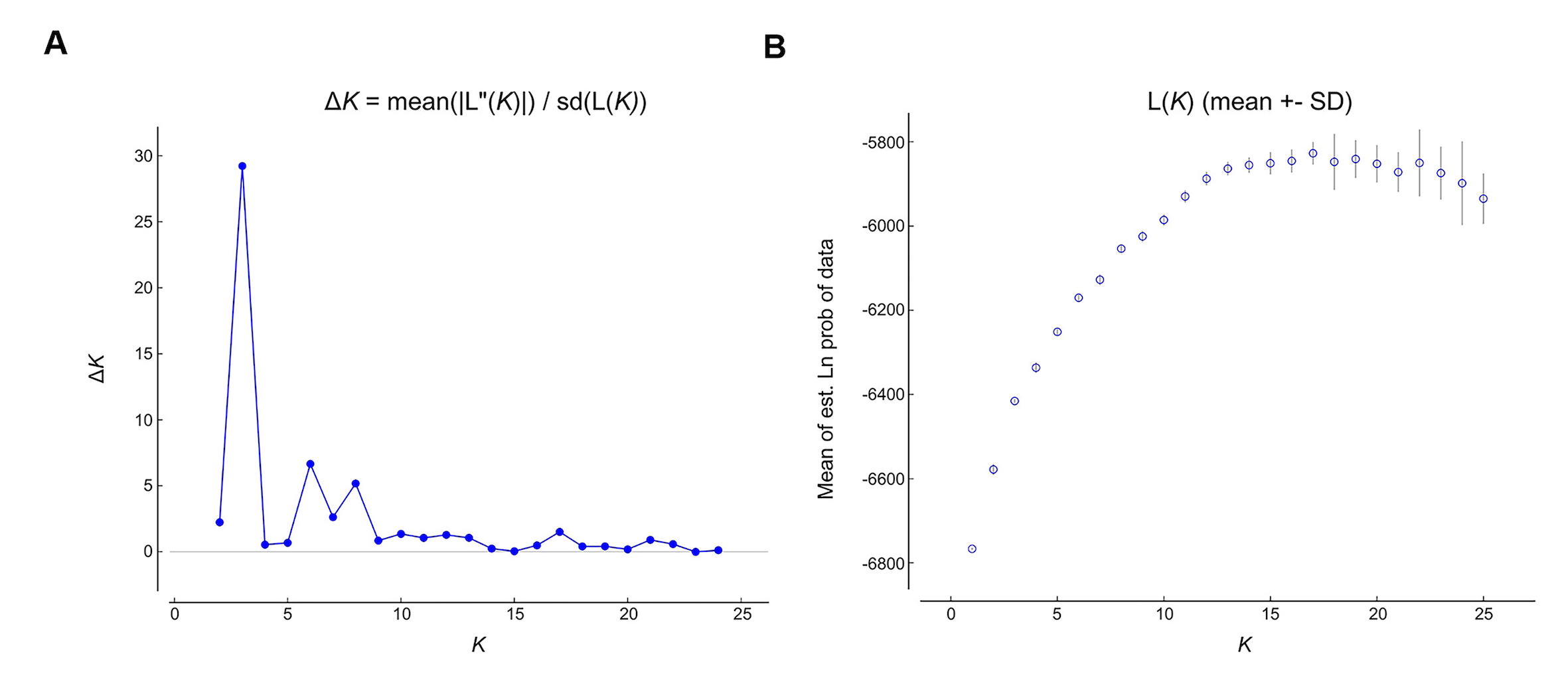

Supplement: S2 Fig — Plot of (A) Evanno’s ΔK and (B) ln P(K). (TIF) [file pone.0302584.s024.tif]

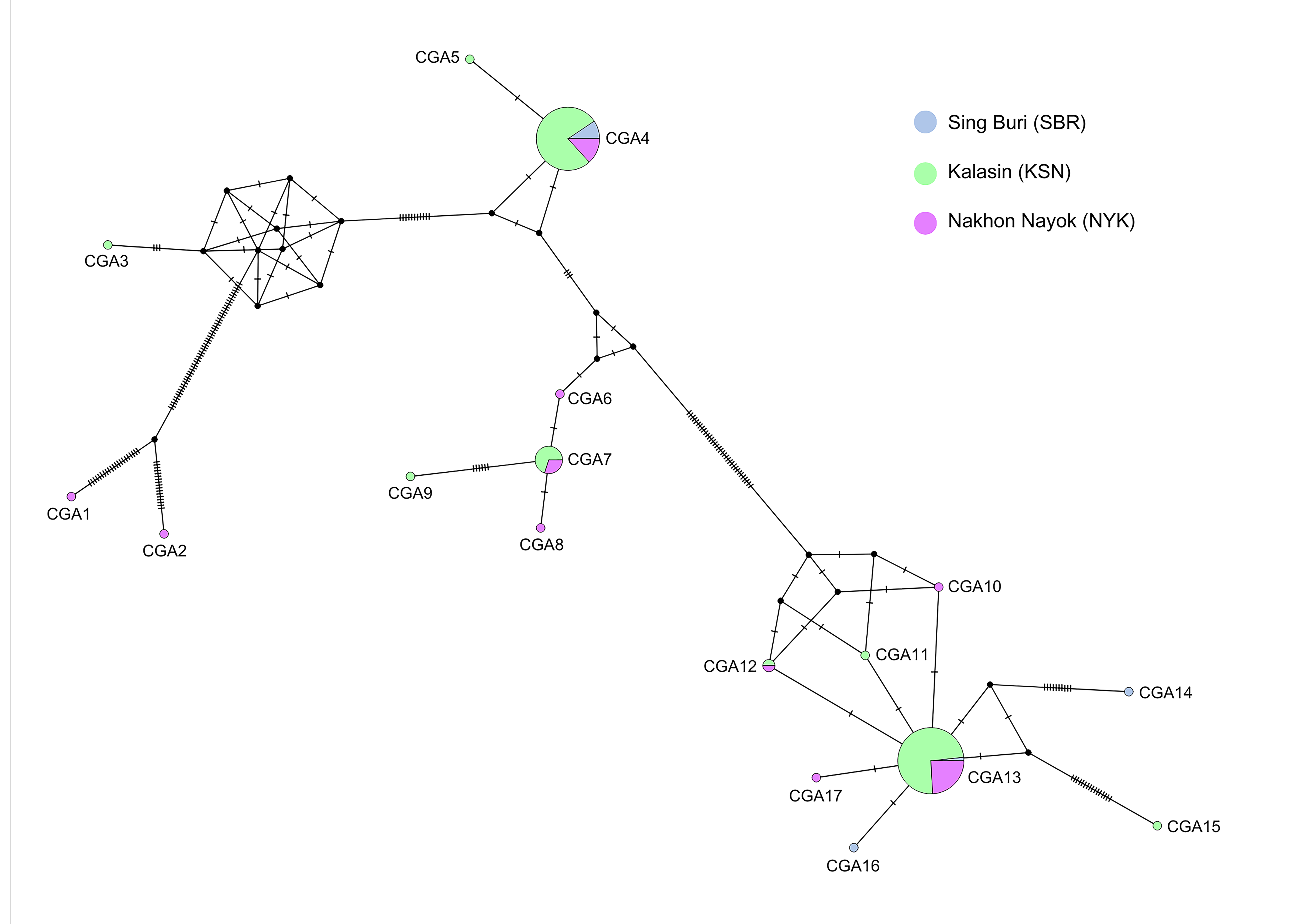

Supplement: S3 Fig — SBR, Sing Buri population,KSN, Kalasin population,NYK, Nakorn Nayok population. (TIF) [file pone.0302584.s025.tif]

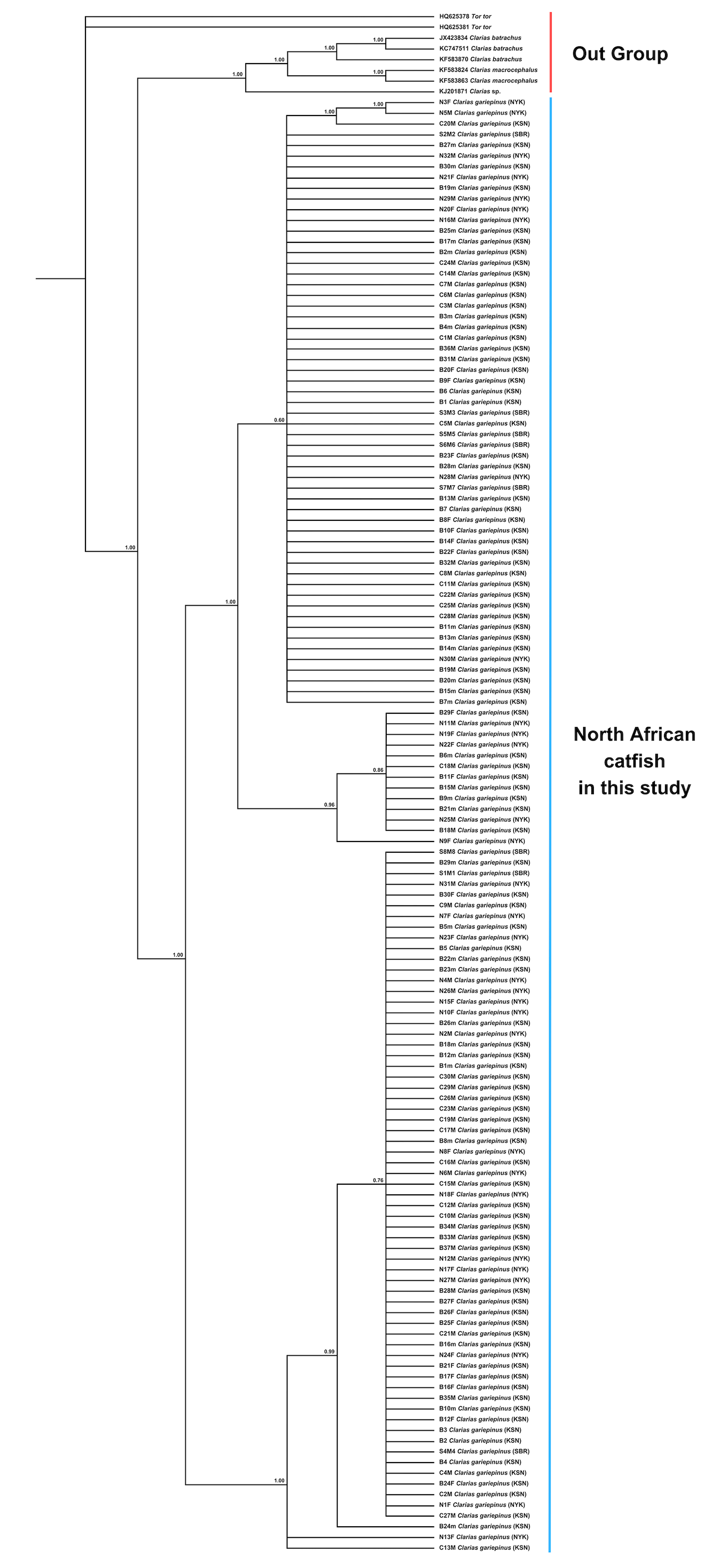

Supplement: S4 Fig — The two most common haplotypes (CGA4 and CGA13) were shared among three populations. (TIF) [file pone.0302584.s026.tif]

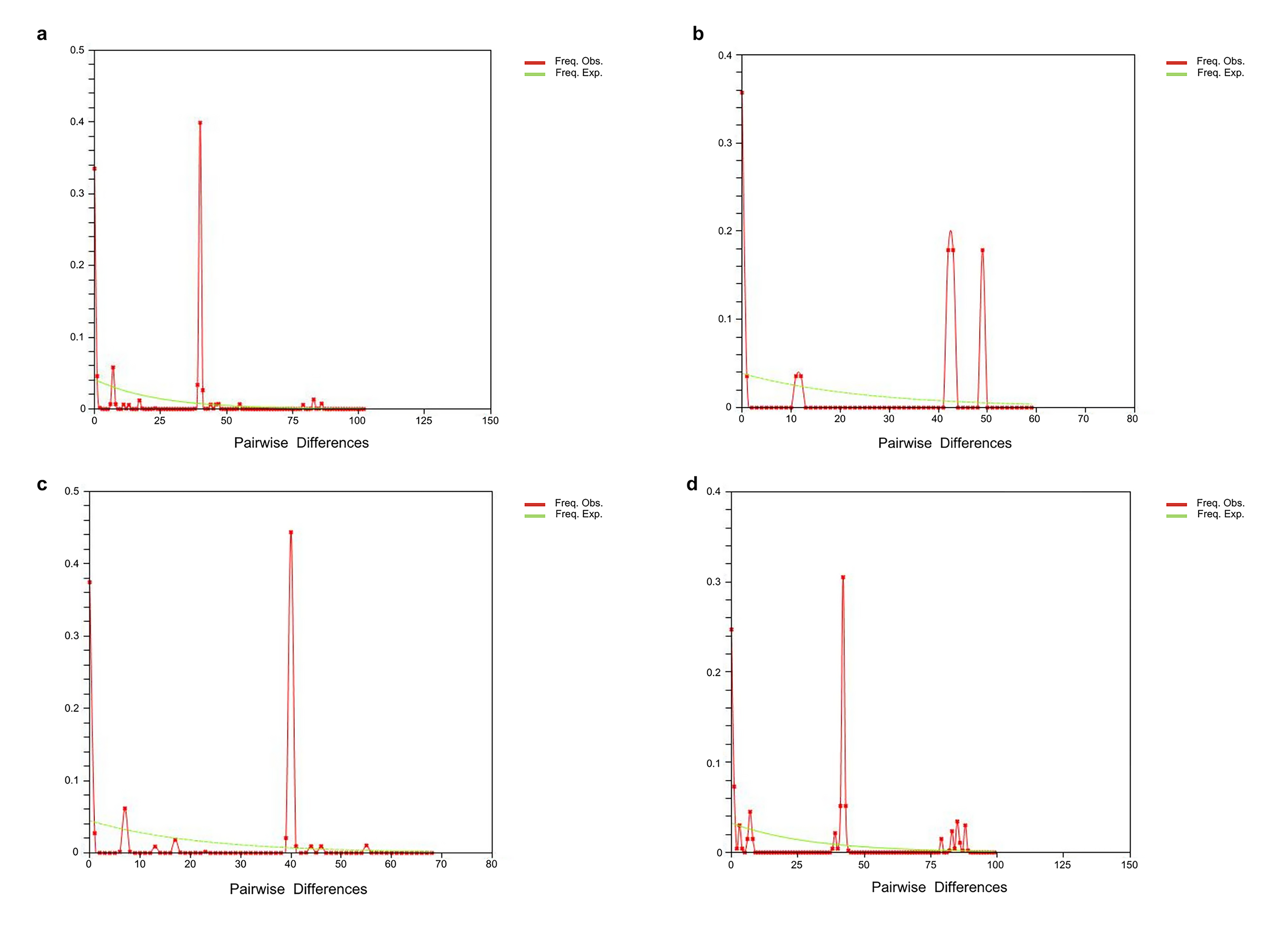

Supplement: S5 Fig — (A) the entire population, (B) the Sing Buri population, (C) the Kalasin population, (D) the Nakorn Nayok population. The x-axis represents the number of pairwise differences (mismatches), and the y-axis represents the frequency of these differences. The distribution of frequencies of observed mismatches (red line) is compared to those of frequencies of expected mismatches (green line). (TIF) [file pone.0302584.s027.tif]

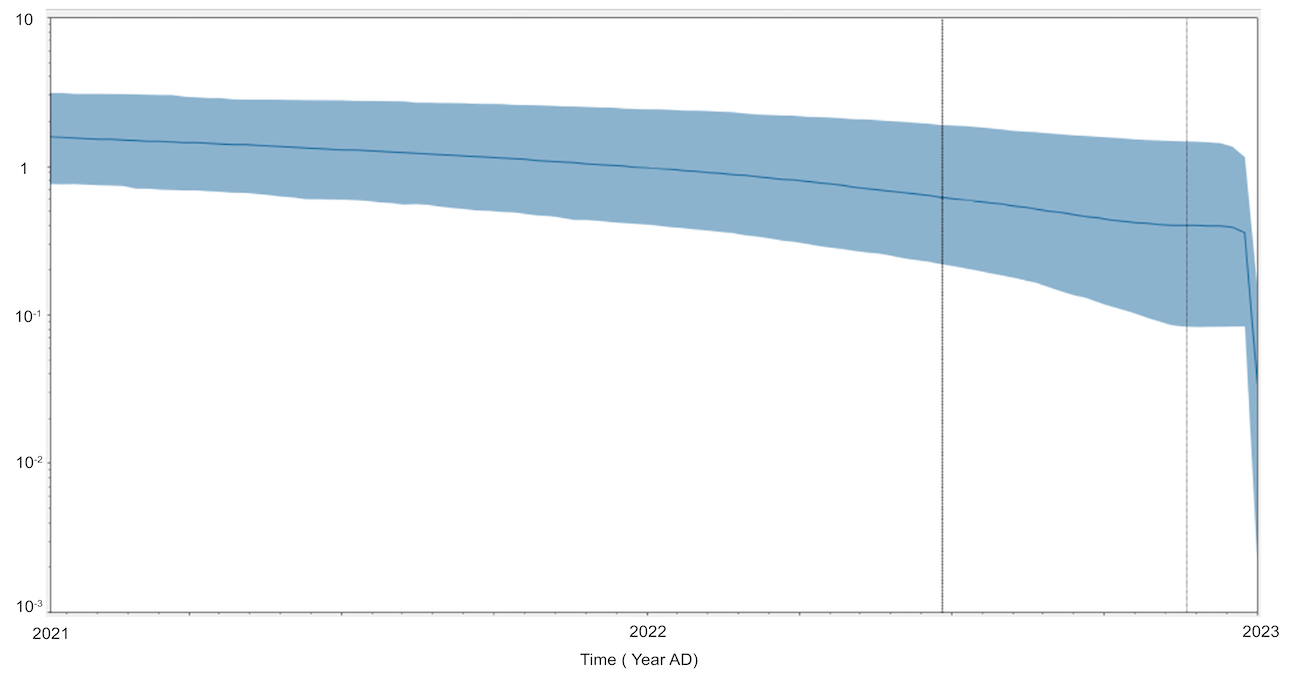

Supplement: S6 Fig — The median effective population size is delimited by the black lines. The blue shaded area delimits the upper and lower bounds of the 95% highest posterior density interval. The x-axis represents time in years and the y-axis is displayed in logarithmic scale. (TIF) [file pone.0302584.s028.tif]
